# Meet our European editorial board members

**DOI:** 10.1111/iwj.70085

**Published:** 2024-11-18

**Authors:** Douglas Queen, Keith Harding

**Affiliations:** ^1^ International Wound Journal Oxford UK

In our recent editorials, we discussed the expansion of our Editorial Board^1^, ^2^ and introduced you to some of our members. We promised a series of further editorials introducing our wider membership. This is the second in a series of three editorials providing insight into the group of outstanding and distinguished individuals that comprise our board. We have created the largest most internationally diverse board to greatly increase the capabilities and expertise of the journal.

## 1 EUROPEAN EDITORIAL BOARD MEMBERS

Please meet the editorial board members for the International Wound Journal covering Europe.

**Table:** 

**Mr. Naseer Ahmad, UK**	
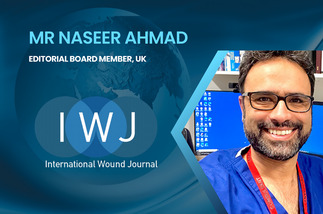	Naseer is a vascular surgeon based at Manchester University Foundation Trust. He performs the full range of vascular procedures including carotid, aneurysm and venous surgery but specializes in complex lower limb surgery that prevents an amputation. He is the Clinical Director of both the Manchester Amputation Reduction Strategy (The MARS Project) and the Greater Manchester & East Cheshire Aneurysm Screening Programme and a former Deputy Chief Clinical Information Officer of his hospital. His award winning research interest is the inequality surrounding lower limb amputations, which led to developing The MARS Project. This project has resulted in a 42% reduction in amputation number over 6 years in a pilot site of 220 000 people. This work is currently being scaled up for 3 million people. His cross‐sectoral roles are now enabling him to develop ‘whole systems’ pathways, that is, those that harmonize Public Health, community and hospital services for patients with vascular disease. His aim is to bring together and digitize siloed NHS services and to work with leaders across Manchester's academic, clinical, strategic, financial and digital areas to drive this forward.
**Dr. Kirsti Ahmajärvi, Finland**	
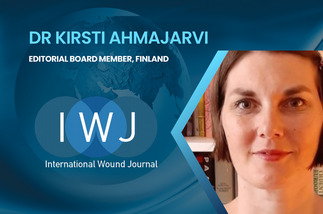	A licensed physician since 2001, Kirsti graduated from the University of Oulu, Finland. She specialized in General medicine (2014) and Geriatrics (2024) at the University of Helsinki. She also has a special competence in wound management (for physicians), which was approved by Finnish Medical Association in 2016. As president of the working group of special competence in wound care, she is developing wound care at a national level by developing this special competence for physicians. As a general practitioner in a primary care out‐patient clinic, she also worked in a wound care ward. During her specialization, she worked in several hospitals, including the emergency room and specialist out‐patient clinics. Kirsti is currently a doctoral student in Helsinki University since 2016 on the subject of chronic wounds, diagnostic delay and primary care. She has several publications on this subject. Kirsti is also a Trustee in the European Pressure Ulcer Advisory Panel (EPUAP), a group member on EWMA's GP network and was a board member of the Finnish Wound Care Society (2017–2019). She participated in several working groups of Helsinki University Hospital Wound Center during 2017–2020, and was a board member of the Finnish National Group of Nordic Diabetic Foot since 2016. Kirsti is very much interested in developing wound care especially in primary care.
**Maarit Ahtiala, Finland**	
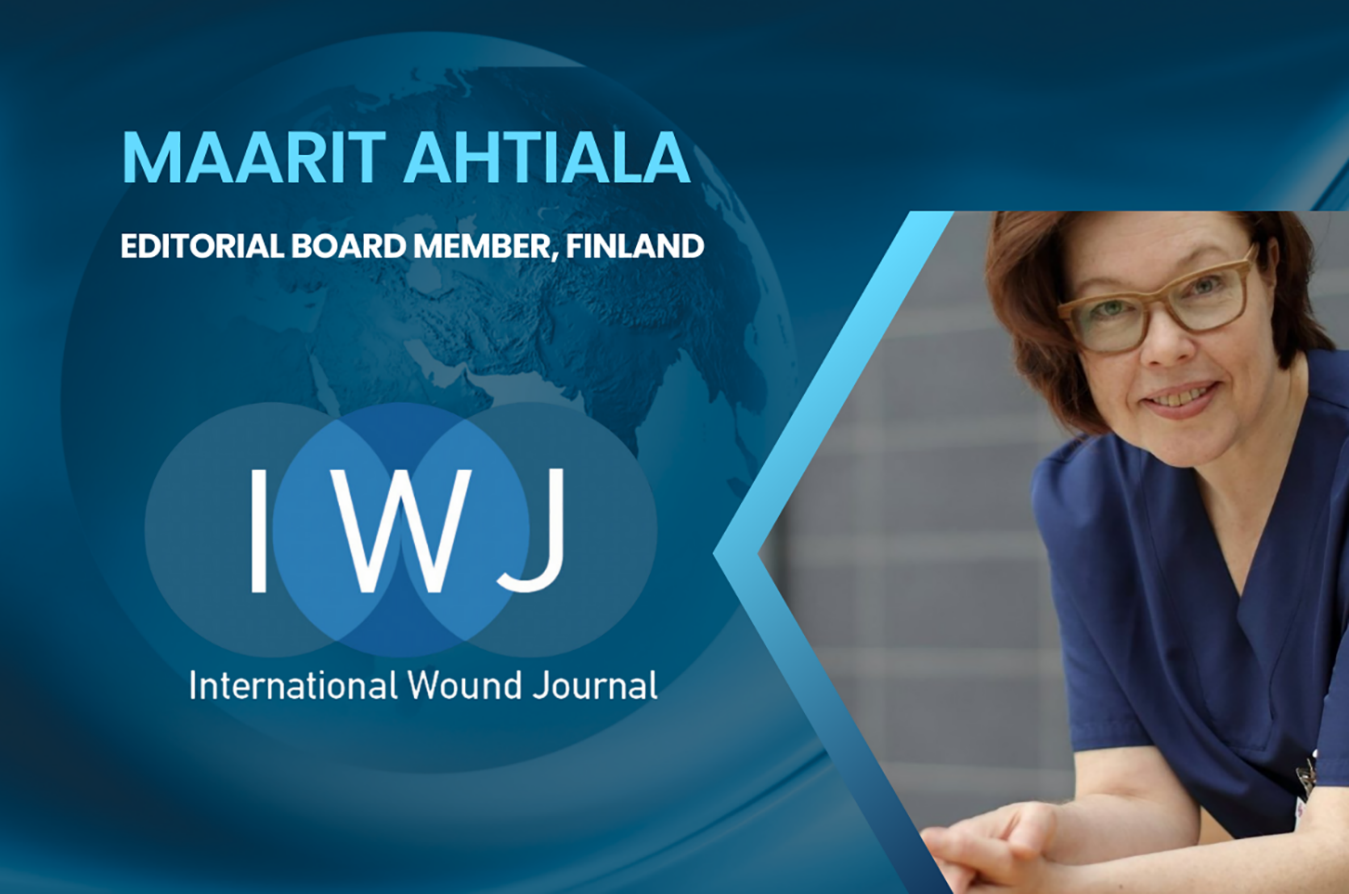	Maarit is a trained wound care nurse within the ICU at Turku University Hospital for over 26 years. Responsibilities include coordination of wound care and pressure ulcer prevention practices within the department. Member (2002‐present>) and secretary (2006–2020) of Turku University Hospital Wound Care team. Maarit has worked on many guidelines (EPUAP, NPIAP and PPPIA International guideline 2015 and 2019; Finnish PU prevention Guideline Working Group 2015 and 2023) and organized the translation of the EPUAP, NPIAP and PPPIA. Maarit was involved in the publication of ‘Prevention and Treatment of Pressure Ulcers: Quick Reference Guide’. Emily Haesler (Ed). Cambridge Media: Perth, Australia; 2014 and 2019. Maarit was the Chair of the 4th EPUAP Focus Meeting 2018 and will be the Chair of the 25th EPUAP Annual Meeting 2025. She is a frequent lecturer on pressure ulcer prevention, classification and treatment within her own hospital, hospital district and across Finland. She also lectures on other wound‐related topics including wound debridement and wound dressing choice. She is also a frequently invited speaker at international educational events and conferences. Marrit is the Principal investigator; Pressure ulcers in ICU research program in Turku University Hospital, since 2010. The research focuses on the risk assessment methods, pathophysiological factors and occurrence of pressure ulcers in intensive care. From this research, nine international peer‐reviewed articles have been published. Her other publications include several PU and wound care related publications in international journals, Finnish Intensive Care and Finnish Wound Journals. She has authored and co‐authored chapter in national books and e‐publications published by The Finnish Medical Society Duodecim. She has been a member of many societies including EPUAP, EPUAP Trustee (2014–2020, 2021‐); Member of the Guideline Committee (Translations) EWMA (2014‐); Finnish Wound Care Society; and the Finnish Society of Intensive Care.
**Professor Ahmet Altintas, Germany**	
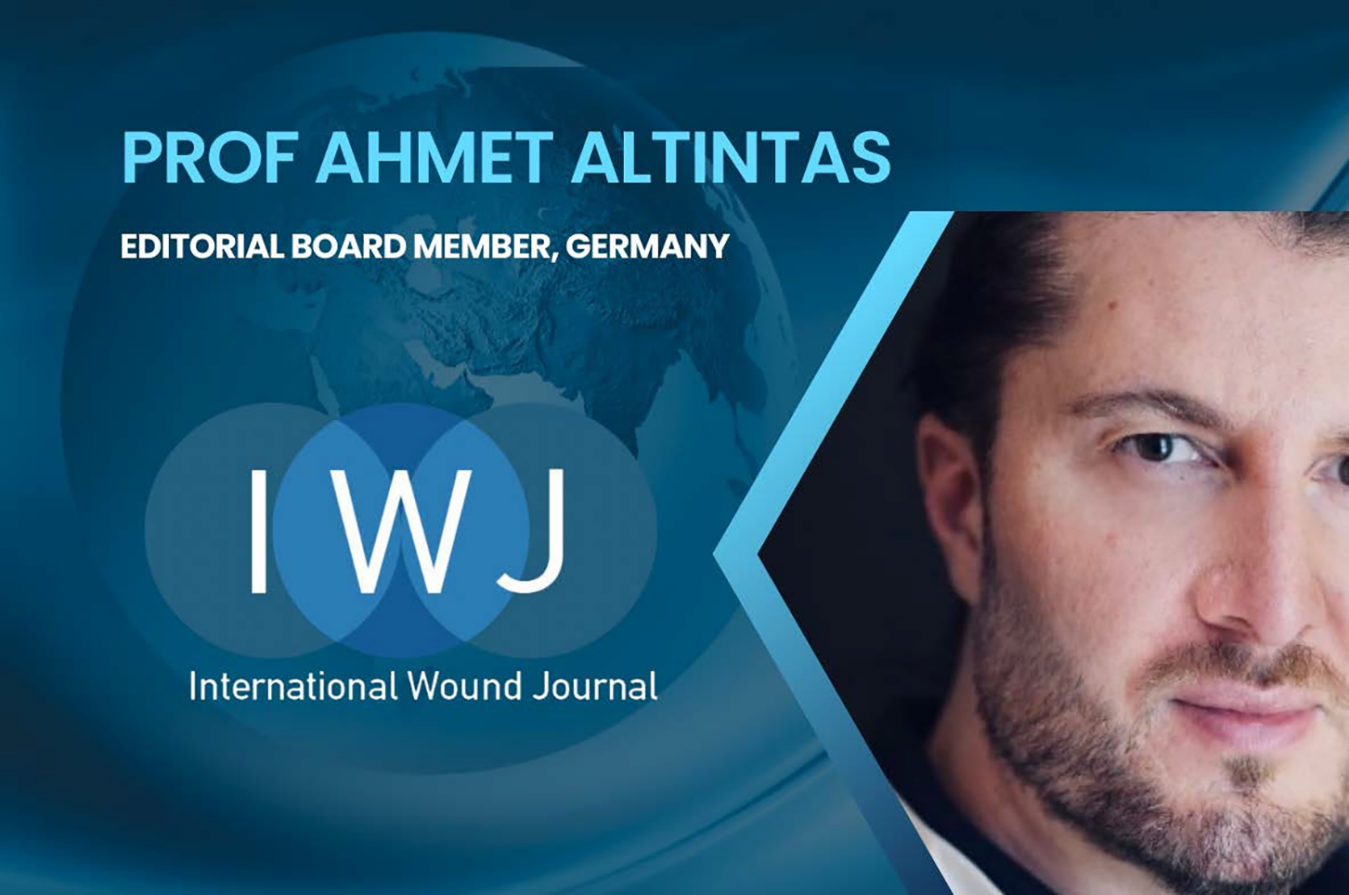	Ahmet is Professor for Plastic surgery in Germany. He graduated from Ruhr University Bochum, Germany. His Doctoral thesis was about ‘Tendon Transfer for Radial nerve Palsy’ at the BG Trauma Center Ludwigshafen, Department of Plastic and Hand Surgery at the Ruperto Carola University of Heidelberg, Heidelberg, Germany. Ahmet also has a Master's degree (MSc) in Public health from Ruperto Carola University in Heidelberg. His surgical career started at the general surgery at the University Heidelberg, followed by German Center of Trauma Surgery at the Hannover Medical School. He worked at the Department of Plastic Surgery, Burn and Hand Surgery Center, Cologne Merheim, Germany. Altintas hold board certification for Plastic and Aesthetic Surgery. He was Senior Consultant at the Department of Plastic and Hand Surgery at the University of Essen, Essen, Germany, and since 2011, he has been an Vice Director of the Department of Plastic Surgery Diakonische Kliniken Hannover. Dr. Altintas is a member of the editorial board in numerous Journals in Plastic and Aesthetic Surgery such as Advanced in Wound care (impact Factor 5.2). He presented more than 80 papers at national and international scientific meetings. He has published more than 50 peer reviewed papers in scientific journals and various book chapters. His research focuses on Aesthetic Surgery, Reconstructive plastic procedures and wound healing.
**Professor Paulo Alves, Portugal**	
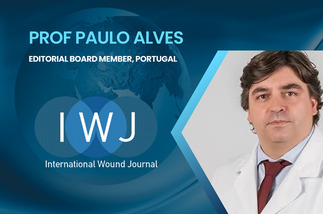	Paulo Alves is a researcher in the field of nursing and wound care, known for his substantial contributions and achievements. Completing his PhD in Nursing at Universidade Católica Portuguesa in 2015, he currently holds prestigious positions as an Associate Professor and Dean at the same institution. His commitment to advancing the field is evident through his extensive research output, having published more than 60 articles in specialized journals and presenting at numerous international and national events. Paulo has a notable record of authoring 10 book chapters and 5 books and has registered a product, collectively contributing to over 200 technical production items. His dedication to the field extends beyond research, as he has actively participated in over 100 international and national conferences, highlighting his ongoing commitment to sharing knowledge and driving innovation. In his role as an educator and mentor, Paulo has successfully supervised various PhD and master's theses in nursing and co‐supervised also in other health sciences area. His involvement in more than 10 research projects between 2007 and 2023, along with his current engagement in two ongoing research projects, underscores his active role in the advancement of medical sciences, particularly in wound care. Paulo's collaborative nature is reflected in his professional activities, having worked with approximately 200 colleagues in co‐authorship of scientific works. Paulo Alves's work is characterized by a focus on nursing, oncology, skin, wound treatment, ulcers, wound care, prevention, teaching, education, healthcare and pressure ulcers. This diverse range of interests and expertise signifies his deep understanding and passion for improving patient outcomes and advancing the field of wound care. His dedication to innovation and research makes him an invaluable contributor to the future of medical sciences.
**Dr. Javier Aragon‐Sanchez, Spain**	
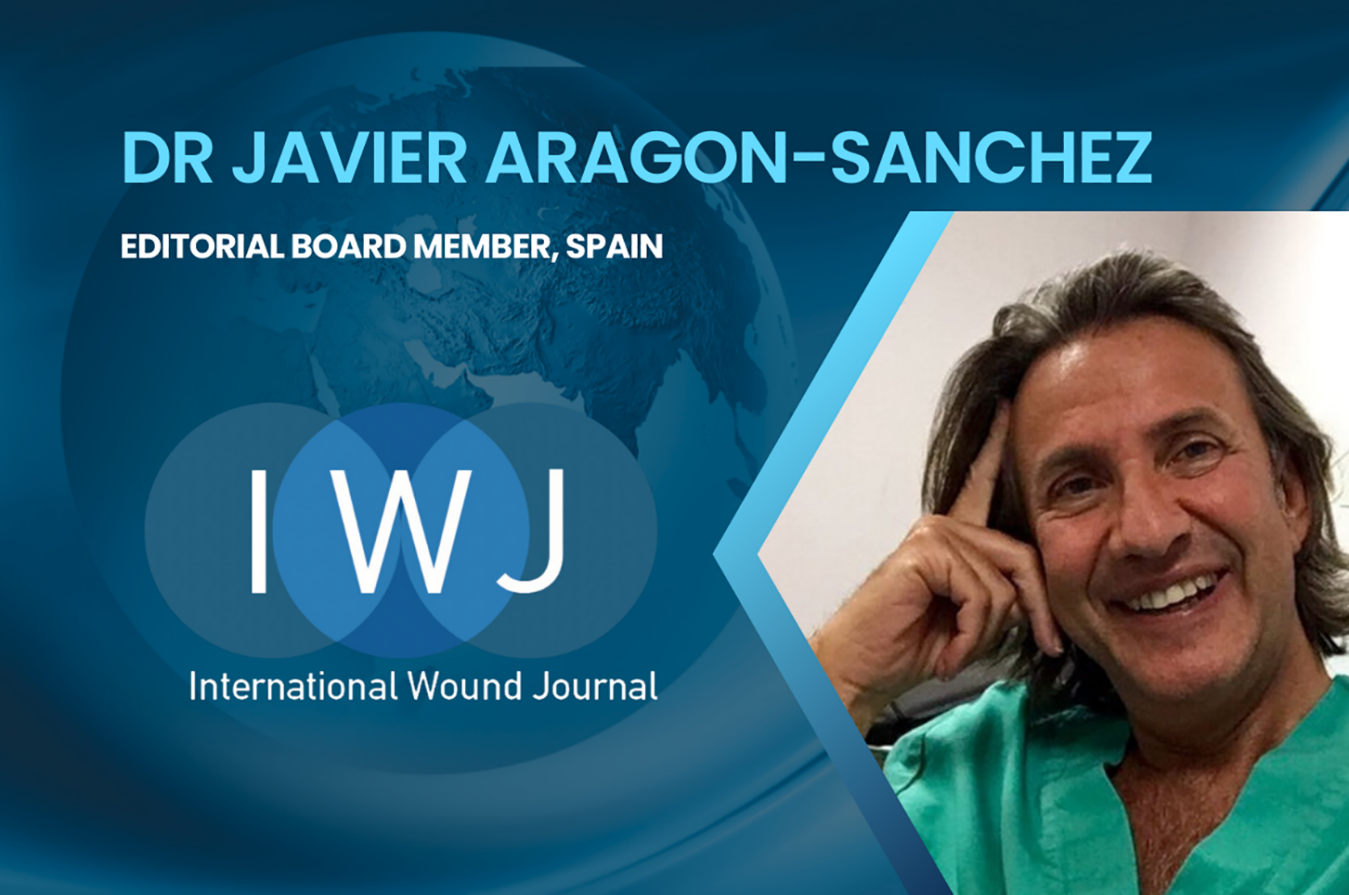	Dr. Javier Aragón‐Sánchez is a General Surgeon. He is the Head of Department of Surgery and Diabetic Foot Unit of La Paloma Hospital in Las Palmas de Gran Canaria (Spain). He founded in 1999 a specialized Diabetic Foot Unit in his hospital. He is the chairman of the Interdisciplinary Diabetic Foot Study Group in Spain and organized three National Interdisciplinary Diabetic Foot Meetings. He is founder partner of the Spanish Wound Healing Society. He is also founder member and spokesman of the Diabetic Foot Study Group on behalf the Spanish Diabetes Association and member of the European Diabetic Foot Study Group on behalf of EASD. He is corresponding member of the IWGDF working group on Infection 2011; assistant lecturer in the Health Science School of Las Palmas de Gran Canaria University and Nursing Department, Complutense University, Madrid. He is the author of two Spanish textbooks and several chapters in other textbooks and monographs dealing with diabetic foot problems and author of peer‐review papers.
**Dr. Leanne Atkin, UK**	
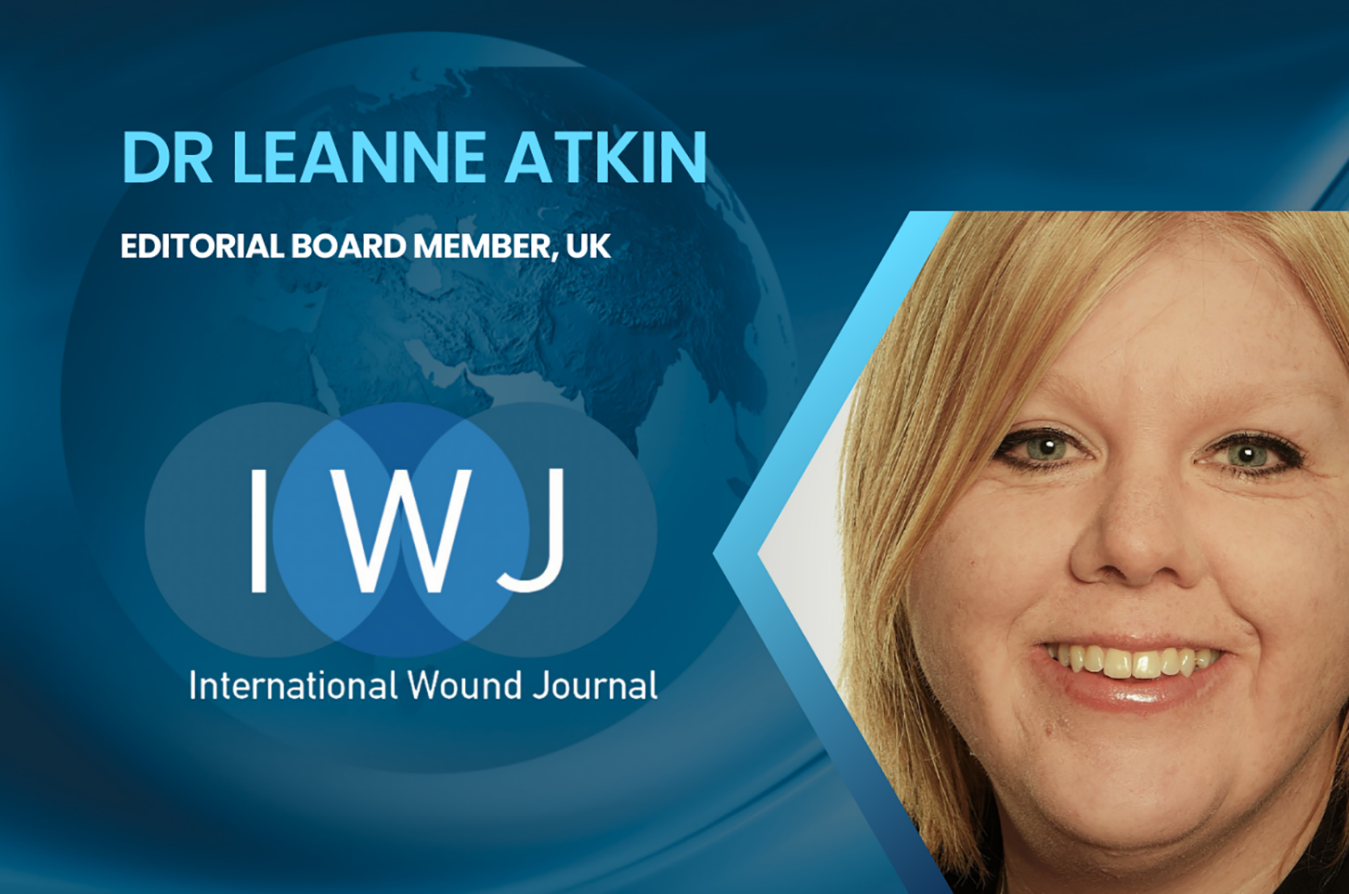	Dr. Leanne Atkin PhD MHSc RGN is a clinical academic with a joint appointment between the University of Huddersfield and Mid Yorkshire NHS Teaching Trust within the role of Vascular Nurse Consultant. Notable achievements mark her academic journey. In 2010, she obtained her master's degree in Advanced Nursing Practice, and in 2017, she completed a PhD, concentrating her research on the treatment and management of peripheral arterial disease. Driven by her dedication to improving patient outcomes, Dr. Atkin has channelled her expertise into various areas of interest. Her passions encompass leg ulcer management, advancing nursing practice, peripheral arterial disease and advanced wound management. This broad spectrum of interests reflects her holistic approach to patient care and research. With a publication record exceeding 100 articles, she has disseminated invaluable insights into lower limb ulceration, vascular diseases, wound assessment and quality of life. Her work not only informs clinical practice but also shapes the trajectory of research in her field. As an established researcher, Dr. Atkin undertakes pivotal roles as both chief and principal investigator in several studies. Her active engagement in research underscores her commitment to pushing the boundaries of knowledge and driving innovation in healthcare delivery. In addition to her scholarly pursuits, Dr. Atkin is deeply involved in advocacy initiatives aimed at raising awareness of lower limb conditions. She plays a vital role in the ‘Legs Matter’ campaign, leveraging her expertise to educate patients and the public about the importance of early detection and intervention in vascular health.
**Professor Dimitri Beeckman, Belguim**	
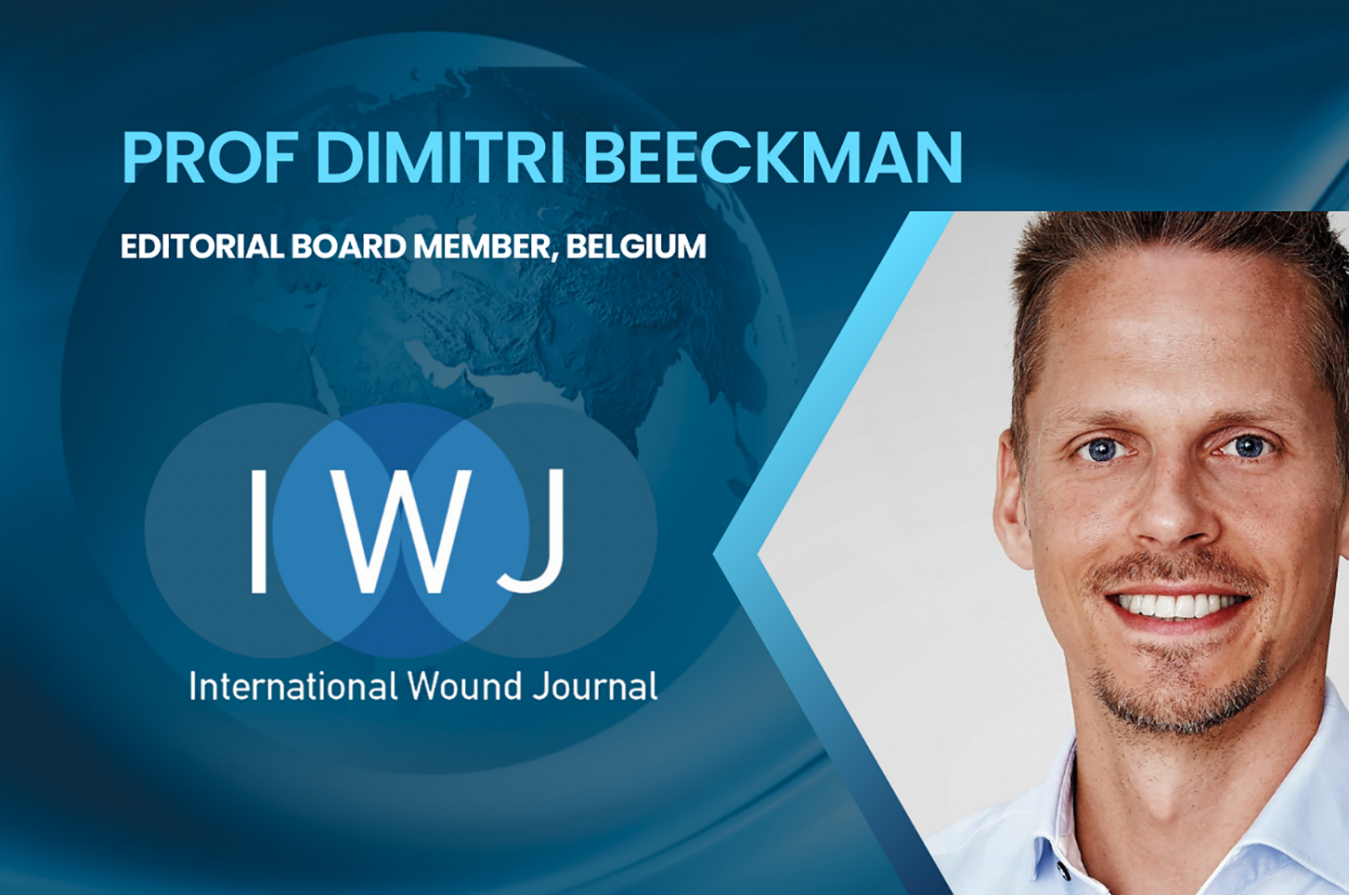	Prof. Dimitri Beeckman is a Professor of Skin Integrity and Clinical Nursing at Ghent University (Belgium). He holds visiting professorships at Örebro University (Sweden), University of Surrey (UK), Royal College of Surgeons in Ireland (Ireland), Monash University (Australia), and University of Southern Denmark (Denmark). He is an expert in skin integrity research, clinical trials and instrument development and validation. He authored over 120 scientific publications in international peer‐reviewed journals. He is Consulting Editor of the Journal of Wound, Ostomy and Continence Nursing, the Journal of Tissue Viability, as well as Associate Editor of BMC Geriatrics. He is President of the European Pressure Ulcer Advisory Panel (EPUAP), President Elect of the International Skin Tear Advisory Panel (ISTAP) and Council member of the European Wound Management Association (EWMA). He holds Fellowships of Sigma Theta Tau International Honour Society of Nursing and the European Academy of Nursing Science.
**Professor Thomas Bjarnsholt, Denmark**	
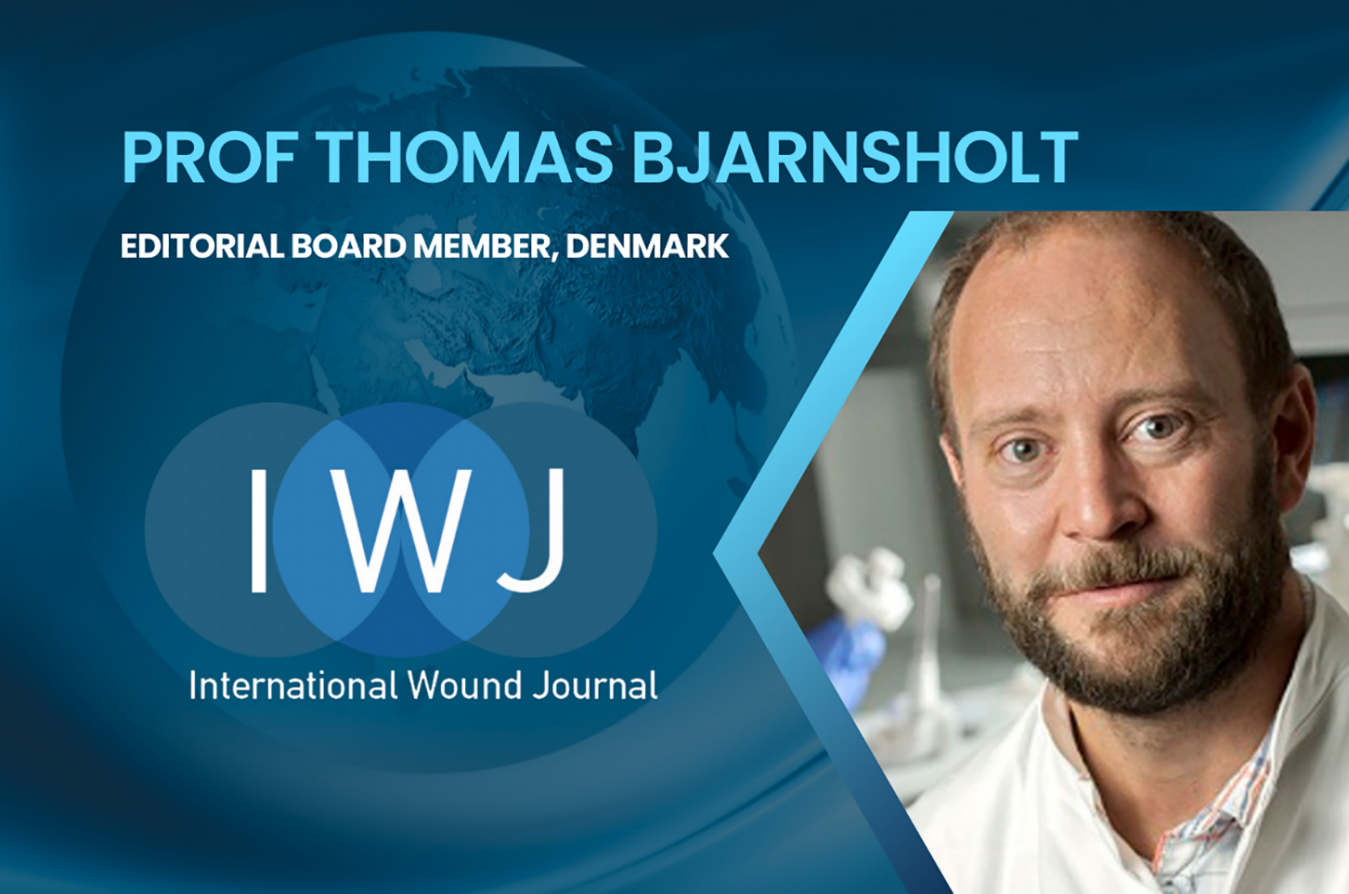	Prof Bjarnsholt is a global expert in the role of biofilms in chronic infections, with over 250 peer reviewed publications in the biofilm field including the ESCMID guidelines for biofilm treatment and Consensus guidelines for the identification and treatment of biofilms in chronic non‐healing wounds published in WRR. His research focuses on biofilm formation and the role of bacterial biofilm in chronic infections, both in vitro, animal models and ex vivo material from chronic infections. Bjarnsholt is interested as to how bacteria initiate biofilms in the human body and why the immune defence seems to fail both in the initial infection and later in the chronic infection. His research also seeks to develop tools and methods to enable fast diagnosis of these infections, for better treatment and possible prevention. For this, Bjarnsholt works in close collaboration with clinicians at most of the major hospitals in Denmark.
**Jonathan Brocklehurst, UK**	
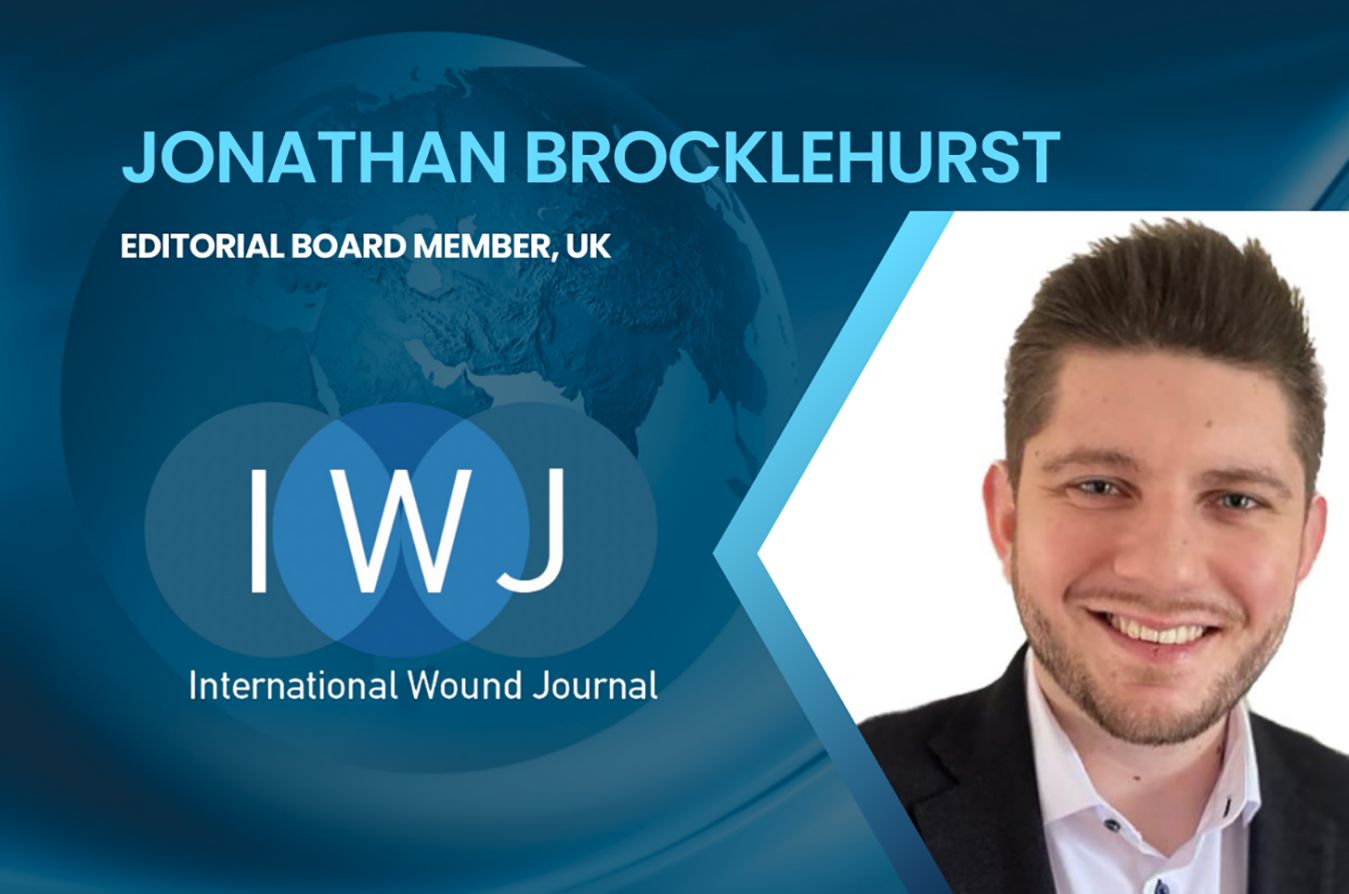	Jonathan is a Podiatrist based in Berkshire, United Kingdom and a member of the Royal College of Podiatry. His interest in Wound Care has blossomed from his clinical experience in the National Health Service, private practice and academic studies at the University of Southampton, and Cardiff University. Recently, a peer reviewer for the American Diabetes Association and lecturer with publications in Advances in Skin and Wound Care, Wounds UK, and the Journal of Foot and Ankle Research, Jonathan has developed a passion for research and continues to write on a broad range of topics within podiatric wound care.
**Professor Benjamin Bullen, UK**	
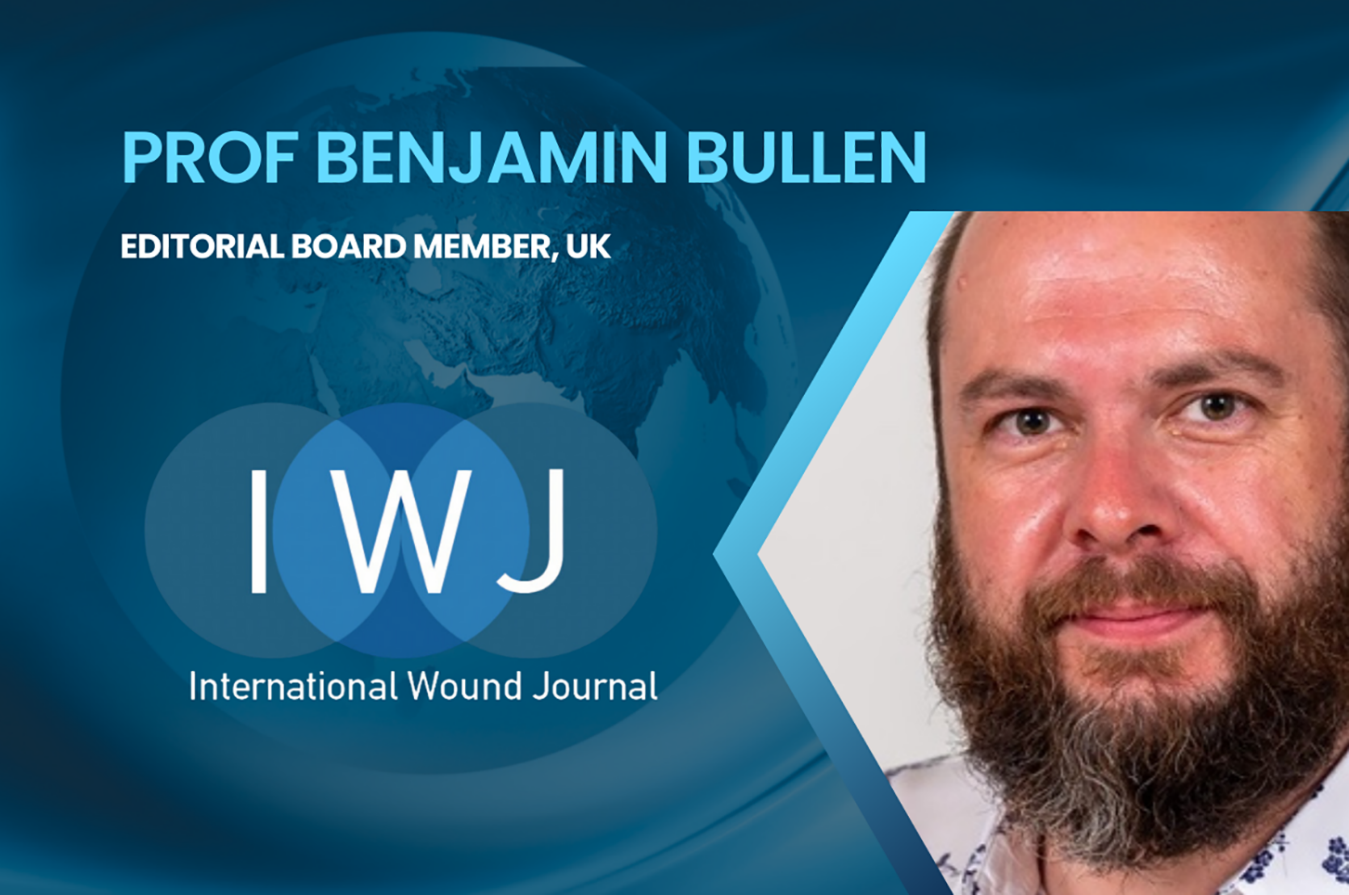	Benjamin graduated in 2001, from Curtin University of Technology, before contributing to multidisciplinary diabetes foot teams at The Royal Melbourne Hospital, Norfolk and Norwich University Hospital and The New Royal Infirmary of Edinburgh. Benjamin's Professional Doctorate in Person‐centred Practice explored Charcot foot health literacy among individuals with diabetic peripheral neuropathy. He also holds a Postgraduate Certificate in Wound Care, from Monash University, and a Postgraduate Diploma in Podiatry and Doctoral Certificate in Researcher Enhancement and Development, from Queen Margaret University. Benjamin has taught undergraduate and postgraduate podiatry students at Queen Margaret University and Cardiff Metropolitan University and pharmacy students at The University of Wolverhampton. He is currently a Lecturer in Podiatry at The University of Galway, Honorary Senior Lecturer at Cardiff University and Visiting Lecturer at Birmingham City University. He is a Chartered Scientist, Member of The Royal College of Podiatry and Council Member of Podiatry Ireland.
Professor Sergiu‐Bogdan Catrina, Sweden	
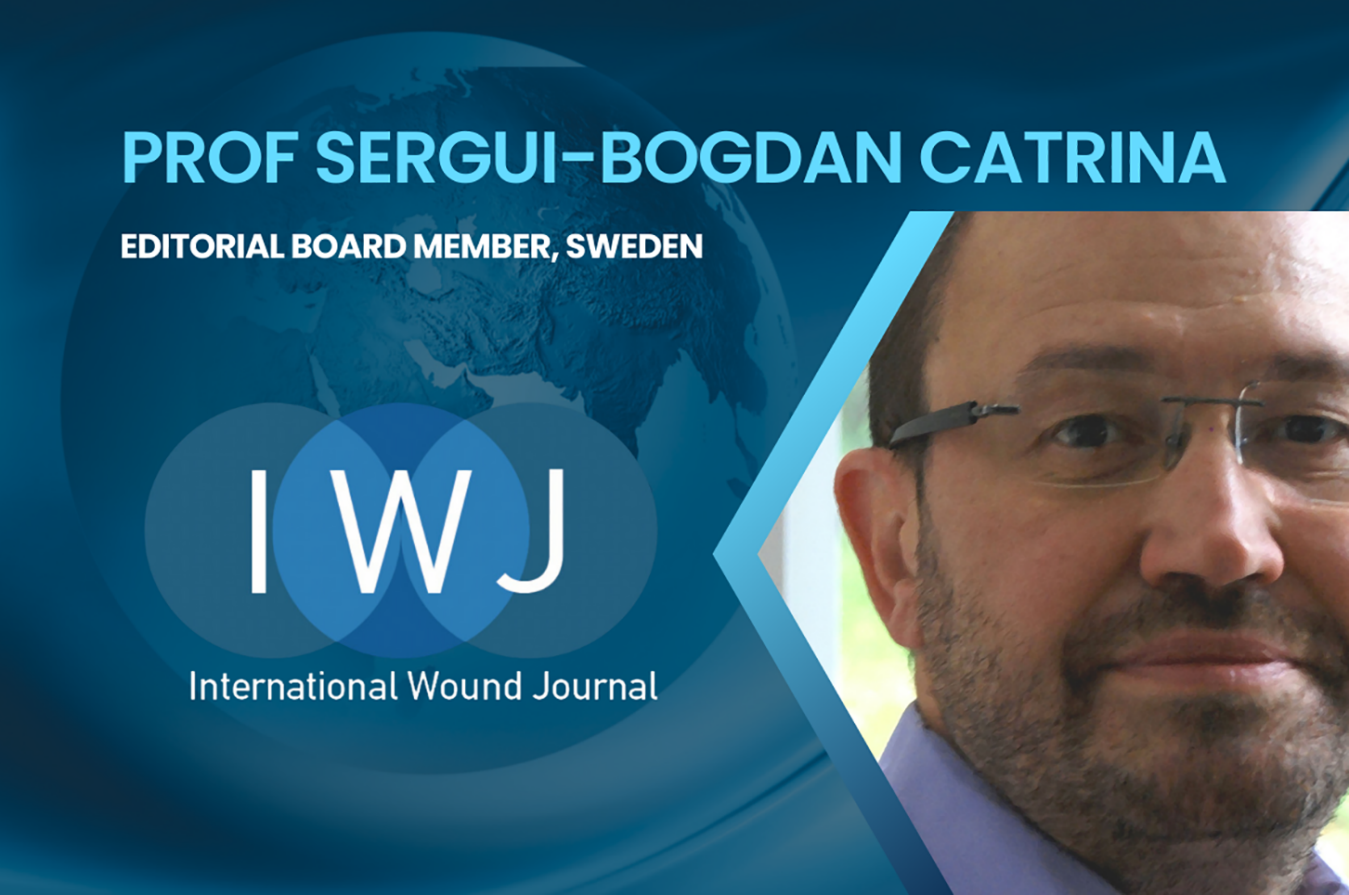	Sergiu‐Bogdan Catrina, consultant in Endocrinology at Central for diabetes in Stockholm and Associate Professor at Karolinska Institute, Stockholm, Sweden. Dr. Sergiu Catrina is a clinical endocrinologist specializing in the complications of diabetes, with a particular emphasis on impaired wound healing. His research focuses on the mechanisms that lead to these complications, especially the role of impaired hypoxic responses in diabetic patients. Sergiu adopts a translational approach, aiming to bridge the gap between laboratory research and clinical applications to better understand and treat diabetes‐related issues including diabetes wound healing. His work is widely recognized for its contributions to improving care for patients suffering from the long‐term effects of diabetes.
Professor Can Cedidi, Germany	
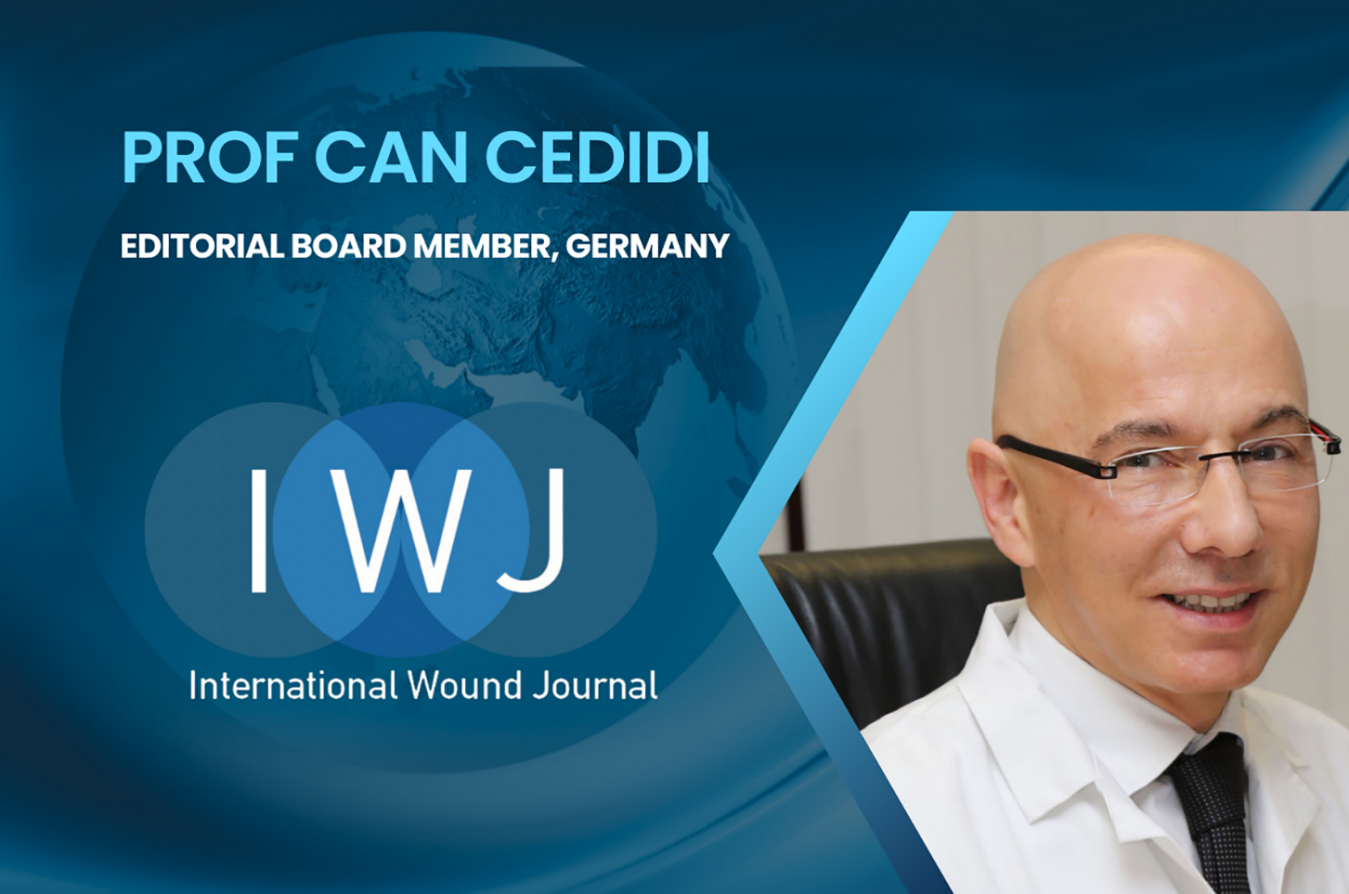	Prof. Cedidi is the chief of the Clinic for Plastic, Reconstructive, and Aesthetic Surgery at the Klinikum‐Bremen‐Mitte, which is the main maximum health care hospital centre for Bremen and the north in Germany and the academic teaching hospital of the University of Göttingen, and Witten/Herdecke where Can is teaching as a Professor. Prof. Cedidi studied medicine in one of the top medical excellence universities in Germany, which is Heidelberg University and he absolved his training in Surgery at the Hannover Medical School, at the University of Heidelberg, and the Mayo Medical School in USA. He shared his experiences in numerous internationally cited articles, book chapters, Scientific Publications, as well as numerous international presentations. He loves sharing science and experiences, that is why he is lecturing regularly in non‐stop series of online webinars for different international societies of Plastic, Reconstructive and Microsurgery with exchanging knowledge, and scientific experiences. Prof. Cedidi has established one of the best international yearly Live—Surgery Meetings worldwide, which is The SOAP—Congress, State of Art in Plastic‐Surgery. Gathering the best expert and inventor, plastic surgeons worldwide together, performing and demonstrating their tips, tricks and the last updates in Live—Surgeries, Hands on Cadaver Courses and Workshops. In the hospital Bremen Mitte, which is chaired by Prof. Cedidi and is one of the largest in Germany, the patients need a complex Plastic Surgical reconstruction after cancer, tumour or failed surgeries elsewhere. The Chief Surgeon has also made a name for himself, with excellent performance in microsurgical reconstructions, saving patients after dramatic accidents, tumours or congenital defects. Such interventions take hours and require maximum precision, that is why he succeeded to build a great professional team, always 24 h ready to save patients. Besides, Prof. Cedidi has many positions in many national and international societies of Plastic, Reconstructive and Aesthetic Surgery such as the German Society of Plastic, Reconstructive and Aethetic Surgery DGPRÄC, German Society for Surgery DGCH, International Society Aestehtic Plastic Surgery ISAPS, Interactive Plastic Surgery Network Society IPSN, Mayo Clinic Alumni Association, German Society of Burns, German Society of Hand Surgery. Eventually Prof. Cedidi is also the Chair Plastic Surgeon of the Expert Panel of the European Union.
**Professor Paul Chadwick, UK**	
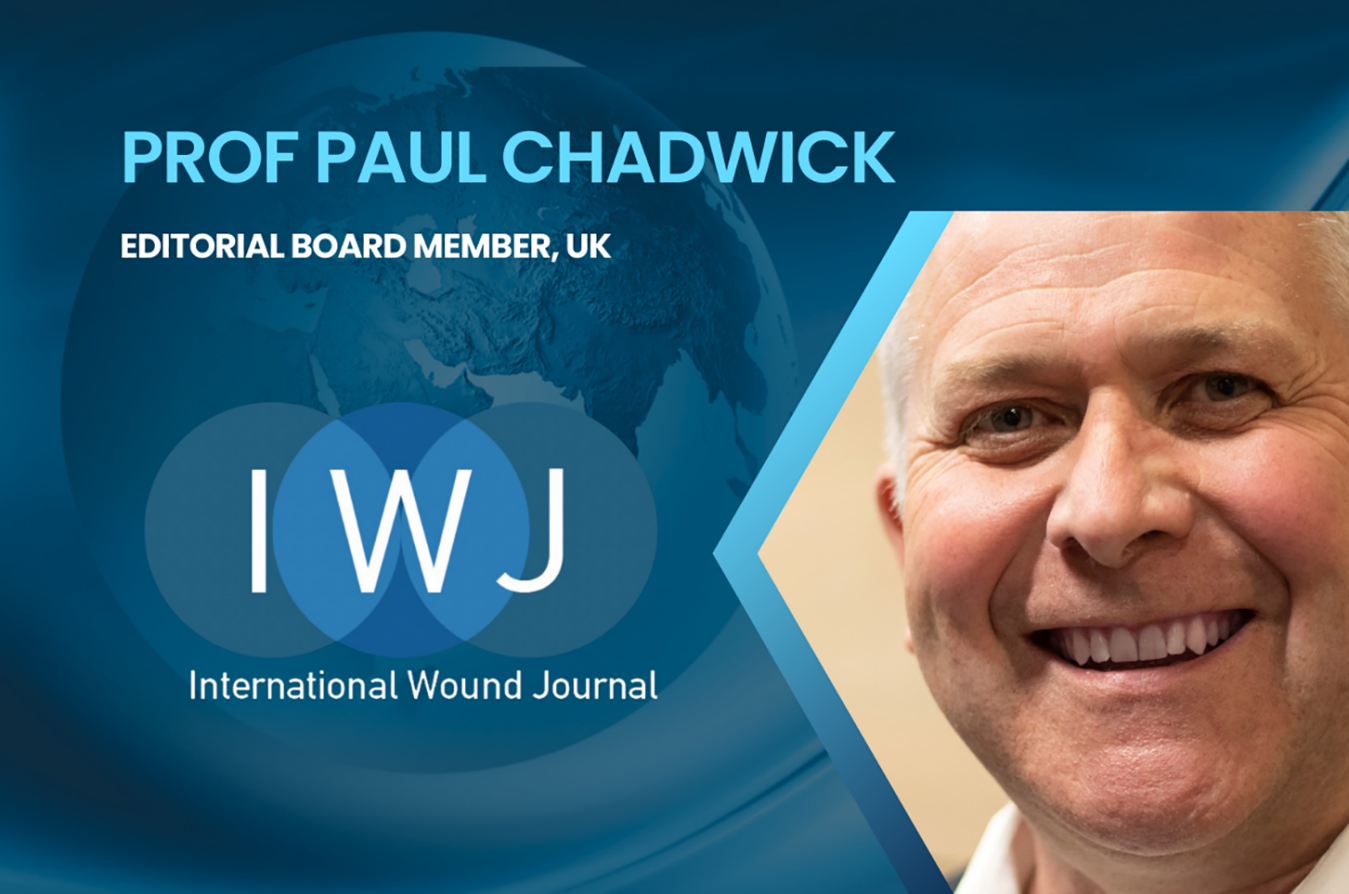	Paul qualified in 1990 and gained an MSc (by research) in 2000. He completed a PhD in 2012, looking at the social construction of neuropathic pain. He is currently the EVP of Spectral MD/AI, leading the International operation. Most recently, he was Interim Chief Executive at the Royal College of Podiatry and prior to that he was the College's National Clinical Director where he led the development and implementation of the Clinical Leadership and Education agenda of the Organizations' five‐year strategic plan. Before starting at the College, he was a consultant podiatrist; he led a team of clinical specialists and developed an integrated system wide approach to foot care. He maintains a clinical role as an honorary consultant podiatrist. He has presented nationally and internationally and published widely in many peer reviewed journals. Previously Paul was the Chair of Foot in Diabetes U.K. He is on the editorial board of a range of journals and has been appointed as associate editor of the Diabetic Foot Journal. He has a Fellowship from the Royal College of Podiatry and was awarded the meritorious award in 2014 for services to the profession. He also received a Fellowship from the Royal College of Physicians and Surgeons of Glasgow. Paul was appointed a Visiting Professor of Tissue Viability at Birmingham City University in 2018. In his time at The College, he has led many projects, including Workforce and Educational reform, HCPC annotation of podiatric surgery in England, the development of the Advanced Clinical Practice Framework in lower limb viability and the Diabetes Commissioning Tool Kit. He has also led the implementation of a new learning management system and created a suite of continuing professional development modules for the profession.
**Professor Guido Ciprandi, Italy**	
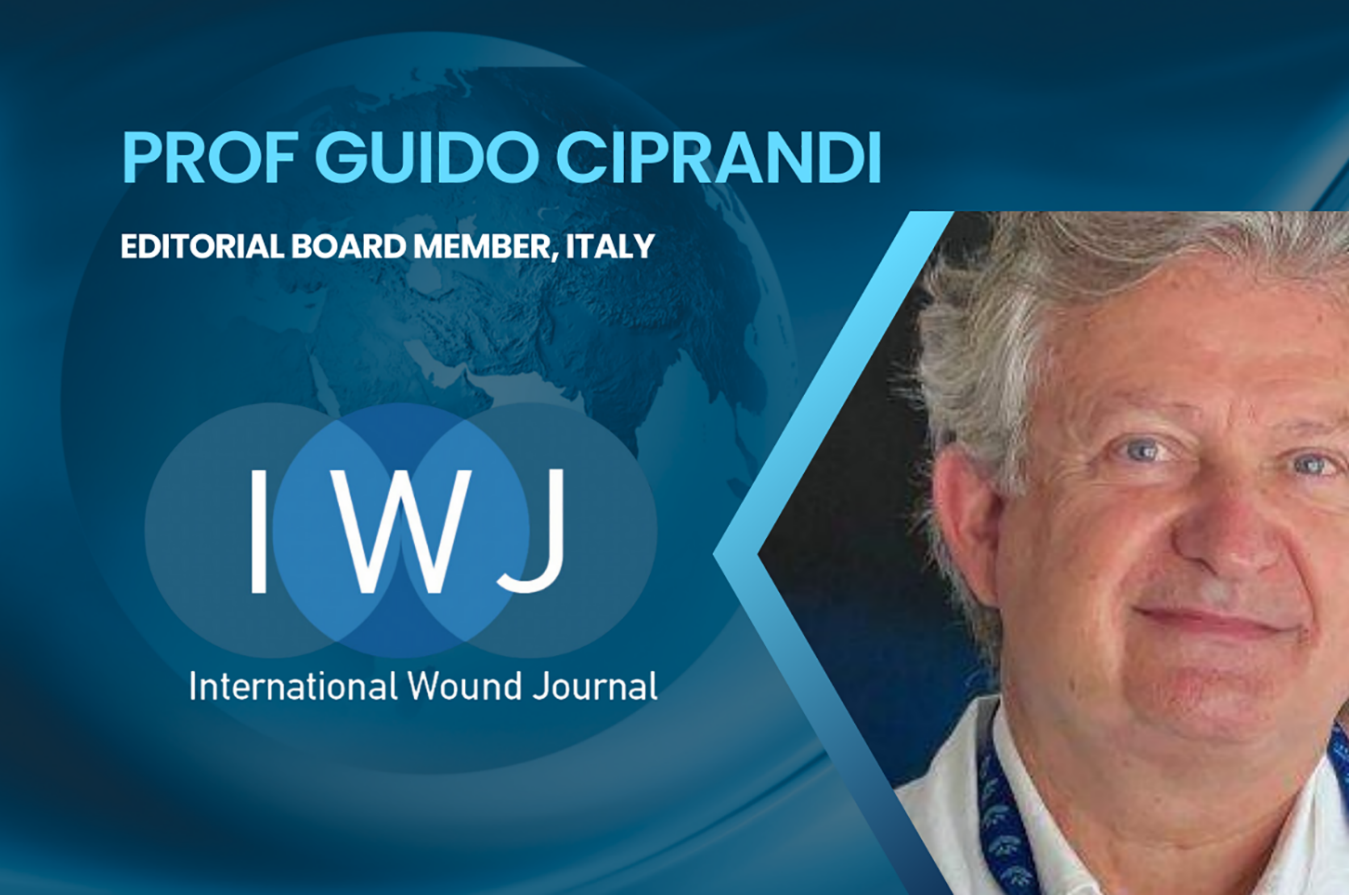	Dr. Ciprandi is a specialist in thoracic surgery and paediatric surgery. Since 2012, he is responsible for ulcers and difficult injuries at the Department of Surgery, UOC of plastic and maxillofacial surgery of the Bambino Gesù Paediatric Hospital of Rome. He also is a professor at the Universities of Rome, Florence, and Milan and author of over 300 scientific publications and books on wound care.
**Mark Collier, UK**	
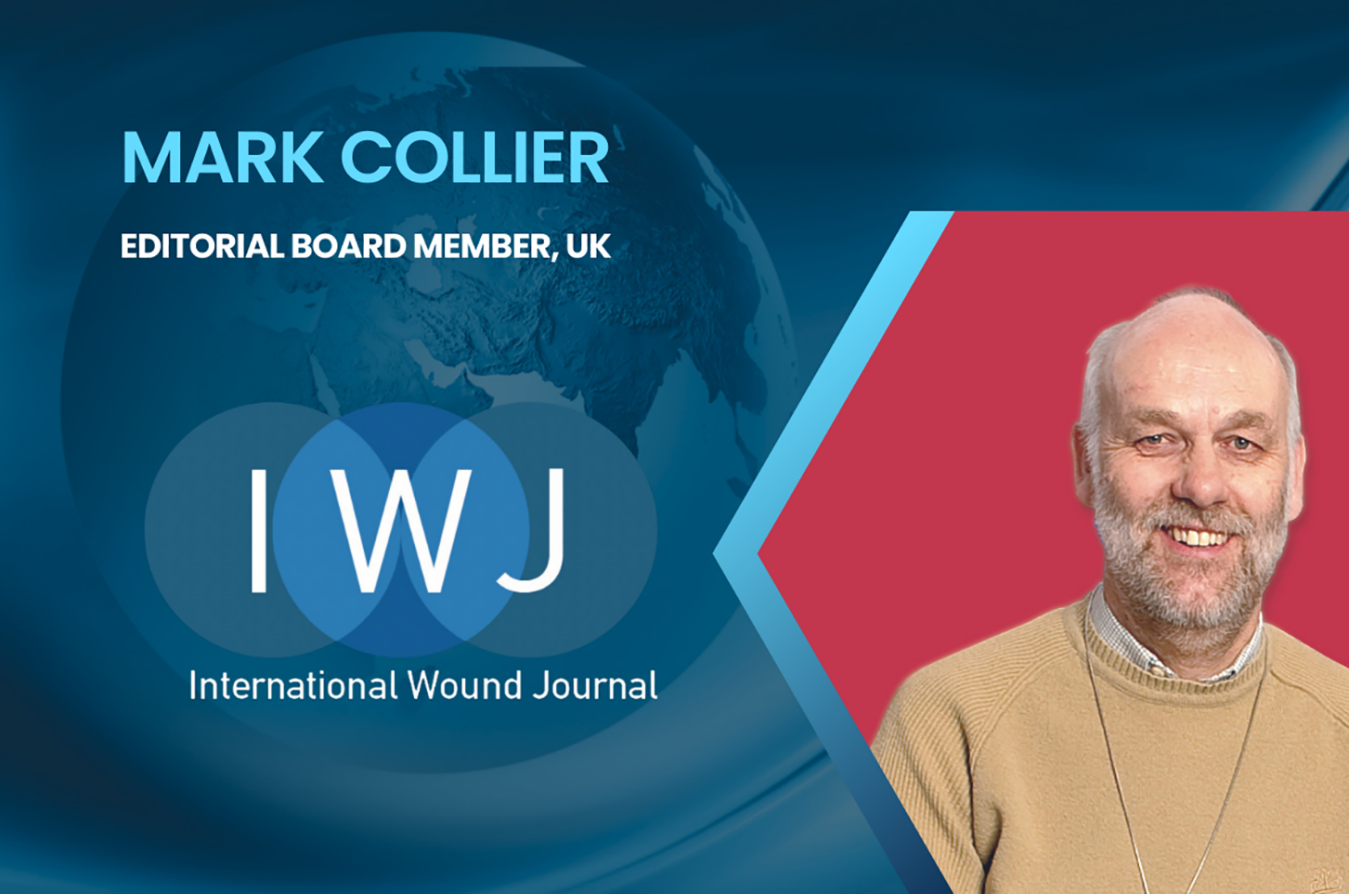	Mark qualified as a Registered General Nurse in 1982, practicing initially in the care of Trauma and Orthopaedics patients. He quickly combined his clinical experiences with his interest in Tissue Viability and as a result was a founding member of the Wound Care Society (UK) in 1988. He has also been a council member of the Tissue Viability Society (TVS), the European Pressure Ulcer Advisory Panel (EPUAP) and is currently a Council member of the European Wound Management Association (EWMA) and Chair of the Leg Ulcer Forum (England and Wales). Throughout his career, he has won many awards such as The Mary Powell Orthopaedic Nursing Award; a Smith and Nephew Foundation Doctoral Nursing Research Scholarship and most recently the British Journal of Nursing ‘Pressure Care Nurse of the Year’. A clinician first and foremost, he is currently working as a Nurse Consultant/Associate Lecturer—Tissue Viability with affiliations to Lincolnshire Health Care Services and the Universities of Lincoln and Sunderland, with his stated objective that of ‘promoting evidence‐based best practice’. He continues to review and advise on specialist patient care and is an editorial advisor for the Mark Allen Group of publications, such as the Journal of Wound care among others. He has over 300 publications (Research/Educational/Clinical) to his name and has been involved in the writing of various National and International best practice documents; NICE (National Institute for Health and Care Excellence) and EWMA Guidelines, and he remains an active NICE GDG (Guideline Development Group) member—often also being asked to review and advise on current NICE Med Tech applications. In the summer of 2023, Mark was appointed as a TV Advisor and Audit Nurse on behalf of Barchester Nursing Home Group—supported by Renray Medical (UK). Mark is often asked to present at local, national and international educational events.
**Dr. Elena Conde, Spain**	
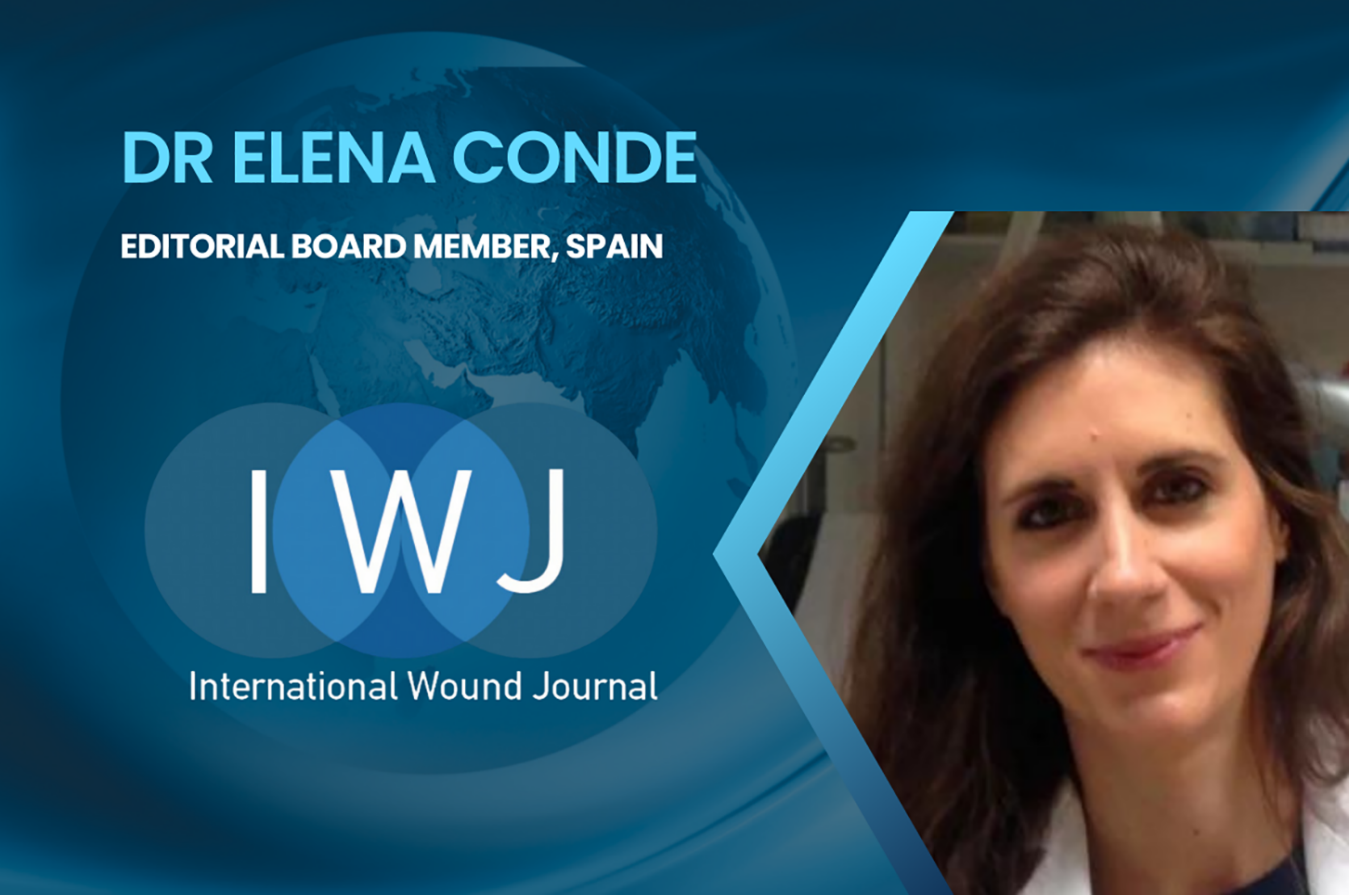	Elena has a PhD degree from University Complutense Madrid obtained cum laude and a D.U. Phlébologie, Université Pierre et Marie Curie París and a degree in Psychology UNED Madrid. She is currently a Dermatology consultant in Madrid, Hospital Universitario Infanta Leonor y Virgen de la Torre.
**Professor Sarah Curran, UK**	
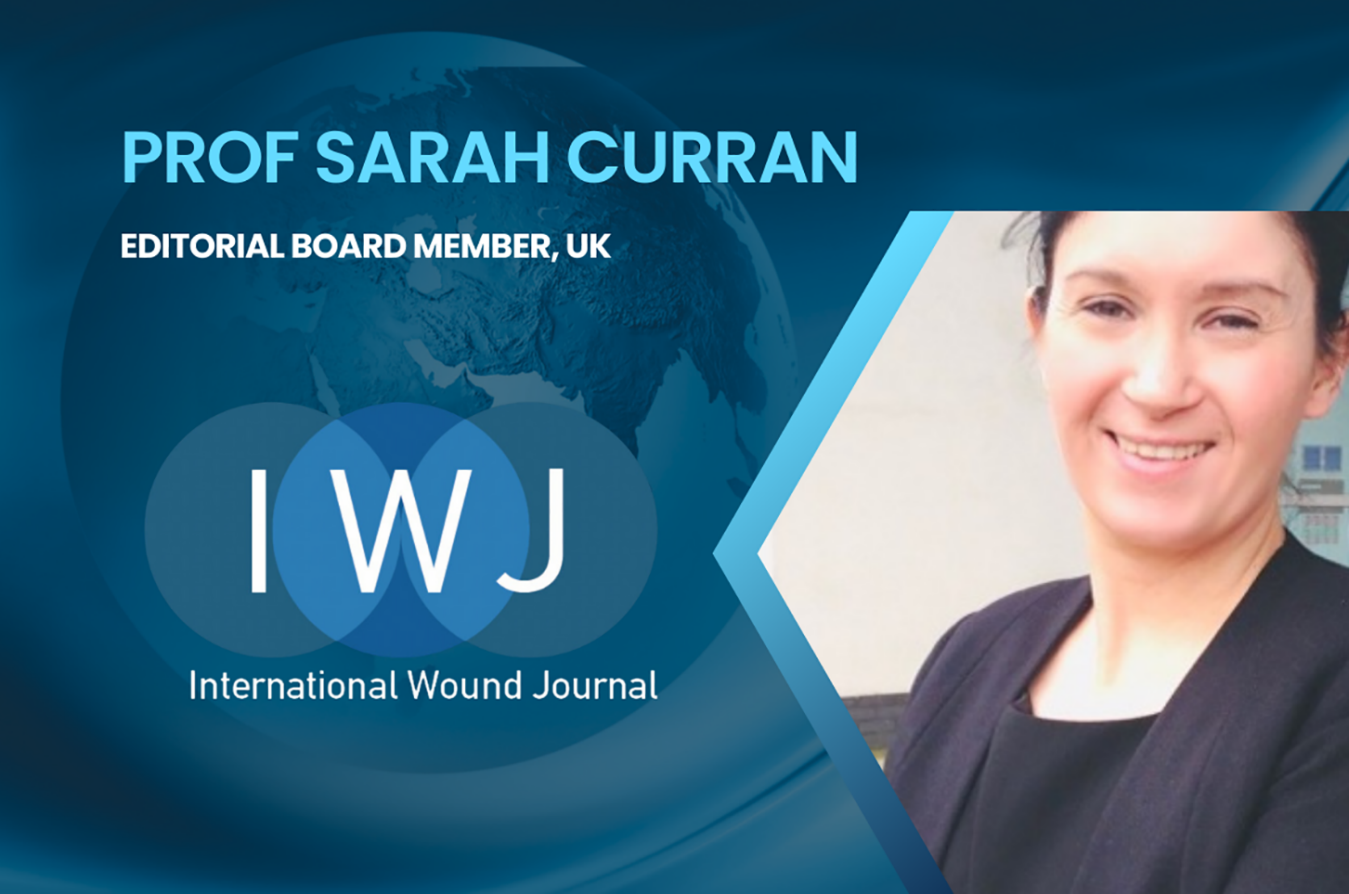	Prof. Curran teaches at undergraduate and postgraduate levels and is an Honorary Professor at the National University of Ireland, Galway. She is the Chair of the Research, Development and Innovation and Chartered Scientist Committee for the Royal College of Podiatry and has held a number of editorships, published widely and presented at national and international conferences. She holds a number of awards and fellowships, including a prestigious National Teaching Fellowship awarded in 2016. Prof. Curran has undertaken a number of pedagogic research projects but has a particular interest in musculoskeletal pathologies of the lower limb, gait function and posture.
**Dr. Rosemarie Derwin, Ireland**	
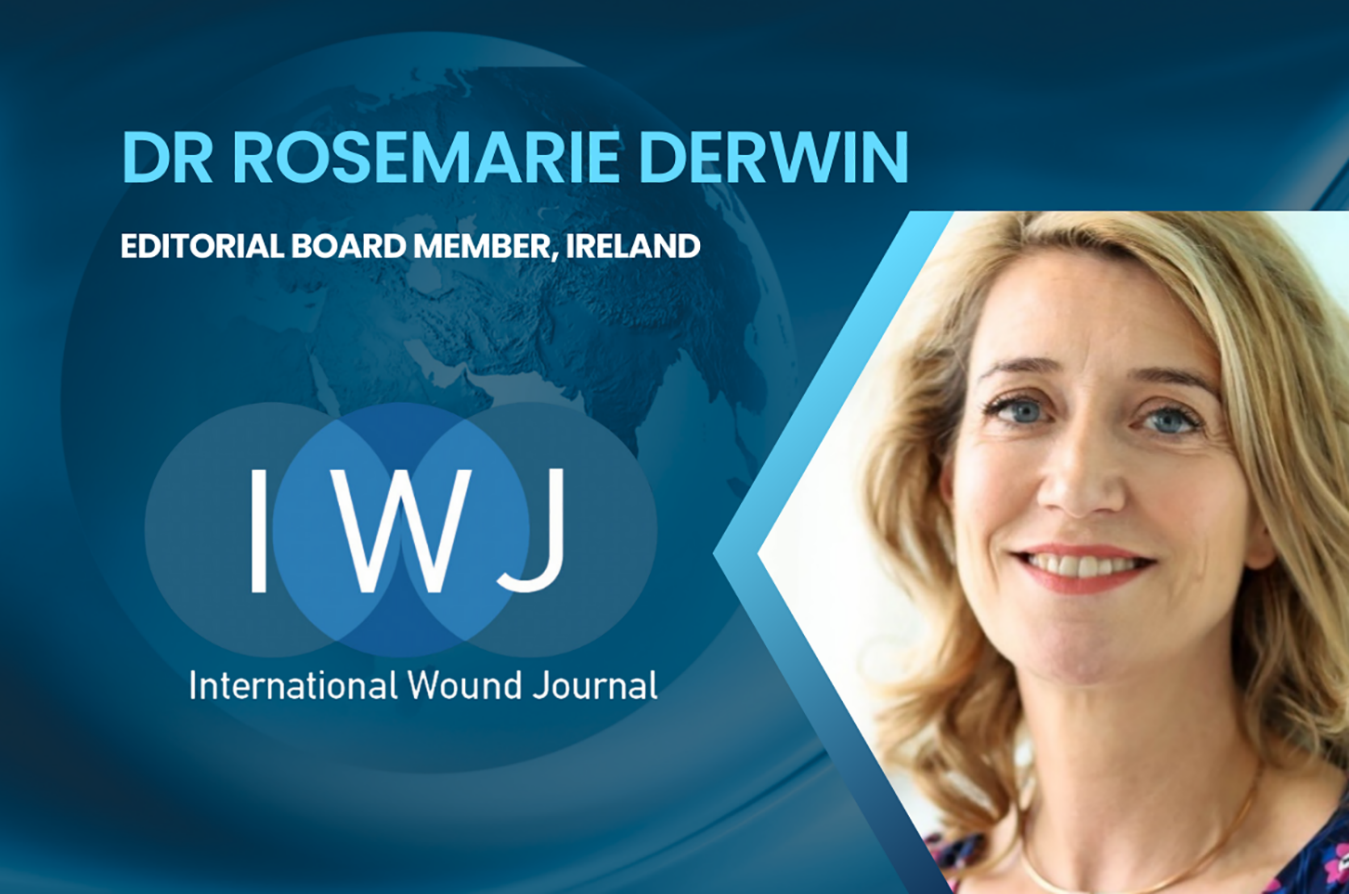	Dr. Rosemarie Derwin is lecturer and programme director in the School of Nursing and Midwifery in the Royal College of Surgeons in Dublin. She is also a lead researcher in the Skin Wounds and Trauma (SwaT) Research Centre. After the completion of her general nurse training in the Netherlands in 1995, she returned to work in Ireland working for a number of years in Beaumont Hospital and the Mater Misericordiae University Hospital. She has a background in head and neck cancer, haematological nursing, care of the older person and tissue viability. She worked in a range of roles in clinical practice, management and education. Her research has focussed on wound healing. She is currently a trustee on the European Pressure Ulcer Advisory Panel (EPUAP).
**Professor Valentina Dini, Italy**	
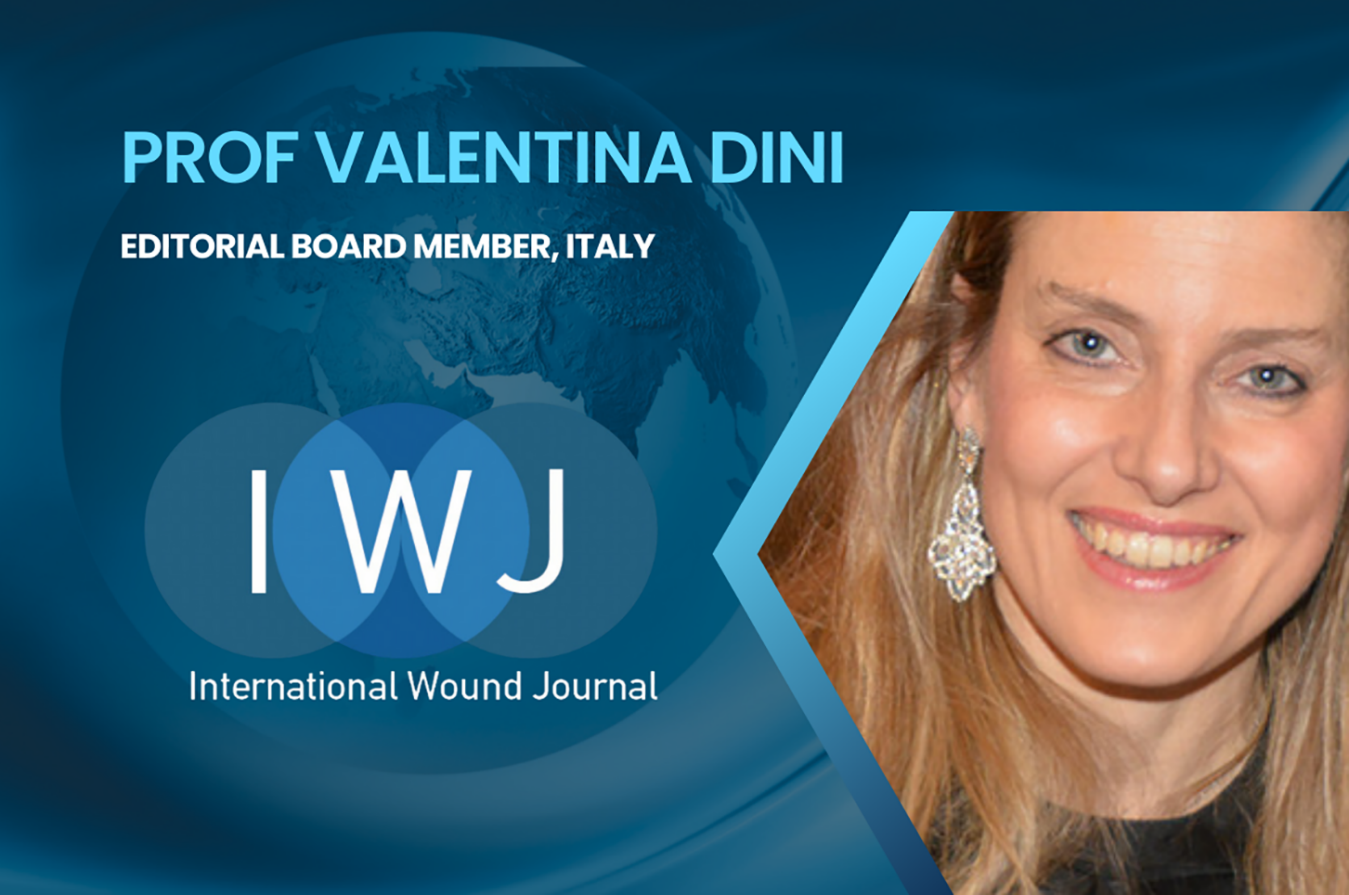	Valentina Dini received her medical degree (2000) and her residency in Dermatology (2005) at the University of Pisa, where she obtained her doctorate in Immunology and Technology of Transplantation in 2009, also completing a fellowship in wound healing (2006) at the University of Miami. For 3 years, she was post‐doctoral fellow at the Dept. of Dermatology and Venereology, University of Pisa. Valentina is a researcher at the University of Pisa pursuing her research work at the Dept. of Dermatology and Venereology. Her research interests are tissue repair of chronic wounds and psoriasis. She has designed and conducted clinical trials to evaluate novel therapies for psoriasis. The major part of her activities was focused on setting up her laboratory of non‐invasive diagnostic techniques for skin assessment. She has ongoing collaborations with the department of rheumatology and the department of chemistry in a variety of preclinical and clinical studies. Efforts of her laboratory continue to be focused on all aspects of translational research in wound healing and psoriasis. Dr. Dini's scientific accomplishments resulted in numerous publications in prominent scientific journals. She continues to dedicate her efforts to research, education through supervising clinical care of our residents and providing clinical and dermatopathology teaching cases as well as frequently participating in research projects and teaching in national and international conferences. Dr. Dini joined as a co‐investigator in an international research group, which was the recipient of European Union 7th FrameWork Programme grant. She is a member of a Tuscan committee for the guidelines development of psoriatic patients management and Hidradenitis Suppurativa management. She is president of the International School of Tissue Repair (SIRTES). She is the author of several papers, lectures to international conferences and chapters in international books. She is in the editorial board of Acta Vulnologica and Chronic wound care and Research journals.
**Professor Michael Edmonds, UK**	
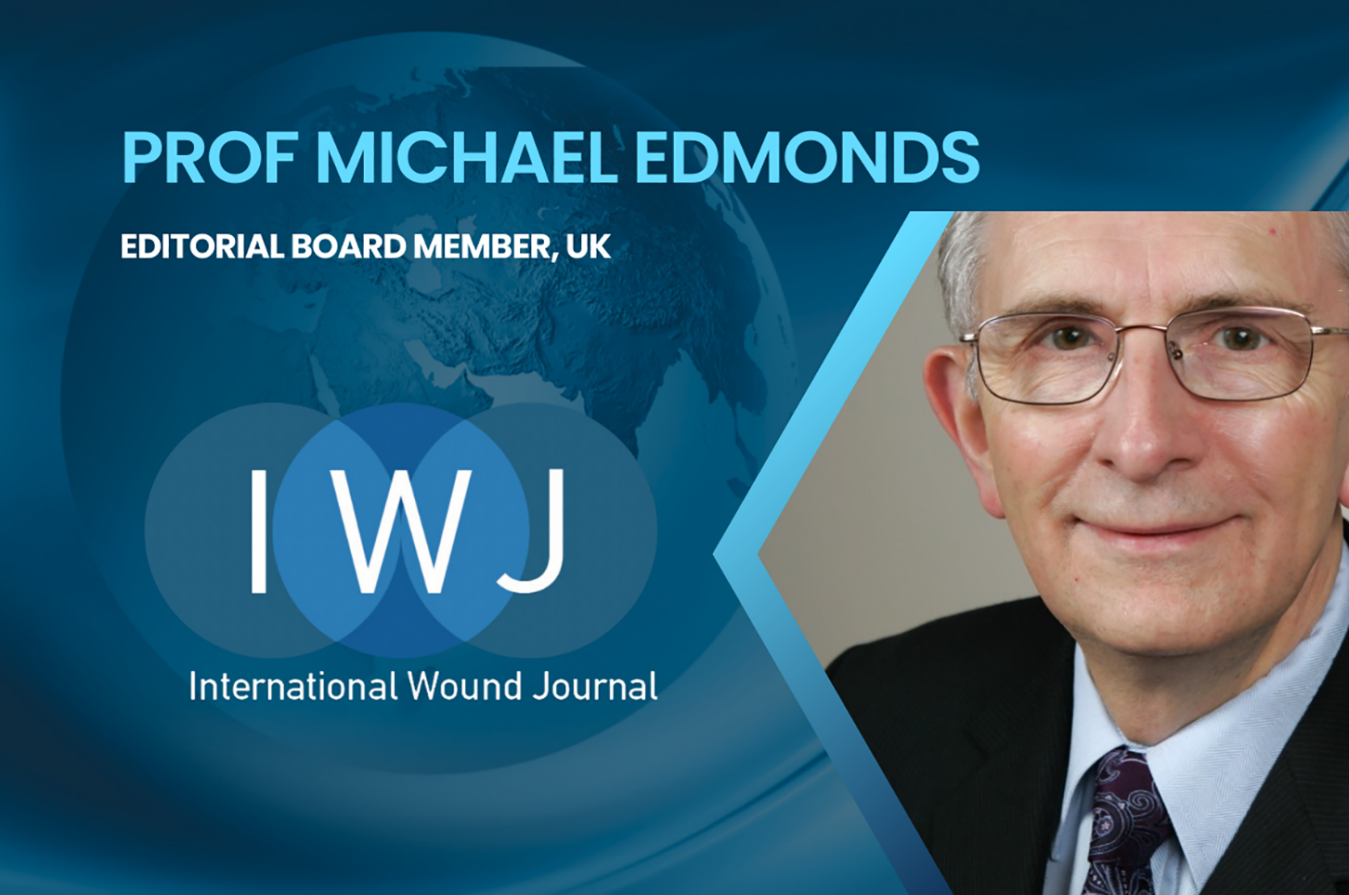	Michael Edmonds is Consultant Physician at King's College Hospital, London, with special interest in the care of people with diabetes complications. His work has had three main aims: first, to develop the best possible care for the person living with diabetes and foot problems; second, to research the causes of and devise new treatments for diabetic foot problems; and third, to teach health care professionals about the best care of the person with diabetes foot problems. He developed a novel model of diabetic foot care, the multidisciplinary Diabetic Foot Clinic, in 1981, to provide early, aggressive treatment of the various factors that contribute to the rapid progression of diabetic foot problems. The clinic at King's College Hospital brought about a 50% reduction in major amputations in people with diabetes. This underpinned the St Vincent declaration, which, in 1989, set a target, in Europe, of reducing major amputations by 50% in people with diabetes. He was Chairman of the Diabetic Foot Study Group (DFSG) of the European Association of Diabetes from 2001 to 2005. He won the Karel Bakker award at the 6th International Symposium on the Diabetic Foot in 2011, the DFSG Lifetime Achievement Award in 2013 and the Edward James Olmos Award for Amputation Prevention in 2014. He gave the Arnold Bloom Lecture ‘The benefits of working together in diabetic foot care for the vulnerable patient’ to Diabetes UK Annual Professional Conference in 2014. He has recently jointly formed the ZAP Amputations group (Zero All Preventable) Amputations of FDUK (Foot in Diabetes UK). He is Chairman of this multidisciplinary group, which sets out to reduce the number of amputations in people with diabetes. Michael Edmonds believes that amputations are not inevitable, and many can be prevented.
**Professor Valerie Edwards‐Jones, UK**	
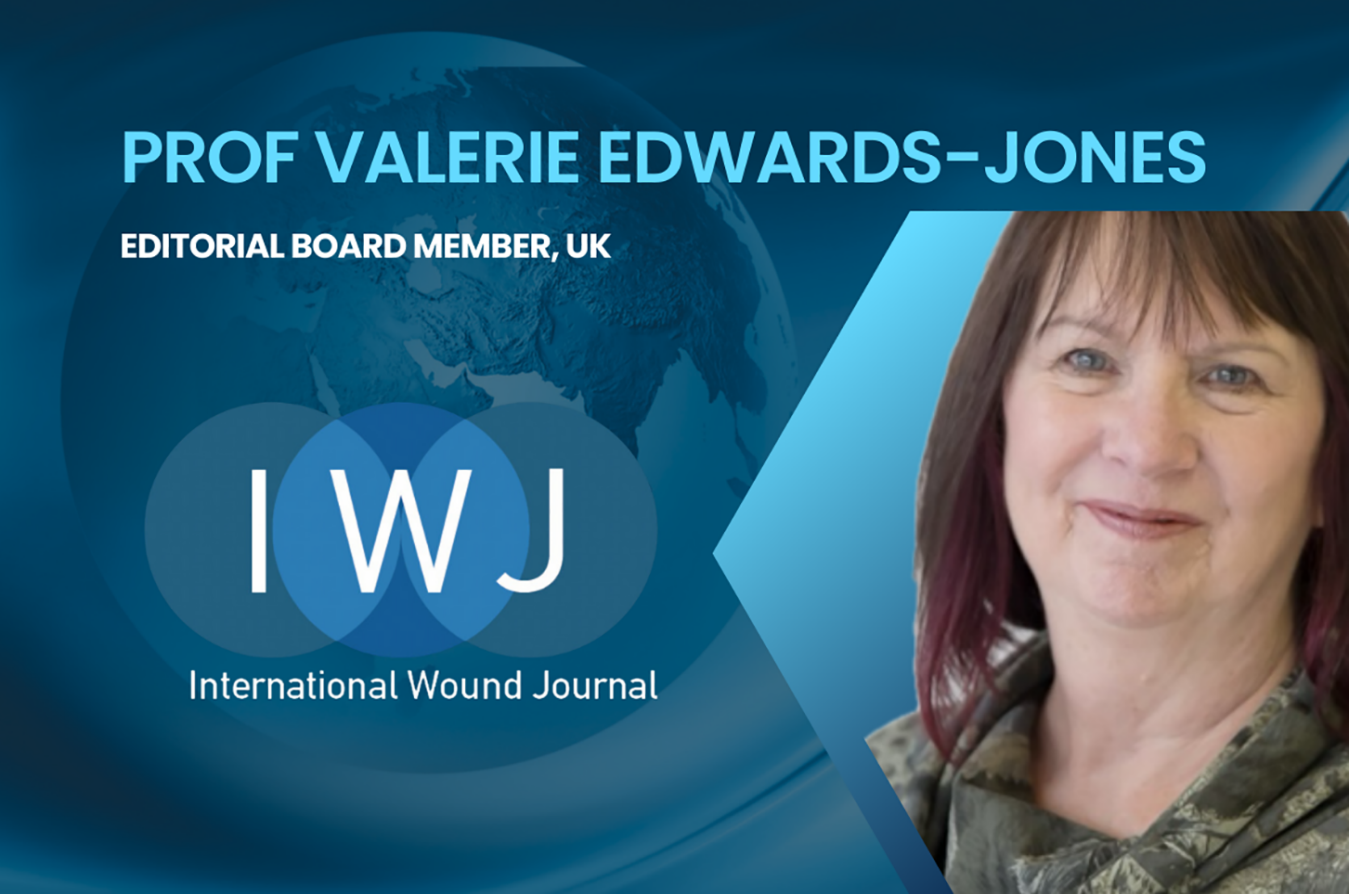	Valerie has over 40 years of experience in the field of medical microbiology, with particular interest in antimicrobial resistance, diagnostic techniques and wound microbiology. Her PhD was ‘toxic shock syndrome in burned patients’ and here she developed her experience in the physical interaction of microorganisms with antiseptics, especially silver. Valerie has published widely throughout her career and frequently attends expert panels/advisory boards, recently contributing to the fields of infection control, antimicrobial stewardship and diagnostics of wound infection.
**Dr. Leigh Fleming, UK**	
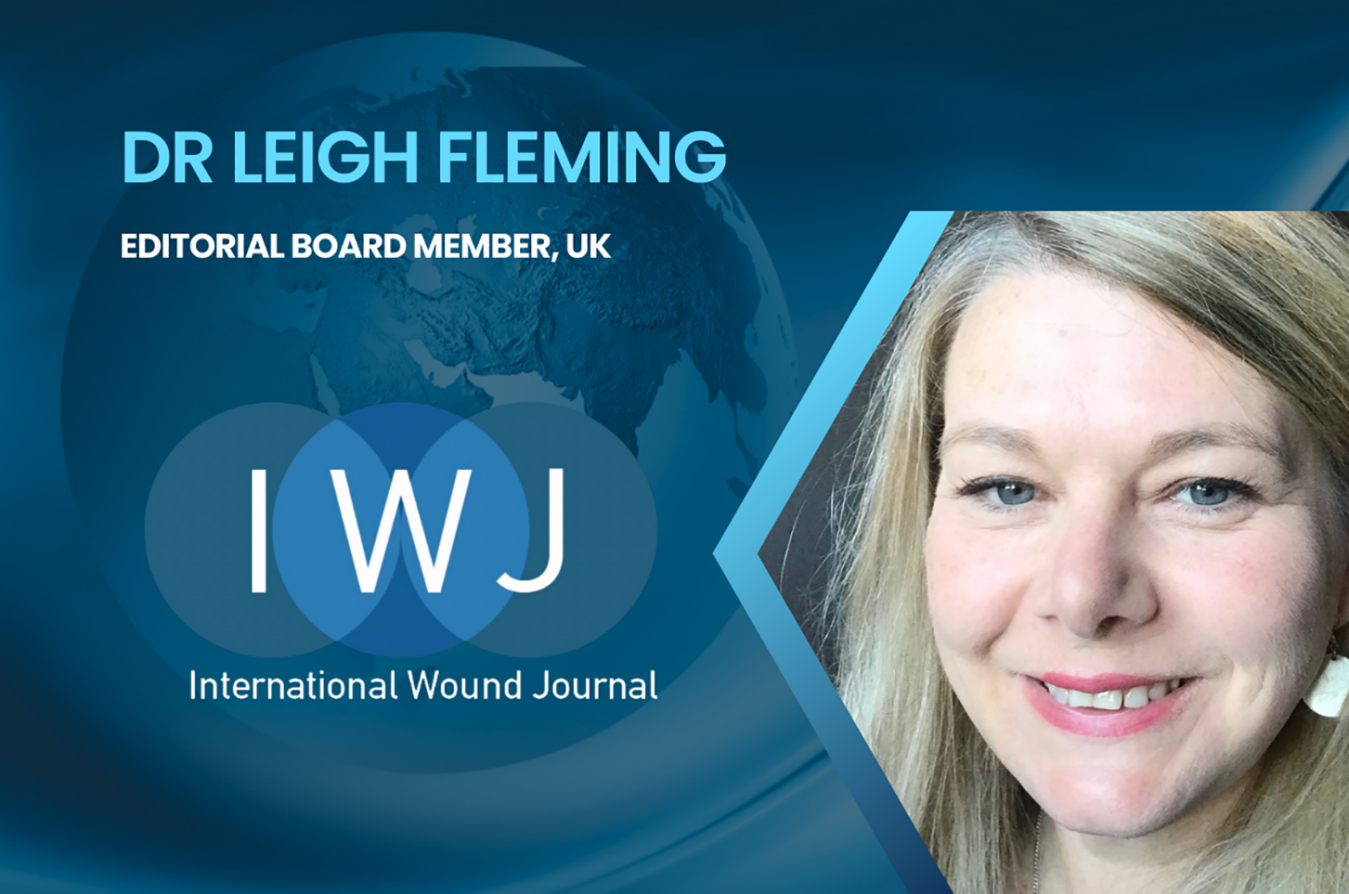	Leigh is currently the Acting Head of Department for Engineering in the School of Computing and Engineering. After graduating with a BEng(hons) in Engineering Design: Mechanical from the University, she continued her studies completing a PhD in ‘The Use of 3D Analysis Techniques to Investigate the Wear of Femoral Stems in Total Hip Replacement’ before moving into industry, working for DePuy International in their research and test department. Leigh's current research interests include application of metrology to novel surface characterization, in particular with skin interfaces, in particular for wound care, pressure area care and pressure ulcer prevention and also the development and evaluation of assistive technologies for improved health‐related quality of life. She is also active in developing research around the integration of predictive health maintenance systems in SMART homes for improved health outcomes.
**Dr. Anika Fourie, Belgium**	
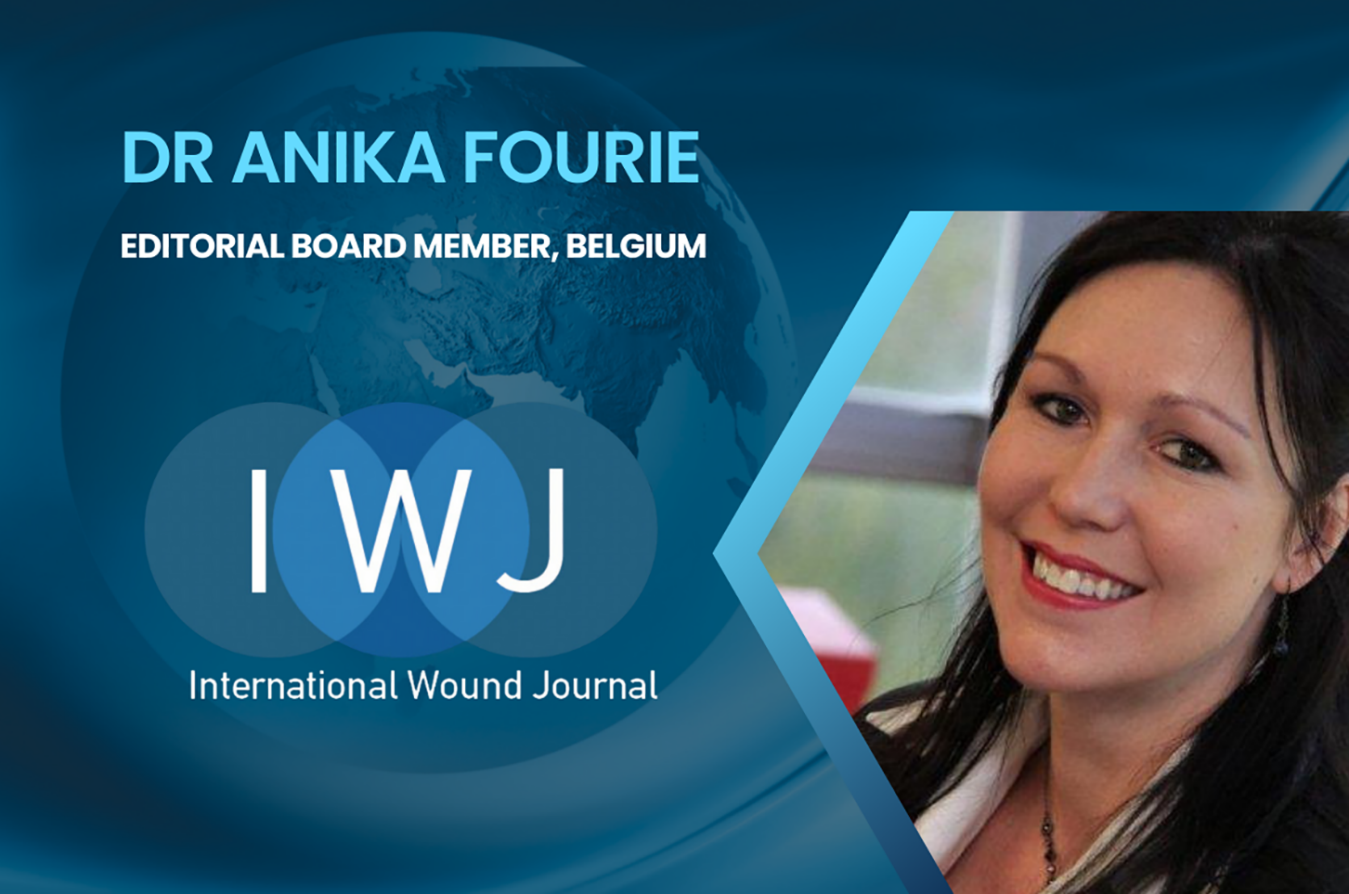	Anika started her doctoral studies in 2020 at the University Centre for Nursing and Midwifery at Ghent University, Belgium, and is part of the Skin Integrity Research Group. She has a critical care nursing background and is a qualified wound care specialist who managed a wound care clinic in South Africa until 2008. For 11 years, she worked in the industry sector as the regional clinical manager, responsible for education, key opinion leader development, and quality improvement projects across Europe, Middle East and Africa. She obtained her master's degree in clinical Skin Integrity a Wound Management in 2018 from Hertfordshire University, UK. She is a peer reviewer for Advances in Skin & Wound Care, Journal of Wound Care, and Journal of Wound Management, and was recently acclaimed as Regional Director for Europe for ISTAP (International Skin Tear Advisory Panel). The title of her PhD thesis is ‘The prevention of skin/tissue damage in hospitalized patients: the journey from gap analysis to training needs analysis and clinical trials’, with a specific focus on the prone ventilated patient in critical care.
**Professor Frances Game, UK**	
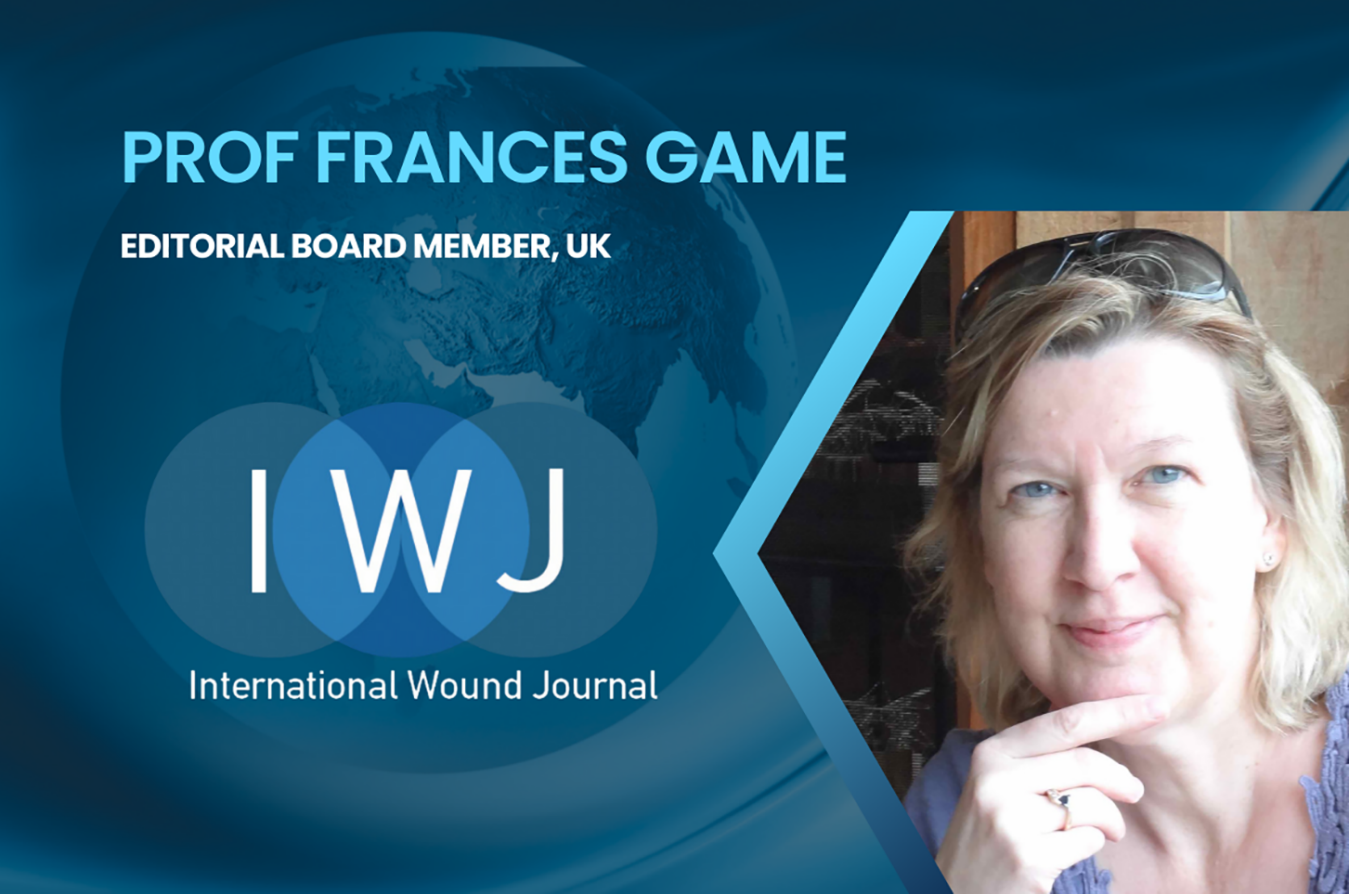	Professor Fran Game has been a consultant Diabetologist at the University Hospitals of Derby and Burton NHS Foundation Trust since 2011. She is the Clinical Director of R&D and the Derby Clinical Trials Support Unit, and Honorary Professor at the University of Nottingham. Her main clinical and research interest is the Diabetic Foot, leading to a number of multi‐centre/multinational trials in this field. She chaired both the Classification and Wound Healing subgroups of the International Working Group of the Diabetic Foot for the 2023 guidelines, has been a member of the Editorial Board of this organization for the last 4 years and is the incoming Chair from January 2024. She is the current Clinical Lead for the National Diabetes Foot Audit of England and Wales and has been one of the NHS England Co‐Clinical Regional Directors for Diabetes (Midlands), since May 2018. She was awarded the Edward James Olmos Award for Amputation Prevention by the American Limb Preservation Society in 2021.
**Santiago Gómez, Spain**	
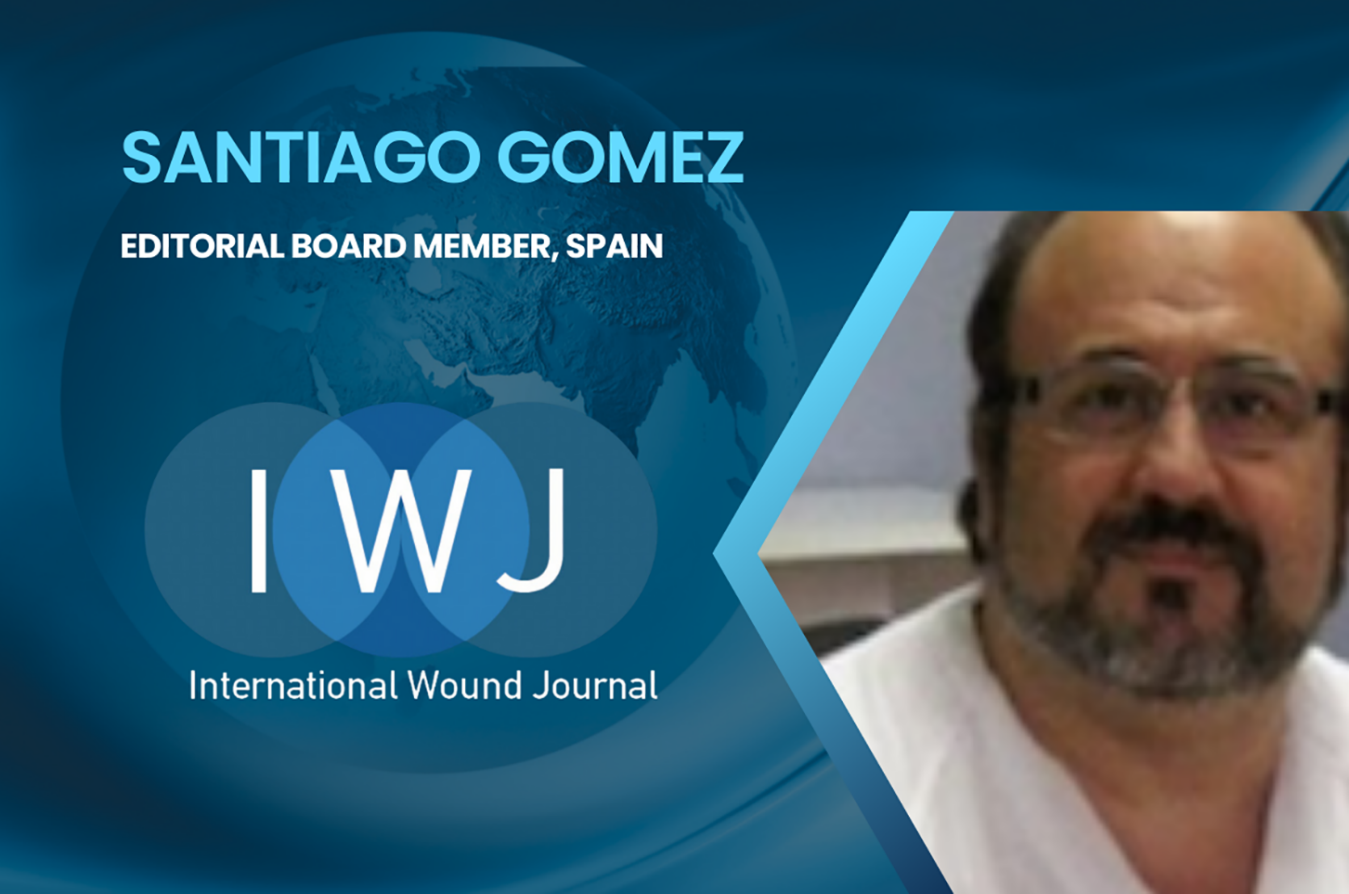	Santiago studied nursing and is an Official Master in deterioration of Skin Integrity Ulcers and Wounds. As an expert in diabetic foot and emergency management, he is responsible for training others through a Sergas programme. He is also the Sergas Reference for Complex Wounds (Galician Health Service) and an undergraduate clinical tutor. He is an advisory committee member of Ulceras.net. He is a former member of the Scientific Committee and Wounds Group of the Spanish Association of Vascular Nursing and Wounds. He is also a former member of the steering committee of the Galician Wound Society. Santiago is an honorary founding member and former advisor of the Latin American multidisciplinary Confederation of Wounds and Ostomies (COMLHEI) and honorary member of GADICIMe (Andalusian Group for Development and Research in Minor Surgery). Santiago acts as an external reviewer for ISSSTE LPP Guide (Mexico). He has authored several guidelines and book chapters, including Guide to soft tissue infections (SAS) and diabetic foot chapter coordinator (D. AEEVH Consensus 2017). He is the author of the Roviralta Technique.
**Professor Beáta Grešš Halász, Slovakia**	
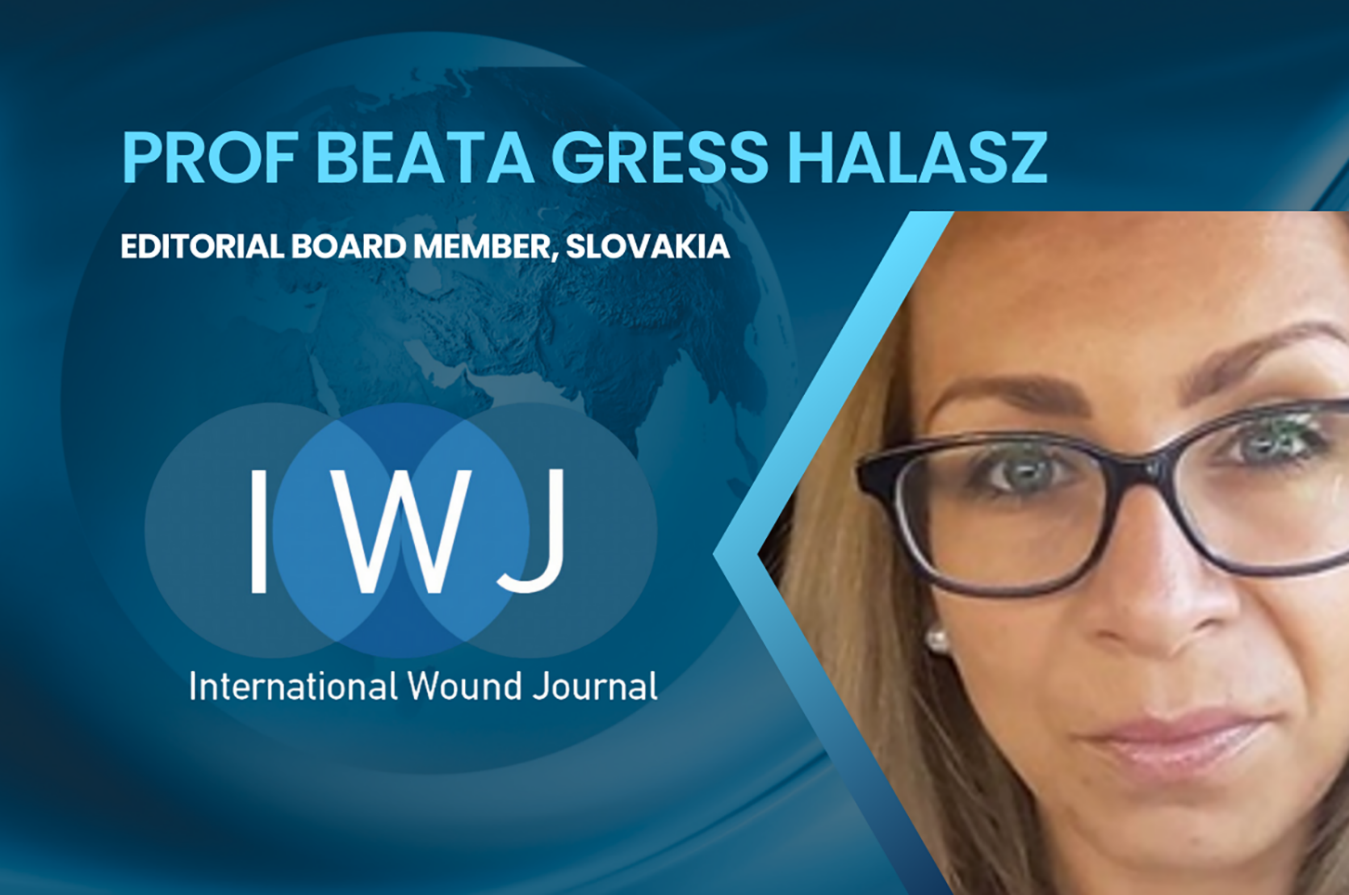	Beata works at the University of Prešov, Faculty of Health Sciences, Department of Nursing, Prešov, Slovakia, and is a fellow assistant and chair of the SSOOR (Slovak Wound Care Society). An experienced nurse in the care of chronic wounds with a specific focus of pressure ulcers. Her interests are practice and education in the field of wound care, advanced nursing roles in wound management, wound management strategies and regulations in Slovakia, and national standard procedures in wound management. She currently lectures, researches and publishes in the field of nursing care for patients with chronic wounds. She is a member of the workgroup for standard diagnostic and therapeutic procedures for wound management.
**Sylvie Hampton, UK**	
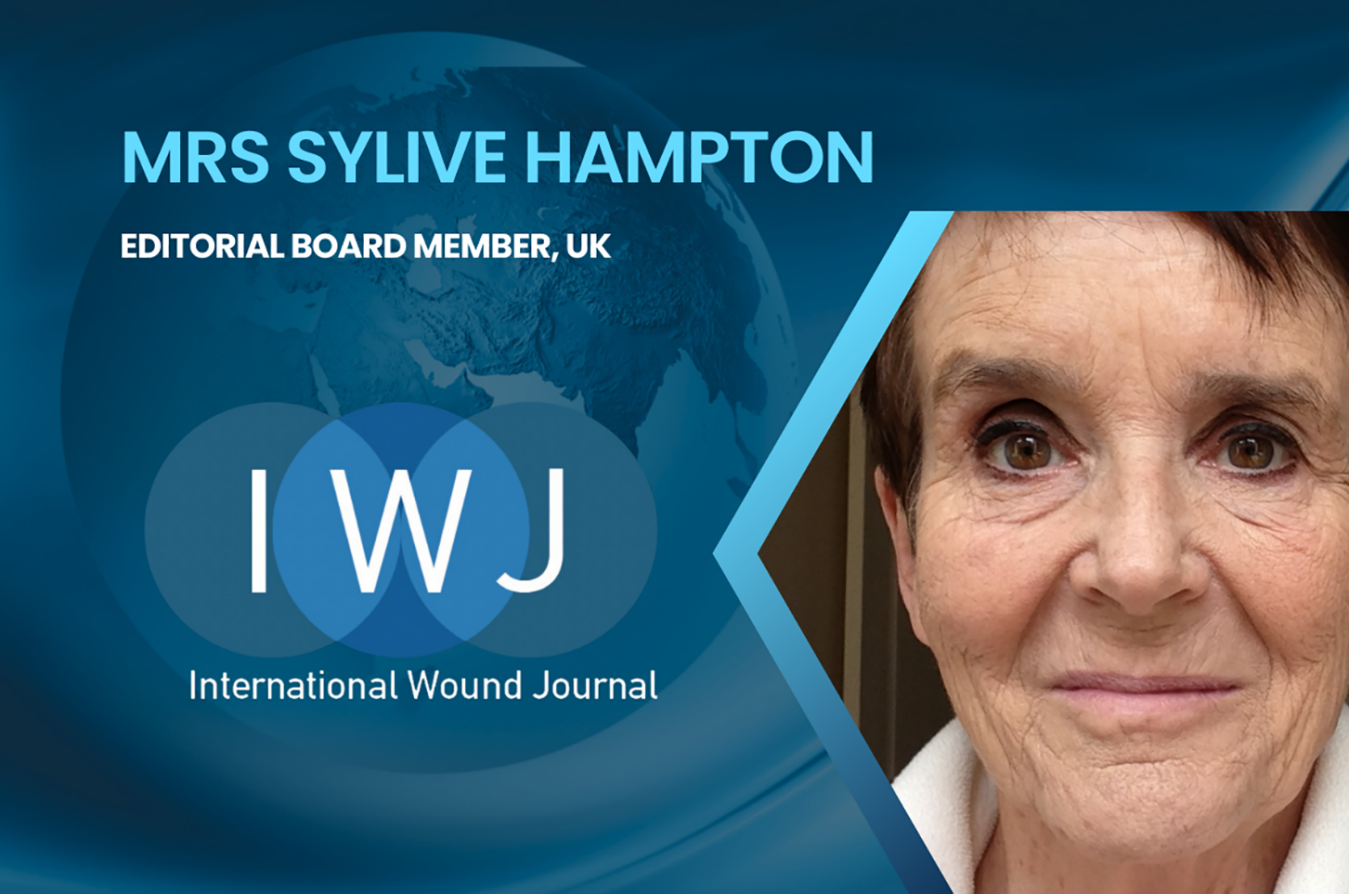	Independent TVNC Sylvie Hampton has 25 years of experience in wound care: 4 years as Enthusiast, 5 years as Specialist Nurse in a hospital and 16 years as Independent Consultant running a private wound healing centre commissioned by the NHS to care for chronic wounds. She has written 400 articles and two books on wound care. She was a Trainer and advising nursing homes, District Nursing Teams, Practice Nurses and Hospital Nurses and also acting as an Expert witness. She has been working as a Specialist Adviser for CQC. She undertakes research in wound care/pressure ulcer prevention. She is a Consultant for Lindsay Leg Club Foundation and Marie Curie Hospice movement. Educator, organizing study days and conferences.
**Britt Hansen, Denmark**	
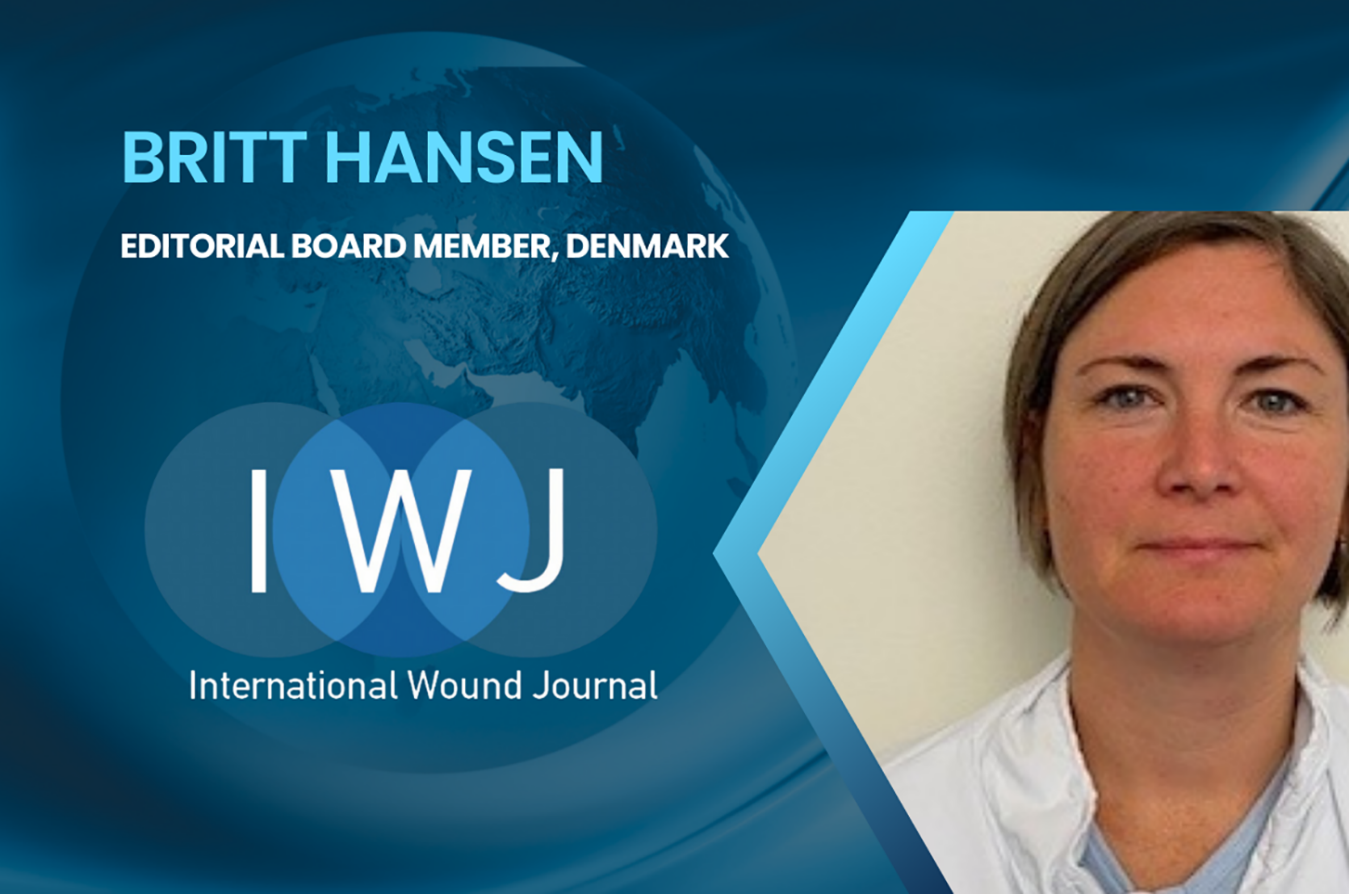	Britt is a Registered Nurse (2005), with a Masters in Clinical Science, MSs in nursing from Aarhus University (2019). She works at the ‘University Center of Wound Healing’ in the Department of Plastic Surgery since 2006 (a centre with multidisciplinary teams containing orthopaedic surgery, plastic surgery, endocrinology and dermatology). She also works as a Wound and Pressure Ulcer nurse (2019–present): Organizing and coordinating the work preventing and treating pressure ulcers at the hospital (850 beds and 38 departments). Britt is heavily involved in translating the EPUAP Quick Reference Guideline into Danish (2017); delivering the Masterclass of EPUAP in Ghent (2017); and an EPUAP trustee (2018–2024), as part of the Educational Committee. She was a part of the local organizing committee within the Focus Meeting (virtual) in Sønderborg, Denmark 2021. Britt has expertise with education of health professionals in all aspects of preventing and treating wounds, especially pressure ulcers; clinical expertise in wound treatment, and especially pressure ulcers in all categories; and the implementation of new knowledge and methods in clinical practice. She has clinical experience with patients with spinal cord injury. She prepares instructions and small guidelines about mattress equipment to prevent pressure ulcers, use of bandages, and cleaning of mattress to the staff at the hospital. She works in the education of wound care nurses and as evaluator during their examination.
**Dr. Michel Hermans, Netherlands**	
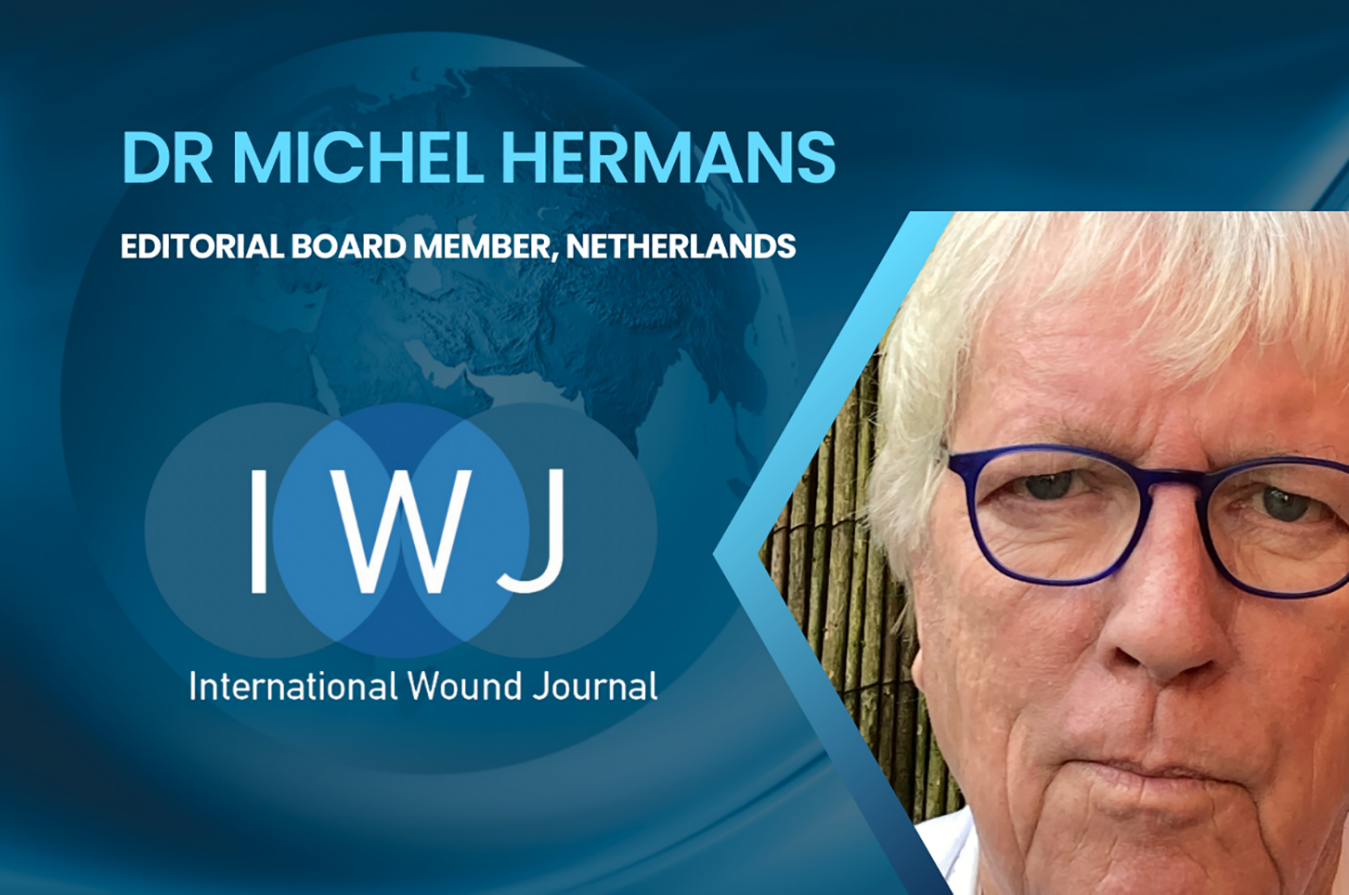	Michel H.E. Hermans MD did his medical, surgical and burn care training in the Netherlands. After years in clinical practice, he joined the wound care industry where he played a number of global leading roles in medical and clinical departments. In 2004, he started his own consulting company, which focusses on the clinical aspects of the surgical and medical device industry, with special interest in wound and trauma (burn) care. He has been, and still is a medical consultant to a number of companies. He returned back to his native country in 2021, after having lived in the United States for 30 years, to continue his consulting practice from the Netherlands. He has given more than 400 lectures worldwide, has published more than 200 articles, book chapters and posters, edited a number of journal supplements, and is member of the editorial board of a number of wound and burn journals.
**Samantha Holloway, UK**	
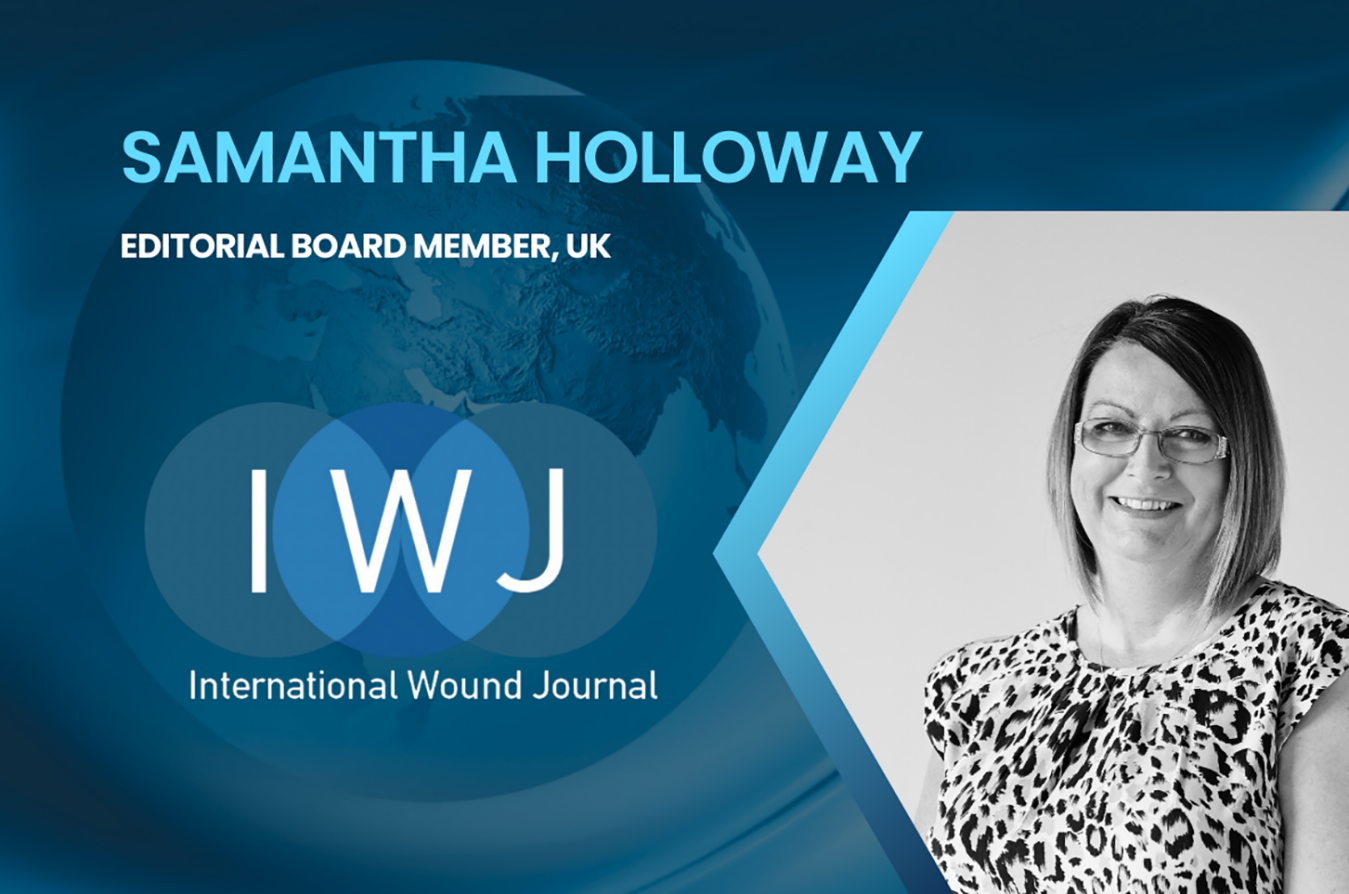	Samantha is a Reader in the Centre for Medical Education at the School of Medicine, Cardiff University Wales UK. Samantha is a Registered Nurse who has a Postgraduate Certificate in Education, a Masters in Social Science Research and is a Senior Fellow of the Higher Education Academy. Samantha's main role in the Centre is as the Programme Director for the Masters in Wound Healing and Tissue Repair which she has been responsible for since 2008. During that time, she has supervised 83 students through to successful completion of the Masters programme. Samantha is an author/co‐author of over 90 publications and has presented at national and international wound care conferences. She is the current President of the International Skin Tear Advisory Panel (2022–2024) and currently holds the position of the Chair of the Teacher Network for the European Wound Management Association (EWMA), having been the Chair of the Education Committee for the same association between 2019 and 2023. Samantha was integral to the development of a series of published EWMA wound management curricula for physicians and nurses between 2017 and 2020. She is a peer reviewer for several journals including BMJ Open, Journal of Wound Care, British Journal of Community Nursing, Nursing Standard, Journal of Wound Management and the International Wound Journal; she is also the Academic Editor for the Wounds UK journal. Samantha holds several external positions including being a member of the Executive Team of the Welsh Wound Innovation Centre, External Examiner for the University of Huddersfield and a member of the Education and Workforce workstream of the National Wound Care Strategy Programme (England). Her areas of interest include wound care education at undergraduate and postgraduate level, qualitative research and quality improvement in wound care as well as research into the prevention, assessment and management of skin tears.
**Professor Ingebjørg Irgens, Norway**	
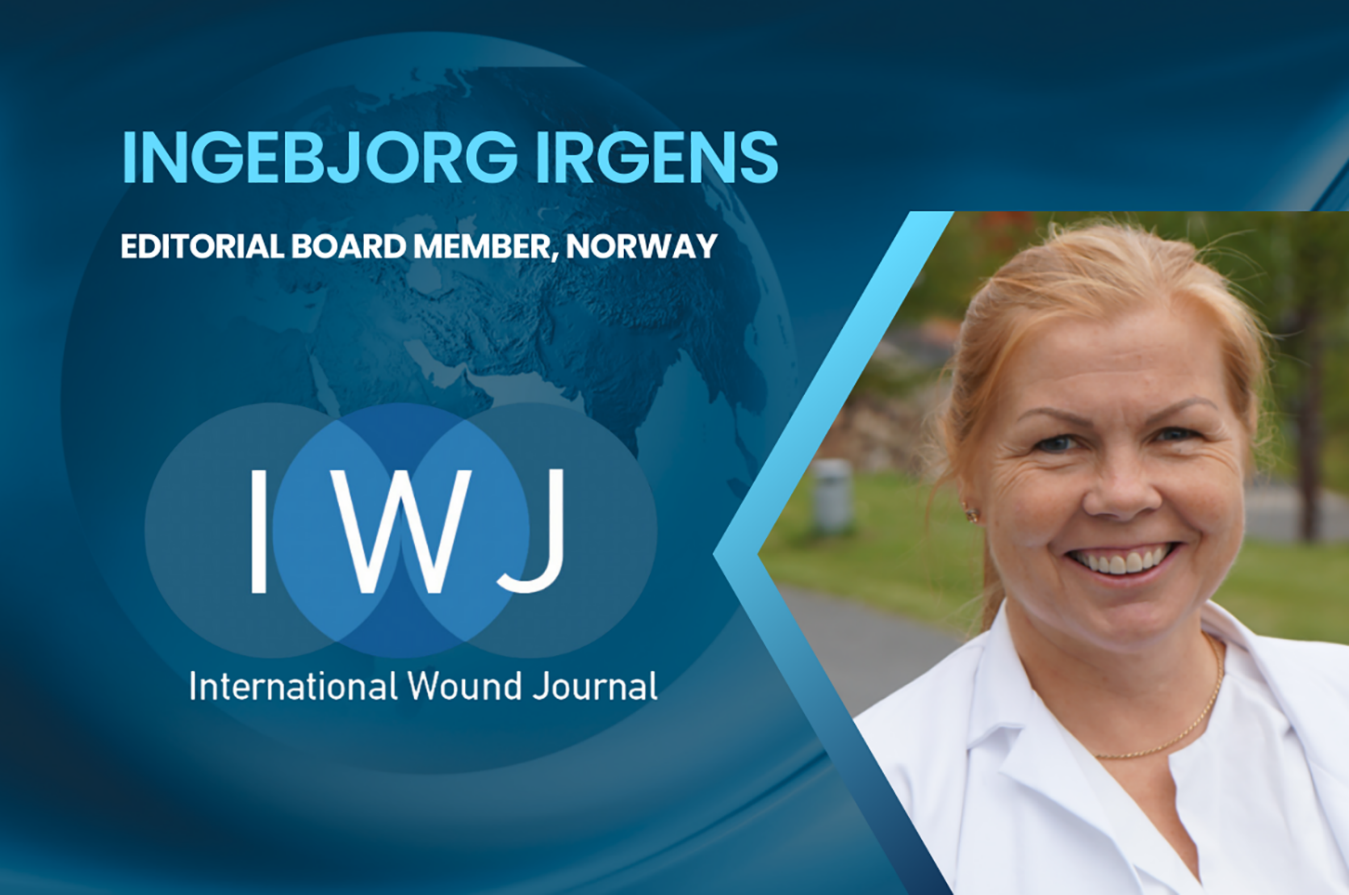	Dr. Irgens is the Manager of the Research laboratories and Chief physician at the Research Department, Sunnaas Rehabilitation Hospital. She is a specialized physician in Physical and Rehabilitation Medicine (PRM). Her journey with pressure injuries began in 2004 while working with individuals with spinal cord injuries. Witnessing the challenges these patients faced, such as recurring skin damage, ignited her passion for pressure injury and wound healing. With nearly 20 years of experience in a spinal cord unit and a research department, she has focused on at‐risk individuals and transitioned her emphasis from treatment to prevention. She has also been instrumental in establishing a multidisciplinary wound team, which provides education and information on pressure injury prevention and treatment to patients, district nurses and general practitioners.
**Dr. John Ivory, Ireland**	
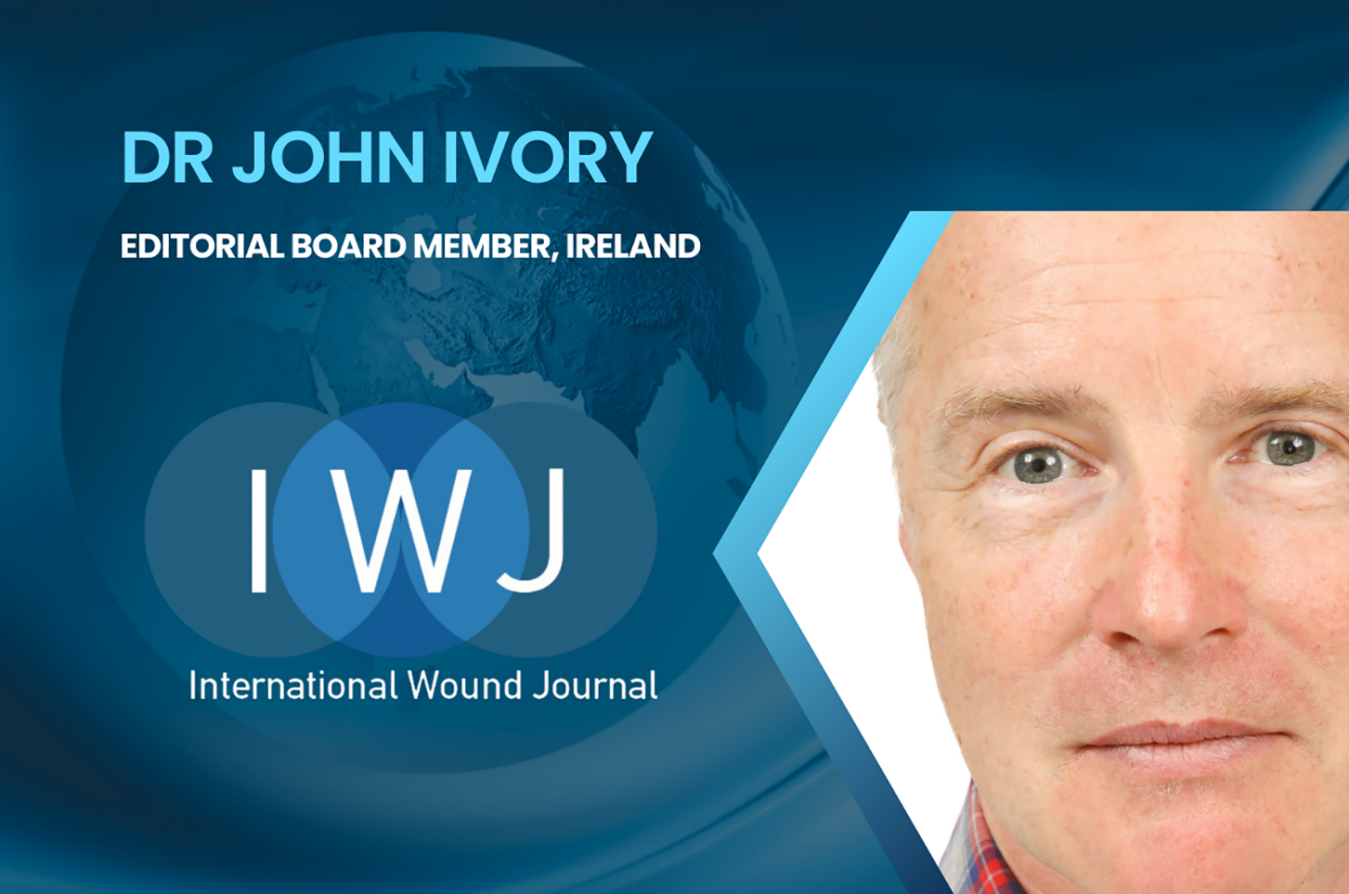	John has a BSc (Biochemistry), an MSc (Neuropharmacology) and is currently a PhD candidate, University of Galway, Ireland. John started out in wound care as a research assistant, spending over 2 years coordinating wound care clinical trials. In addition, he has accrued over 10 years of experience in the field of systematic/scoping reviews, developing reviews in such diverse areas as antiretroviral therapy, 5HT‐3 antagonists and profiling the venous leg ulcer patient. He is currently in the final year of a structured PhD program at the University of Galway, Ireland, investigating the diagnostic validity of clinical indicators reported in the literature to be indicative of biofilm in chronic wounds. He sits on the editorial board of the European Wound Management Association's Journal of Wound Management and has peer reviewed for that journal, The Journal of Tissue Viability and Wound Repair & Regeneration.
**Professor Agata Janowska, Italy**	
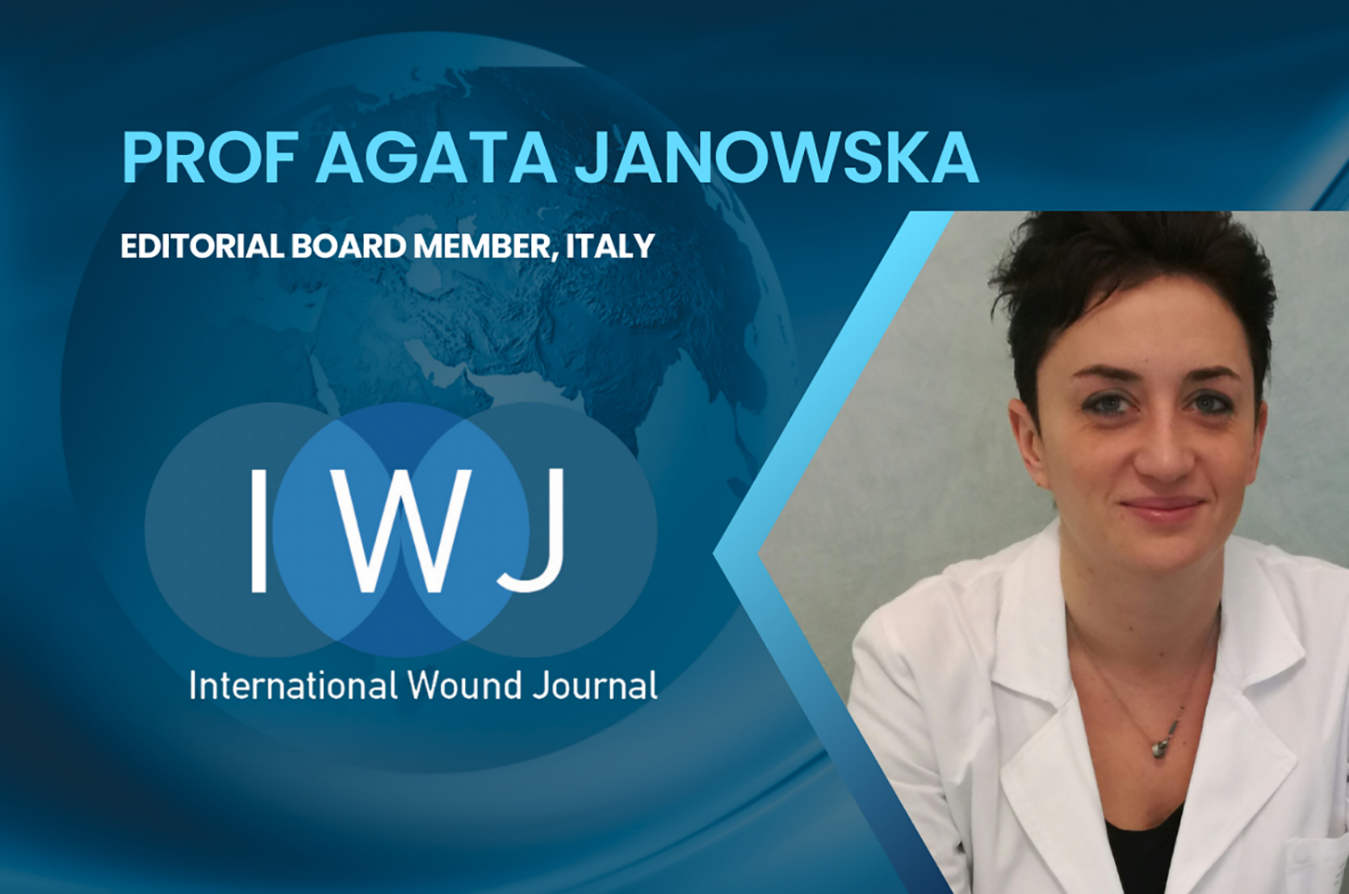	Dr. Janowska is a graduate of the University of Florence and received her MD in experimental dermatology from the medical faculty. She completed her residency in dermatology in Pisa and a fellowship at the Wound Healing Research Center. Currently she is assistant professor and consultant dermatologist at the Division of Dermatology, Department of Clinical and Experimental Medicine, University of Pisa, Pisa, Italy. At national and international levels, Dr. Janowska has served as member of multiple grant review panels and on the editorial advisory boards of multiple journals. She has extensive clinical experience of managing patients with wounds and skin tumours. Dr. Janowska's research activities include the development of biomaterials to be used on acute and chronic wounds, the implementation of different scaffolds for tissue engineering, and the elaboration of a prototype to non‐invasively assess acute and chronic wounds.
**Professor Wenguo Jiang, UK**	
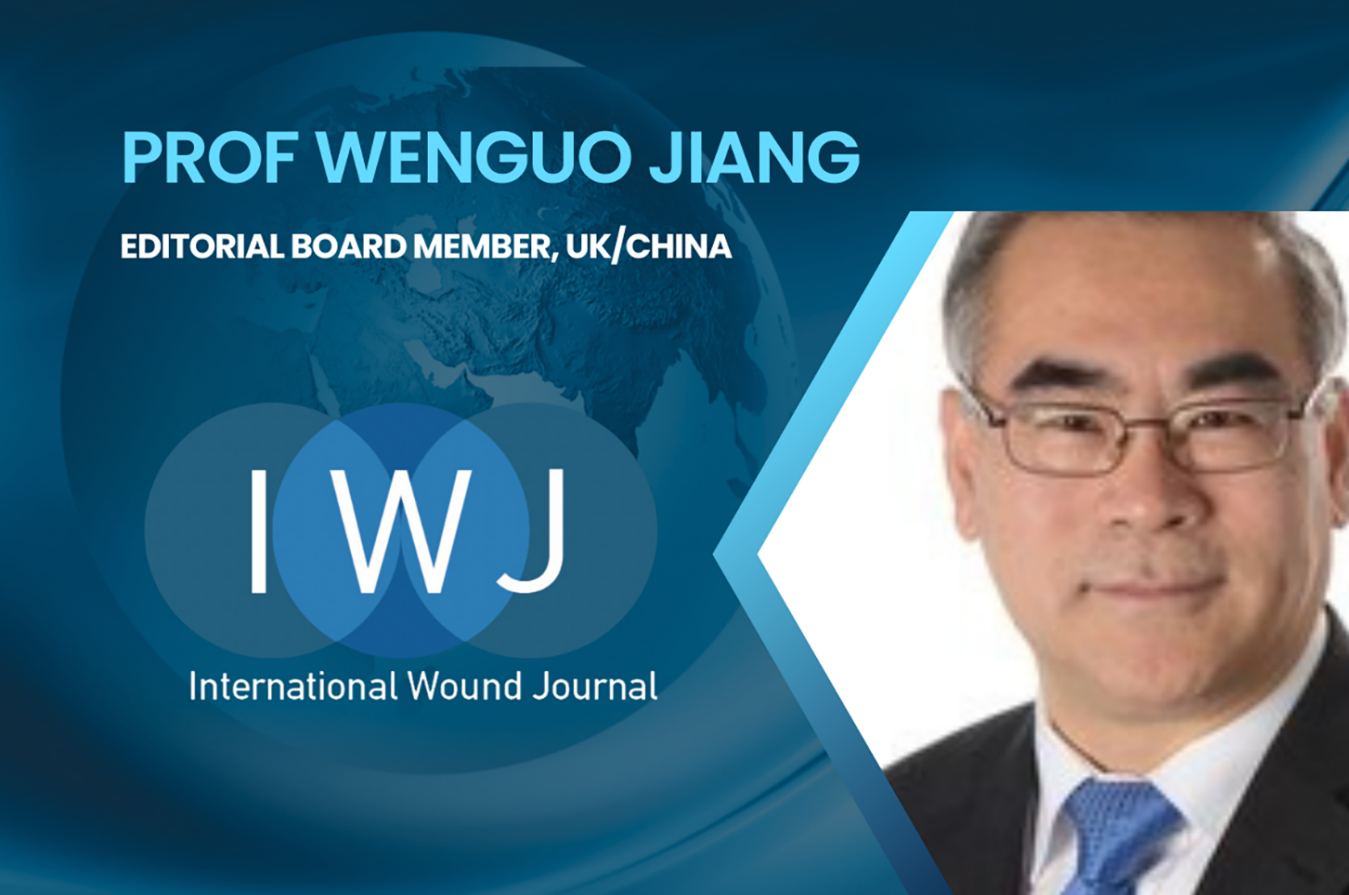	Demonstrating more than 20 years of expertise in the field of medicine, Dr. Wen Jiang is a central figure in medical research and education. He brings a plethora of skills in his current roles, as the Dean of International for Cardiff University, the Director of Cardiff China Medical Research Collaborative, Quality and Safety Improvement Faculty at the National Health Service, the Academic Director for International Relations and a professor of surgery and tumour biology at Cardiff University. Dr. Jiang is well‐known for his research in cancer metastasis, which he started in 1987. His research has centred on the molecular and cellular basis of cancer invasion and metastasis and therapeutic aspects of targeting cancer metastasis. He has published numerous publications concerning this research and his interest in the bone, peritoneal and brain metastasis from primary tumours including breast, prostate lung and GI cancers. Dr. Jiang works with colleagues all over the world to collaborate on at the Cardiff School of Medicine. He earned an M.B.BCh from Beijing Medical University in 1984 and an MD from the University of Wales in 1995. In addition to his work at Cardiff University, he has consulted for the Beijing Lung Cancer Centre, been an honorary professor at the Inner Mongolia Medical University and Capital Medical University.
**Professor Edward Jude, UK**	
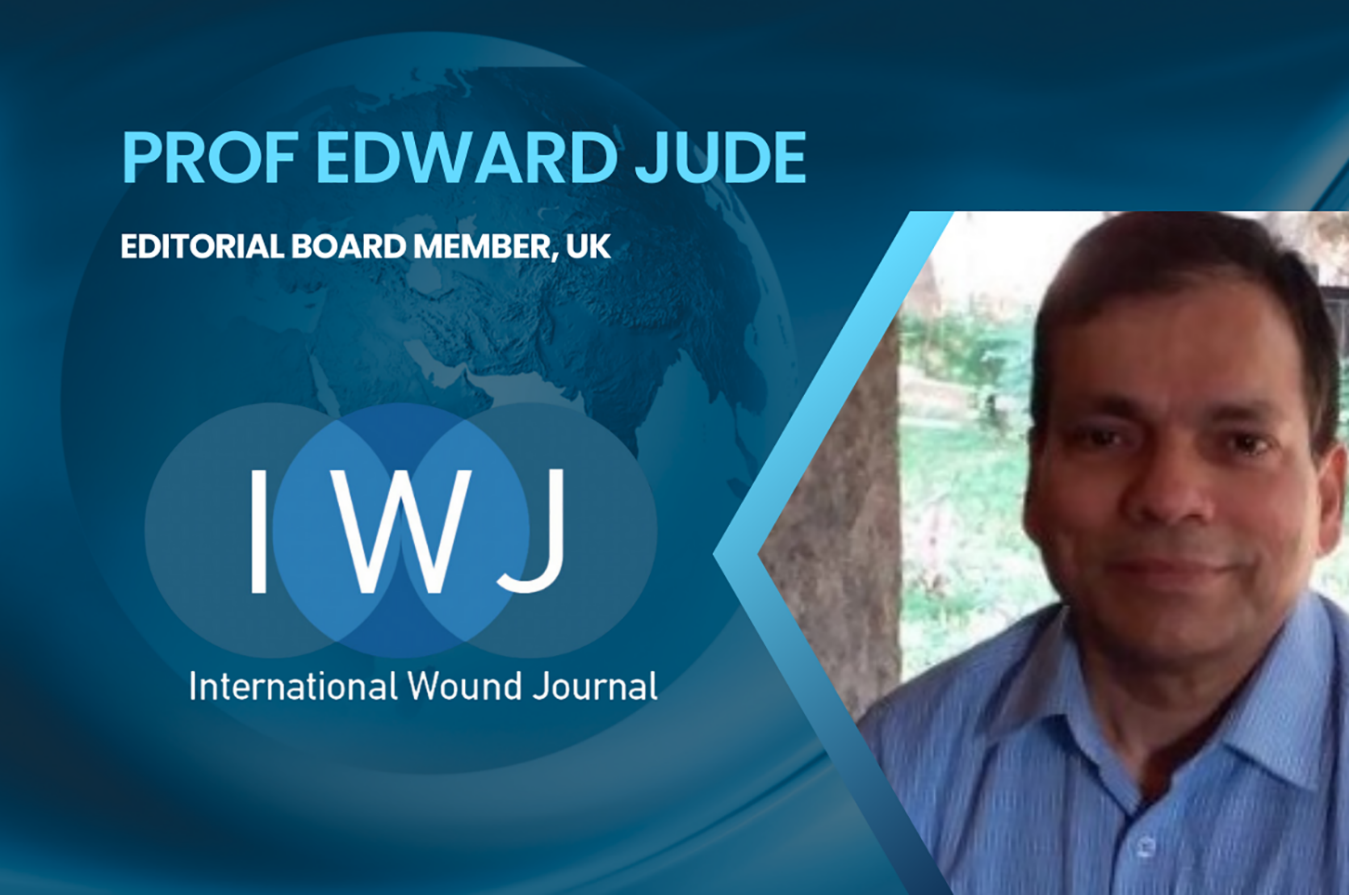	Professor Jude is Consultant Diabetologist and Endocrinologist, at the Tameside General Hospital, Ashton‐under‐Lyne, UK and Honorary Professor in Medicine at the University of Manchester and Honorary Professor at Manchester Metropolitan University. Having done his MD at Manchester University and then completing his training in diabetes and endocrinology in the North West, he took up his consultant post in 2001. He is active in research and has published over 220 original papers and chapters. He has secured grants from Diabetes UK, NIHRfPB, HTA, NIH (USA). His studies include Insulin trials, foot ulcers, painful neuropathy, Charcot foot and endothelial dysfunction in diabetic patients. He is past Chairman of the European Diabetic Foot Study Group and the Council for the European Wound Management Association. He is also the Lead for the North West Diabetes Group.
**Professor Ulrika Kallman, Sweden**	
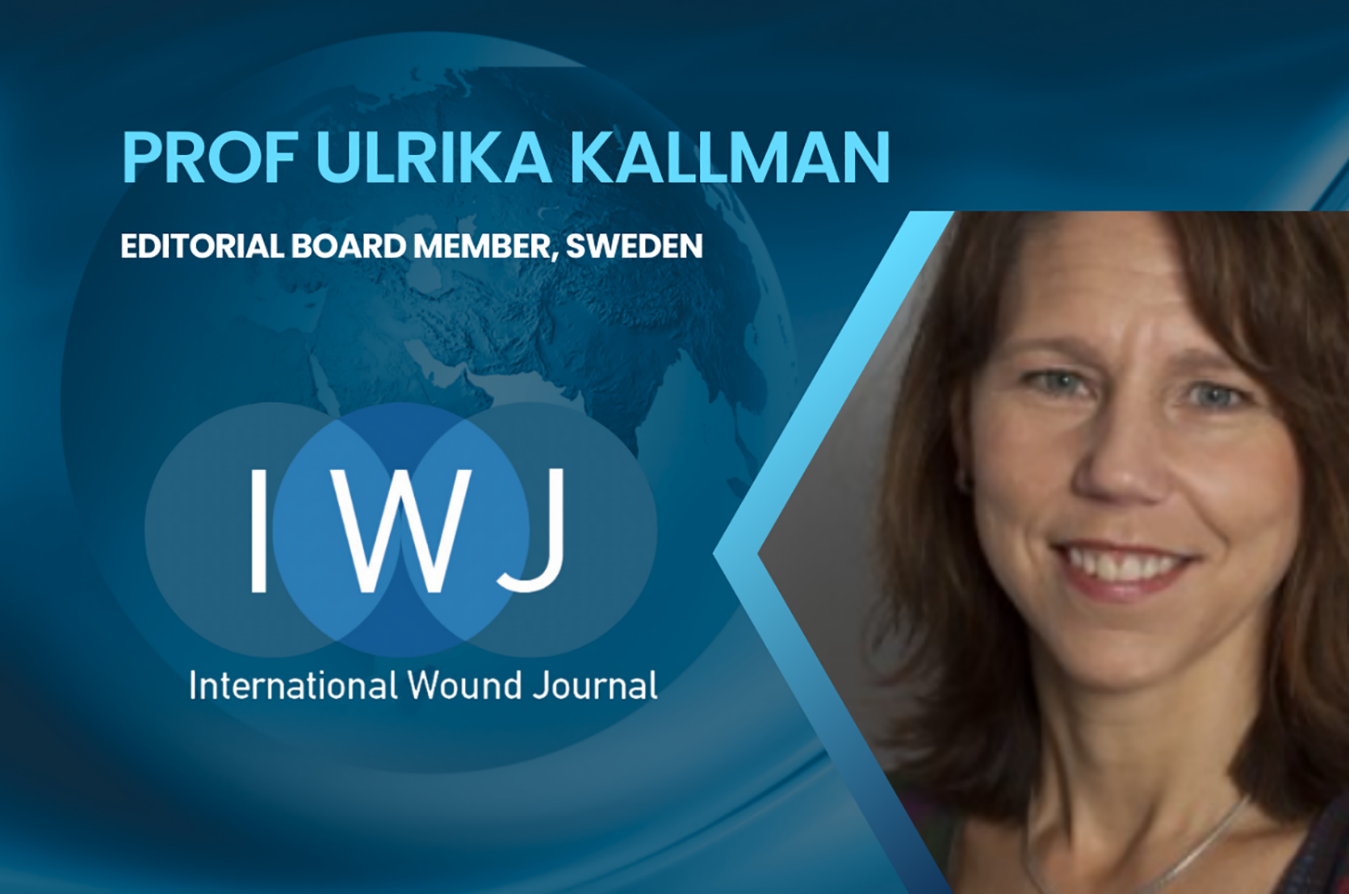	Dr. Källman, holds a PhD and is the Associate Professor, RN (operating theatre nurse). She works as the research manager at Department of Research and Education at Södra Älvsborg Hospital, Borås, Sweden. She is also, Adjunct Senior Lecturer at the Institute of Health and Care Sciences, University of Gothenburg, Sweden. Dr. Källman is a researcher in the broad area of patient safety, with interest in various aspects of wound prevention, especially pressure ulcers/injuries.
**Professor Peter‐Lars Kamotz, Austria**	
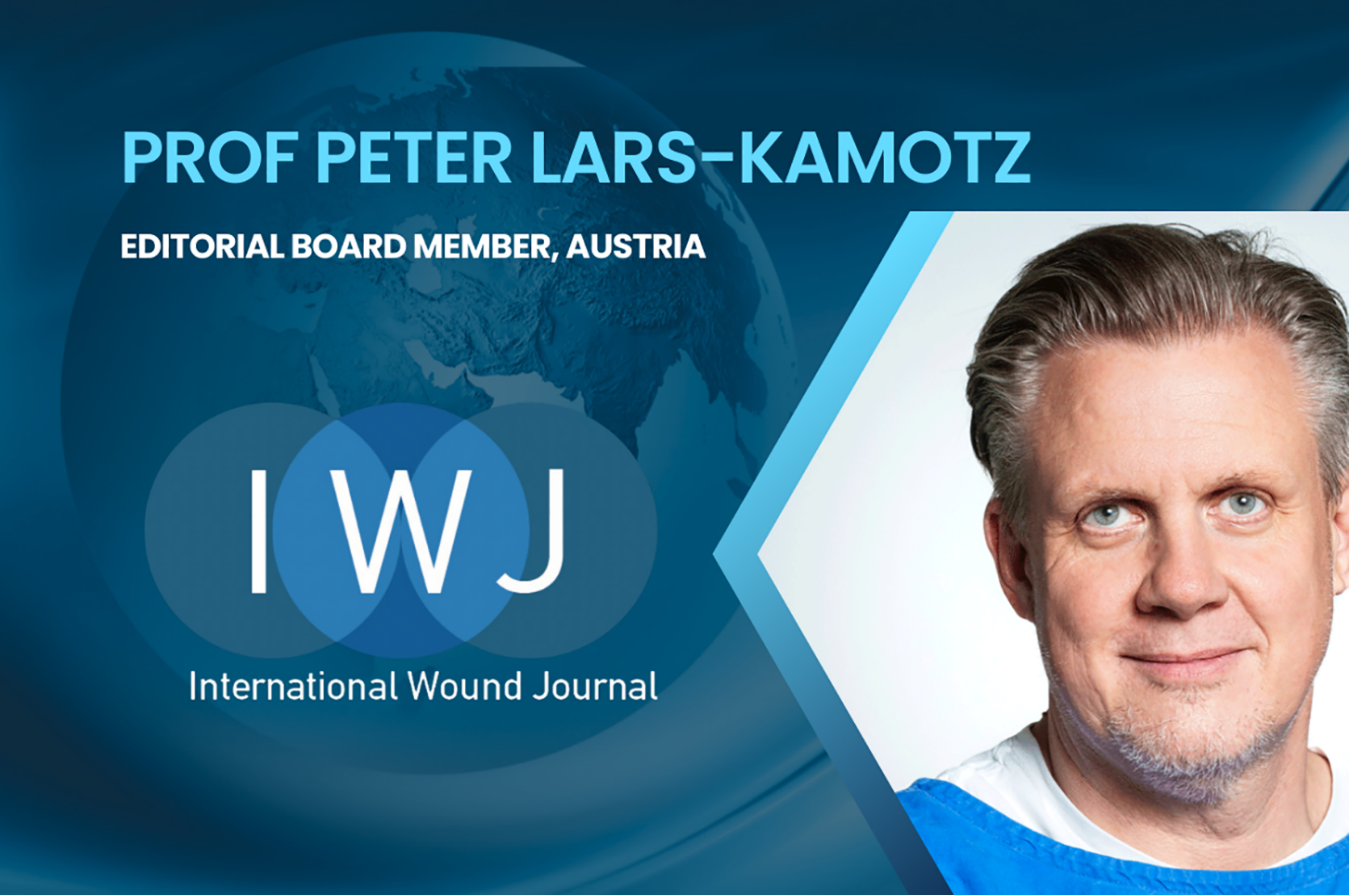	Dr. Kamolz is Professor for Plastic, Aesthetic and Reconstructive Surgery at the Division of Plastic, Aesthetic and Reconstructive Surgery, Department of Surgery, Medical University of Graz and Head of the Division of Plastic, Aesthetic and Reconstructive Surgery, Medical University of Graz, Austria. Furthermore, he is the Deputy Chief Medical Officer of the LKH‐University Hospital in Graz. Since 2018, he is also Director of COREMED—Centre for Regenerative Medicine and Precision Medicine at Joanneum Research Forschungsgesellschaft mbH. Since 2023, he is also Chair of the Department of Surgery, Medical University Graz. He is Editor and Member of the Review Board of several international Journals, Author of more than 280 peer reviewed article, editor of several books and book chapters. He was awarded several times from different scientific organizations for his scientific work.
**Professor Dimitris Kletsas, Greece**	
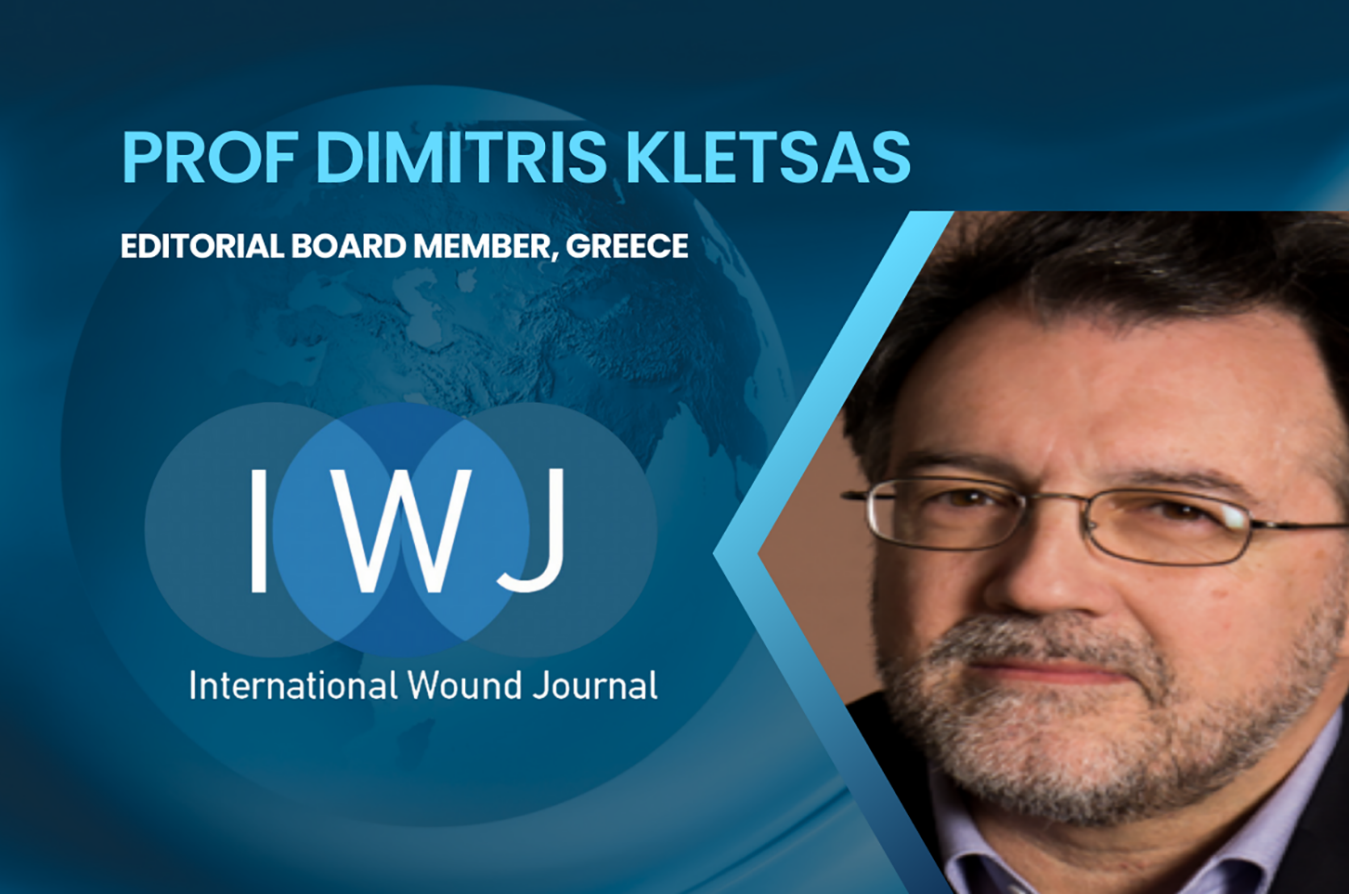	Dr. Kletsas holds a bachelor's degree in Biology for the University of Thessaloniki and a PhD in Biochemistry, Cell and Molecular Biology from the University of Athens. He worked as post‐doctoral or visiting scientist in the European Molecular Biology Laboratory (EMBL) in Heidelberg, in the Imperial College in London and in the Georgetown University in Washington DC. He is Research Director and Head of the Laboratory of Cell Proliferation & Ageing, of the Laboratory of Cell Systems and Bioactive Molecules and of the Experimental Animal Facility if the Institute of Biosciences and Applications of the NCSR ‘Demokritos’. Since 2017, he is Director of the Institute and Member of the Board of NCSR ‘Demokritos’. His research interests include: cellular senescence: molecular mechanisms and role in age‐related diseases; mechanisms of carcinogenesis; regulation of intracellular signalling pathways, and the study of natural products and new synthetic compounds with antioxidant/anti‐ageing, anticancer and wound healing action. He published 184 articles in peer‐reviewed journals and 16 chapters in books. He is Member of the Biosciences' Division of the National Council of Research and Innovation and on the National Committee for the Wellbeing of Experimental Animals, while he served as President in the Hellenic Society of Biochemistry and Molecular Biology and of the European Tissue Repair Society.
**Professor Jan Kottner, Germany**	
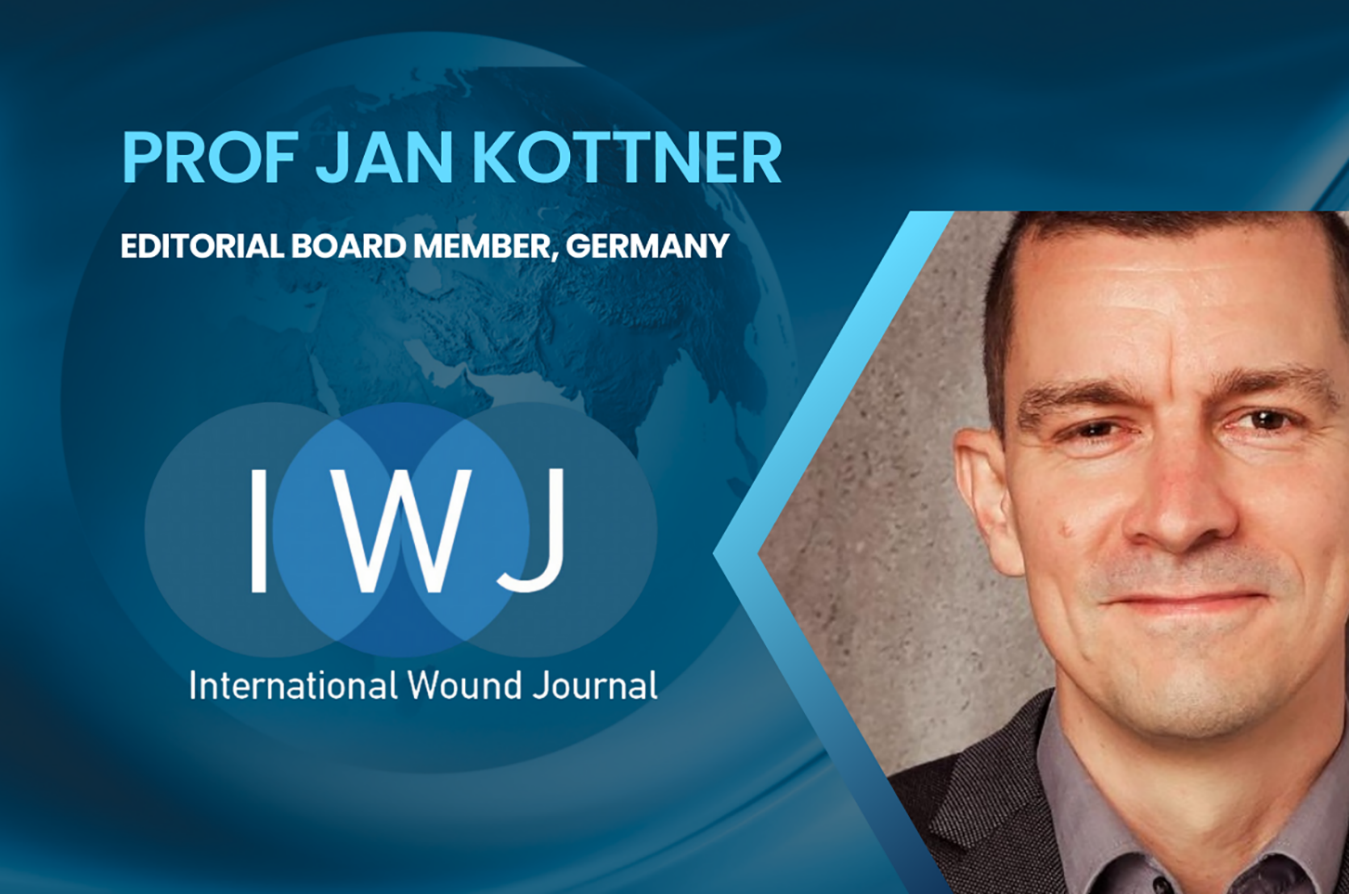	Dr. Kottner obtained his master in Nursing Science and Education in 2007 after having worked as registered nurse in several hospital settings. Measurement properties of pressure ulcer risk diagnoses and classification, statistical process control methods and the epidemiology of care problems were topics of his PhD thesis and his postdoctoral qualification. From 2011 to 2020 he was the Scientific Director of the Clinical Research Center for Hair and Skin Science at the Department of Dermatology and Allergy at the Charité‐Universitätsmedizin Berlin. Since 2020, he is director of the Institute of Clinical Nursing Science at the Charité—Universitätsmedizin Berlin. Key research interests of Jan Kottner are skin and tissue integrity and preventive skin care with a special focus on skin physiology, skin barrier restoration and maintenance. He has special interests in evidence‐based practice, including systematic reviews, guidelines, clinical trial design and conduct, and outcome development and validation. He is Associate Editor of the International Journal of Nursing Studies and member of international boards and societies focussing of skin and tissue health.
**Professor Jose Luis Lazaro‐Martinez, Spain**	
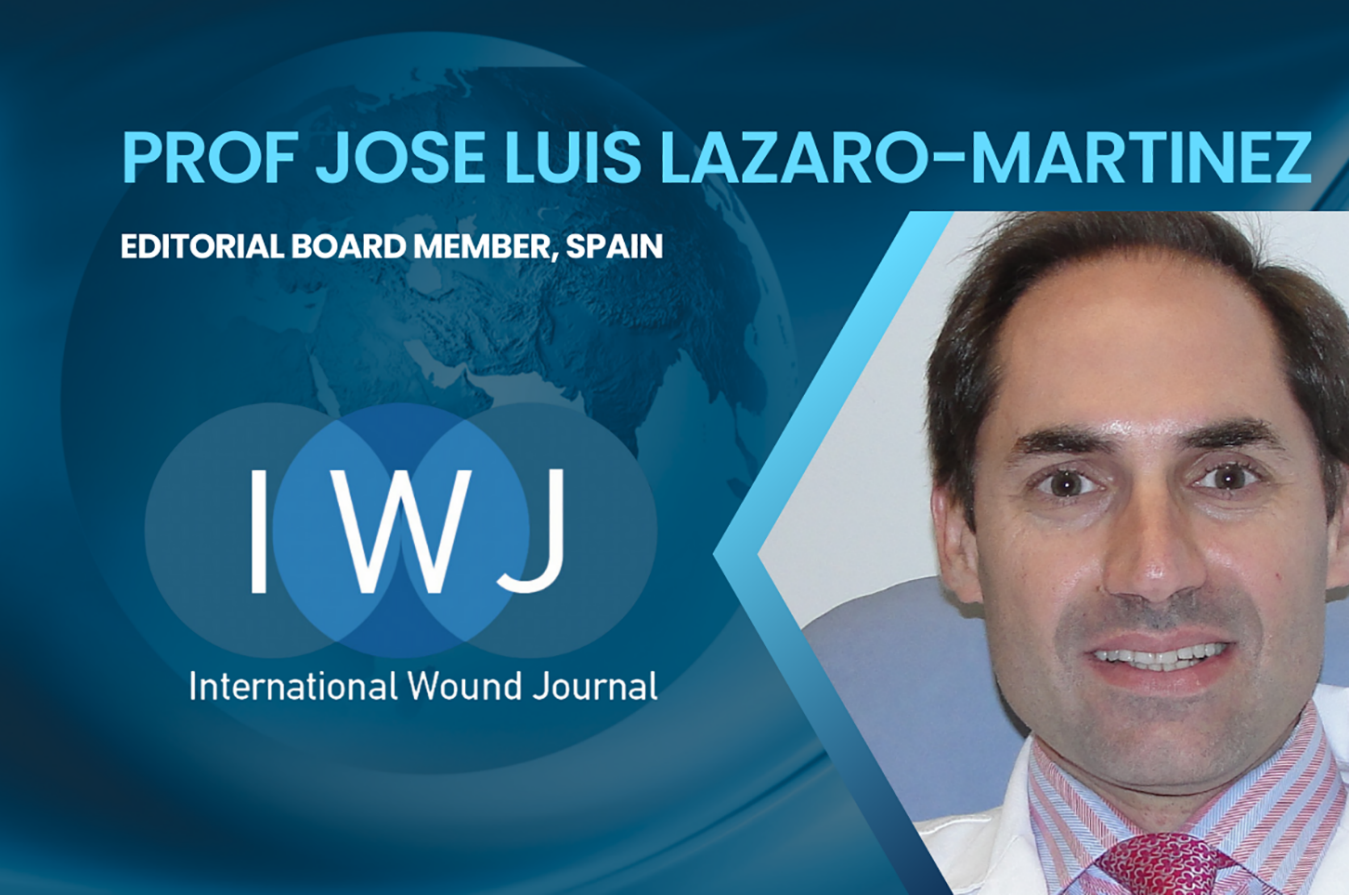	Prof. Lázaro‐Martínez is Full Time Professor at the University of the Complutense University of Madrid (UCM). He got his PhD degree at the same university doing his doctoral thesis on biomechanics of the diabetic foot. He is an expert in Podiatric Surgery certified by a Fellowship Board of the New York College of Podiatric Medicine. He holds a Master of Science Degree on Health's Research (MSc) by UCM. He is the Head of the Diabetic Foot Unit at UCM. He is Director of both Research Groups in diabetic foot at UCM and at the Research Institute of Health of the Hospital Clínico San Carlos in Madrid. He was the Coordinator of the Diabetic Foot Working Group of the Spanish Diabetes Society. He was the Scientific Secretary of the Diabetic Foot Study Group (DFSG) belonging to the European Association of the Study of Diabetes. He is Honorary President of Diabetic Foot International (International Working Group on the Diabetic Foot Implementation). He is a member of the Editorial Committee of the Journal Diabetic Foot & Ankle, Journal Clinical Medicine and Frontiers Endocrinology. He has numerous publications in international journals indexed in the journal citation report (JCR‐more than 140). He has conducted 20 doctoral theses (PhD) in the field of diabetic foot at the Complutense University of Madrid. He has participated in more than 350 conferences at the national and international level.
**Professor Richard Leigh, UK**	
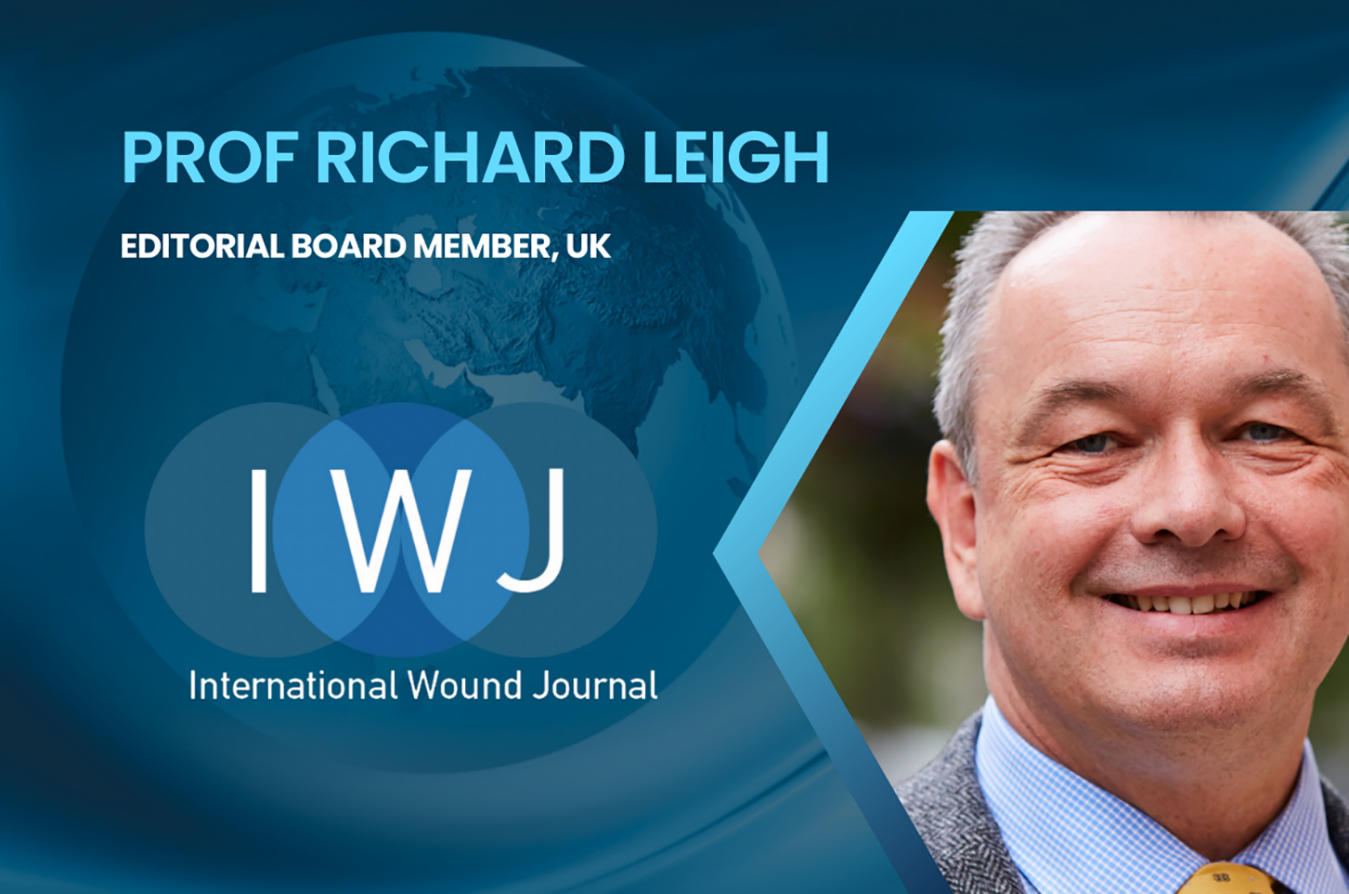	Richard Leigh is Consultant Podiatrist at the Royal Free Hospital. He is visiting Professor to Perm State Medical University. He has particular expertise in acute foot care, including foot conditions related to diabetes, vascular and neurological pathology.
**Ellie Lenselink, Netherlands**	
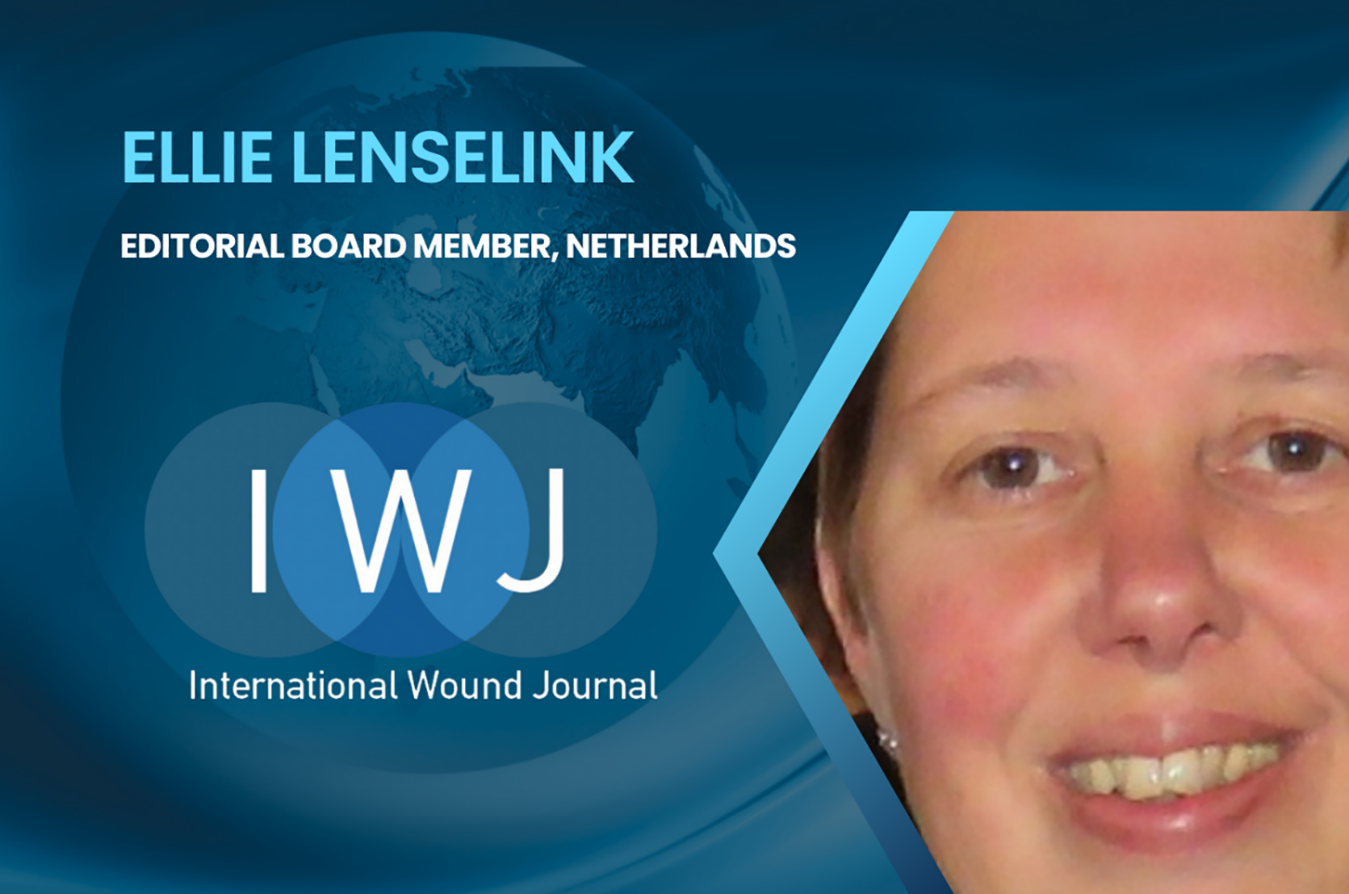	Ellie Lenselink is a dedicated inspired Wound Consultant nurse who loves to combine clinical care with medical science. During her carrier, she always want to expand her knowledge in order to give the patient the best possible high‐quality care. After graduating from the MSc in Wound Healing & Tissue Repair, she continued doing some research next to her work as a Wound Consultant nurse. As a member of the multidisciplinary wound team, she was one of the driving forces and founders of the multidisciplinary Wound Expert Clinic in The Hague's largest teaching hospital: Haaglanden Medical Centre. Together with her colleagues, she takes care of patients with complex wounds and is specialized in diabetic foot wounds. In addition, Ellie is a tutor at the Dutch course for wound Consultant, auditor for the Dutch college of healthcare training and auditor for the Dutch association for Nurses. Ellie had published several articles both in Dutch and international.
**Dr. Natasha Levy, UK**	
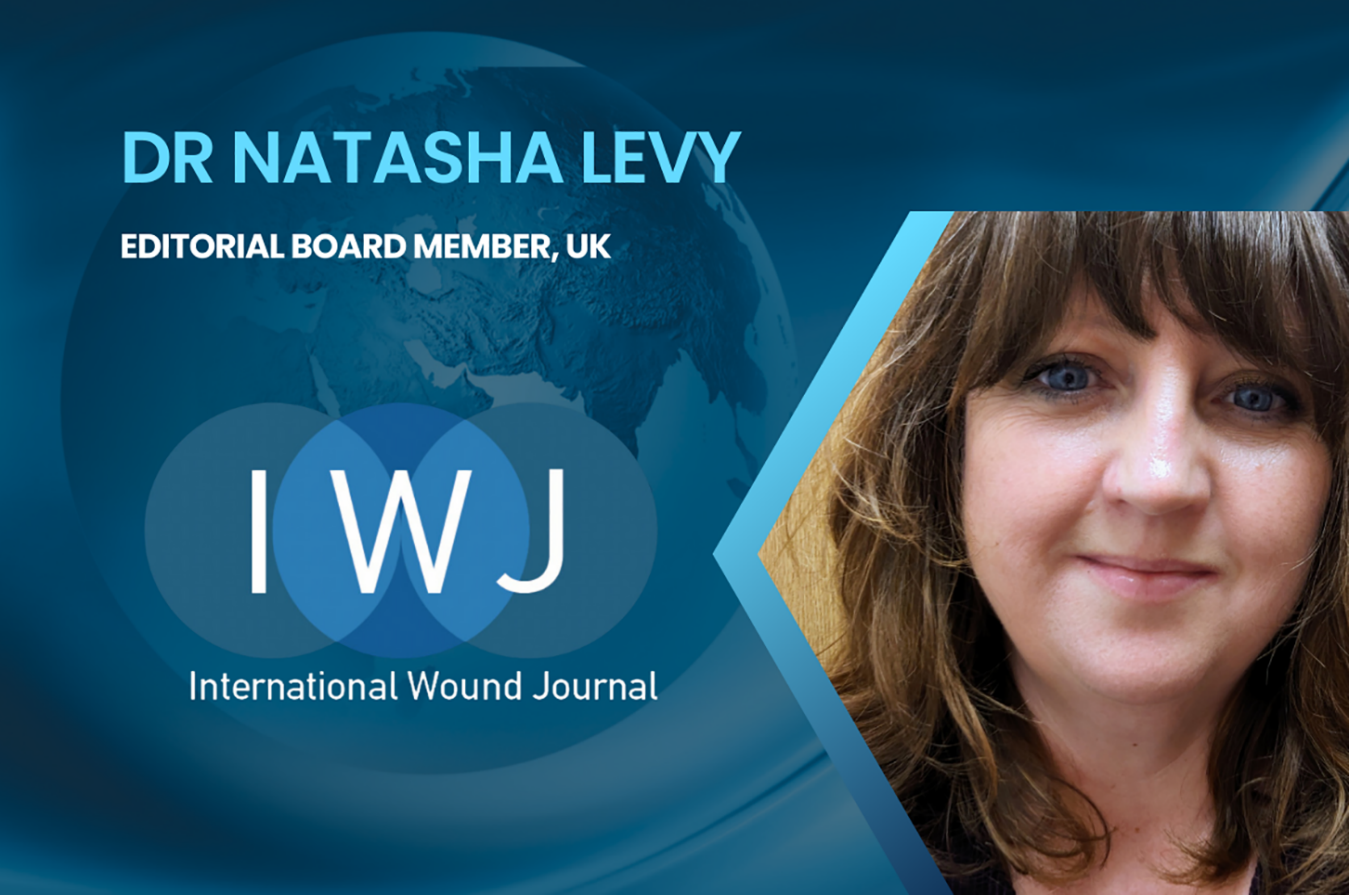	Dr. Levy obtained a BSc (Hons) Podiatry 1997, University of Huddersfield; MSc Clinical Practices for Health Care Professionals 2004, University of Salford; PGCert Professional Development (Higher Education Practice) 2011 University of Huddersfield; and a PhD in 2023, University of Huddersfield (Thesis Title ‘What is the Impact of Below Ankle Amputation upon Quality of Life for Individuals with Type 2 Diabetes Mellitus?’). Dr. Levy is a senior lecturer in the school of human and health sciences at the University of Huddersfield, where she is involved with both the clinical and theoretical education of health care professionals and research focussed upon enhancing patient care. She is also an elected Trustee for the Society of Tissue Viability, an independent charity focussed upon encouraging collaborative thinking to solve wound and skin care challenges through evidence‐based education for all involved with tissue viability. Dr. Levy's research focusses upon enhancing clinical care and quality of life for patients with compromised tissue viability, exploring aspects such as the long‐term complications of diabetes, peripheral arterial disease, and amputation. Dr. Levy has been a Co‐investigator on Medical Research Council Grant awarded research exploring the development of new technologies to enhance assessment and care of individuals with PAD.
**Dr. Maria Lopez‐Franco, Spain aura Parnell, USA**	
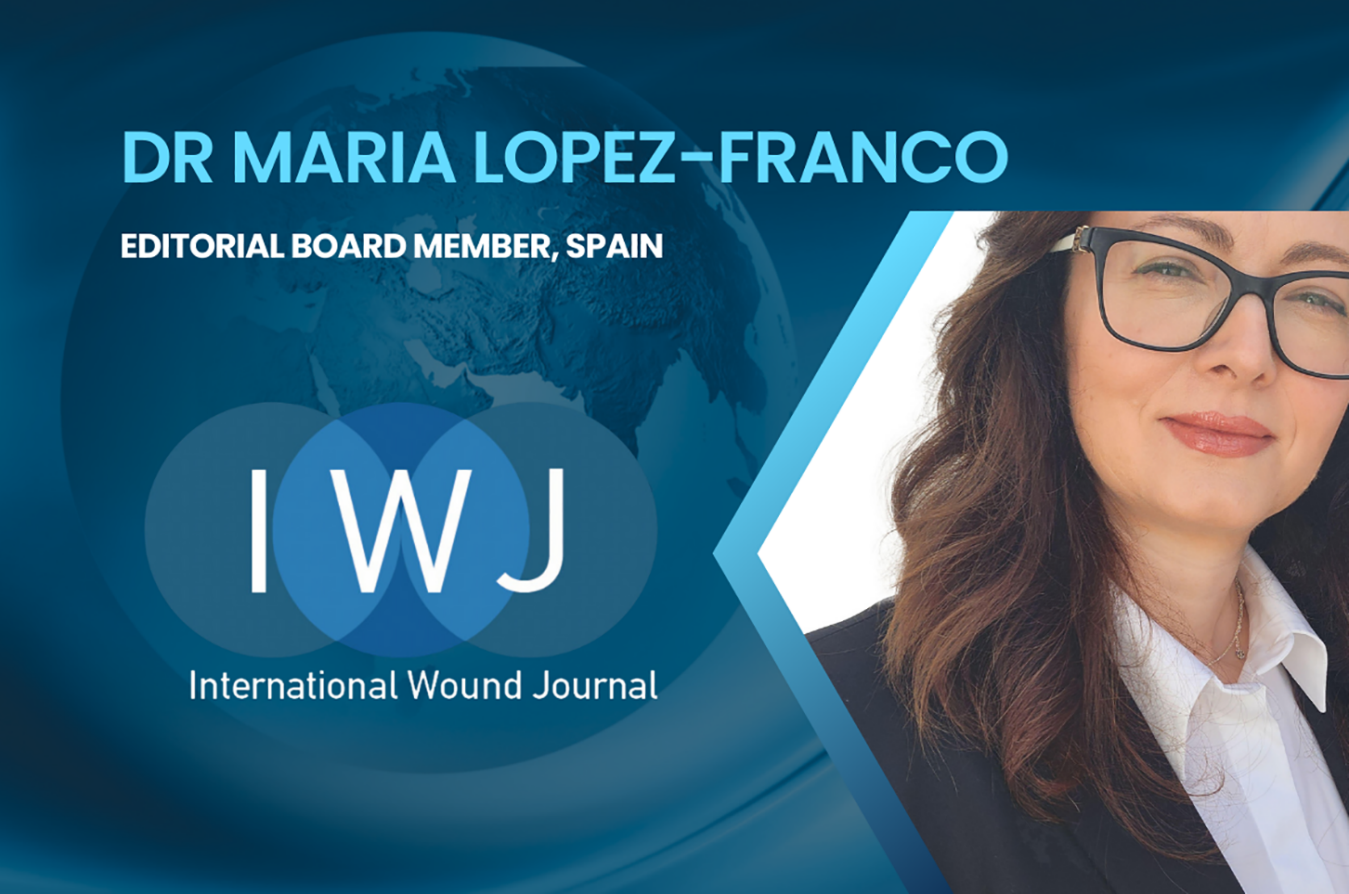	Maria is a nurse in Primary Care at Centro Salud Linares A Virgen de Linarejos and Centro de Salud Linares C San José (2014); a nurse in the Emergency Department of the Hospital San Agustín de Linares (2015); and the Medical‐Surgical and Neurotraumatological Hospital of Jaén. July, August, December (2016–2018). She is currently assistant lecturer at the University of Jaén. She was heavily involved in a multi‐centre study: Prevention of the adverse effect of pressure ulcers in the framework of patient safety: knowledge, attitudes and perceived barriers in nursing professionals (SECOACBA Project) 2015–2019. She also drove the sixth national study on the prevalence of skin lesions related to dependency (2022).
**Professor Daniel López‐López, Spain**	
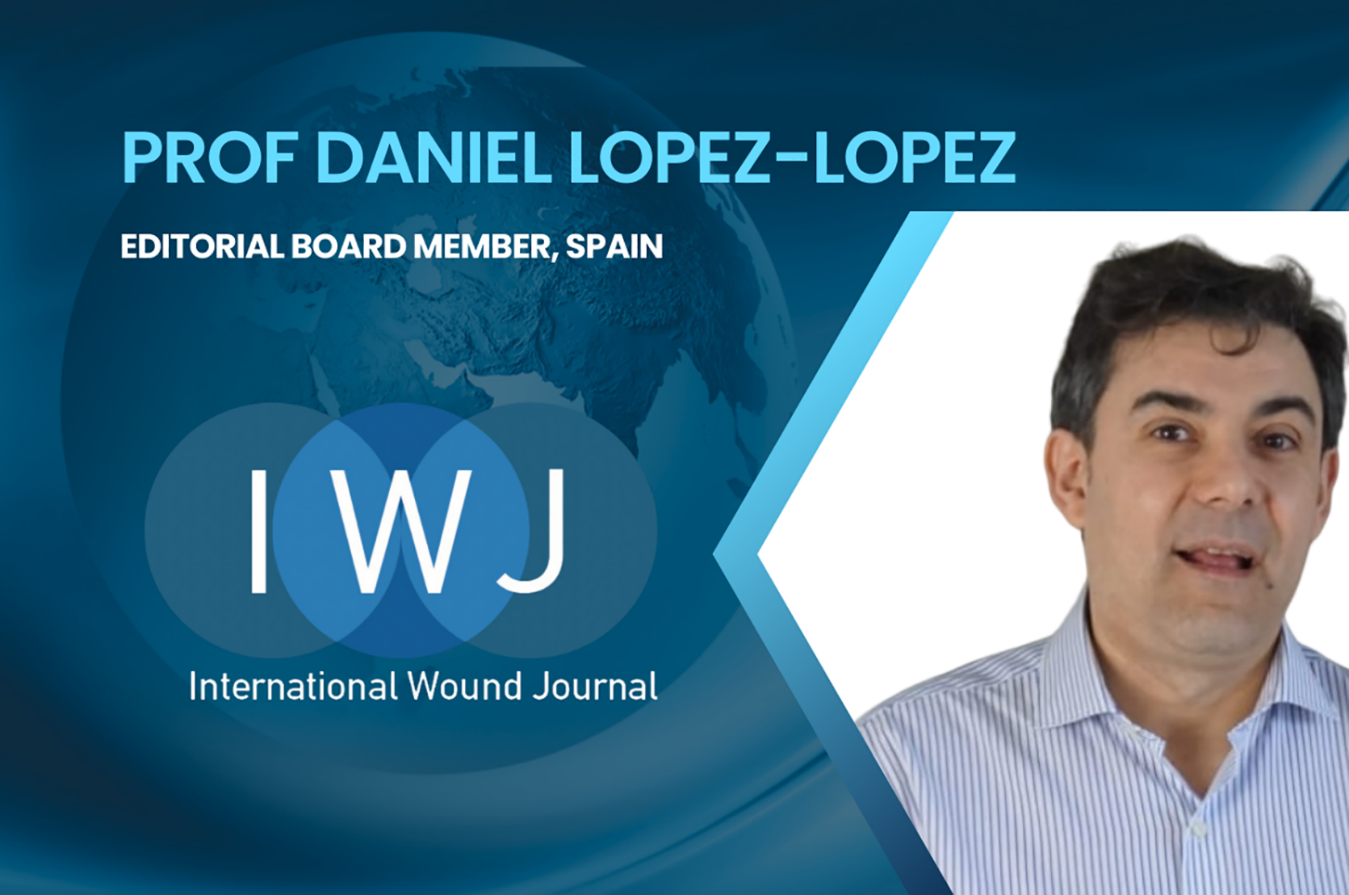	Professor. Daniel López López. Coordinator of Research, Health and Podiatry Group. Department of Health Sciences. Faculty of Nursing and Podiatry. Industrial Campus of Ferrol. Universidade da Coruña, Ferrol, Spain.
**Dr. Chris Manu, UK**	
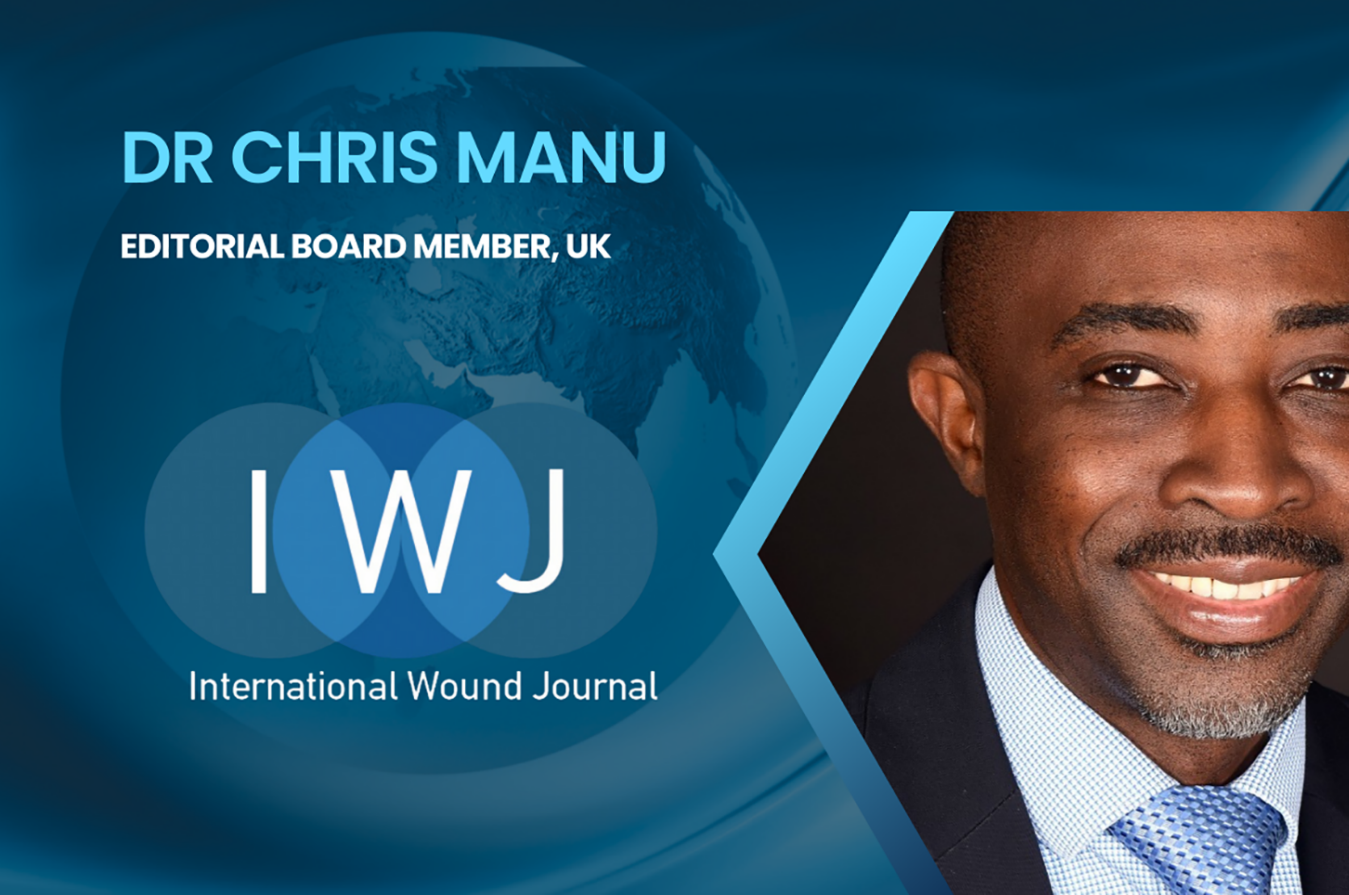	Chris has been a consultant at King's College Hospital, London, since 2016. But even while undertaking his specialist training, he has been involved in several service improvement projects within the South East London (SEL) area, and setting up new foot care pathways. He has research interest in peripheral arterial disease and has undertaken an MD on vascular risk stratification of people presenting with foot complications. He has been proactive in the setting up and running of the SEL MDfT Network, and the Clinical Lead for their Amputation Root Cause Analysis. In his role as Diabetes UK Clinical Champion, Chris led on developing an improved access to diabetic foot surgical procedures, a problem that has become even more critical post COVID‐19 pandemic. This aims to deliver prompt ‘one‐stop’ assessment and ‘day‐case’ surgical procedures within a clinic setting and to improve on waiting lists, avoid hospital admissions and decrease hospital length of stay.
**Professor Jenni McDonald, UK**	
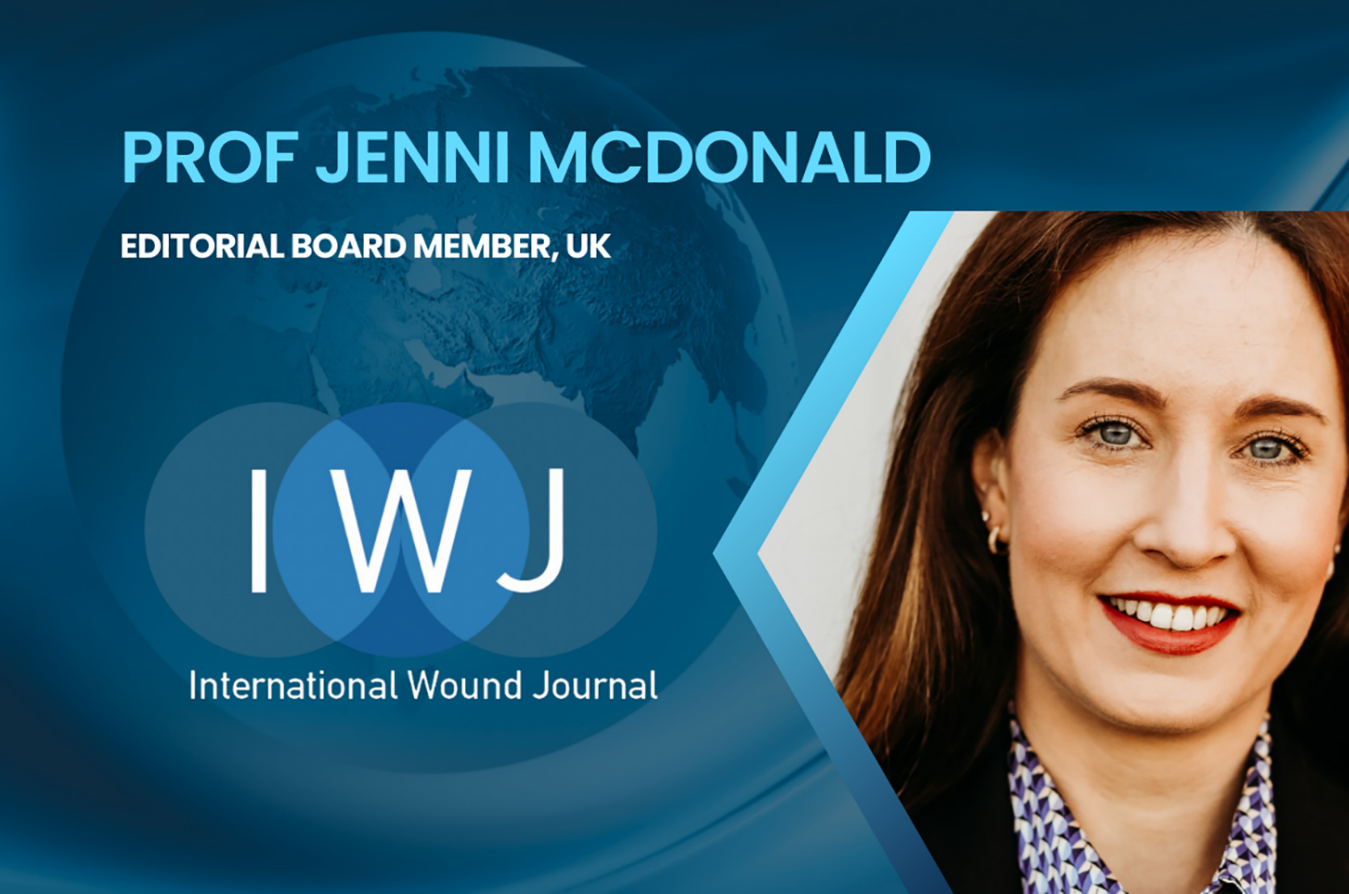	Prof MacDonald is an Executive Nurse Director at Pioneer Wound Healing and Lymphoedema Centres and currently leads the largest wound healing team in the United Kingdom. Jenni also holds the role of Professor of Wound Healing at Brimingham City University, supporting the delivery of Masters degrees in wound healing and tissue repair. Jenni continues to practice as a Nurse Consultant in Tissue Viability and over the last decade has led Tissue Viability and Harm Free Care services for acute, primary and community care across both England and Scotland. She completed her Bachelor of Science degree in Tissue Viability in 2011, Master of Science degree in Skin Integrity in 2017 and graduated from her Darzi Fellowship (PGDip) in 2018, which is a prestigious, high‐profile programme designed to develop leaders to undertake complex change initiatives in health and social care. Jenni has been heavily involved in several wound care publications, and in 2024, she was appointed as the Skin Integrity Nurse Consultant for the UK Sports Institute.
**Professor Chris Morriss‐Roberts, UK**	
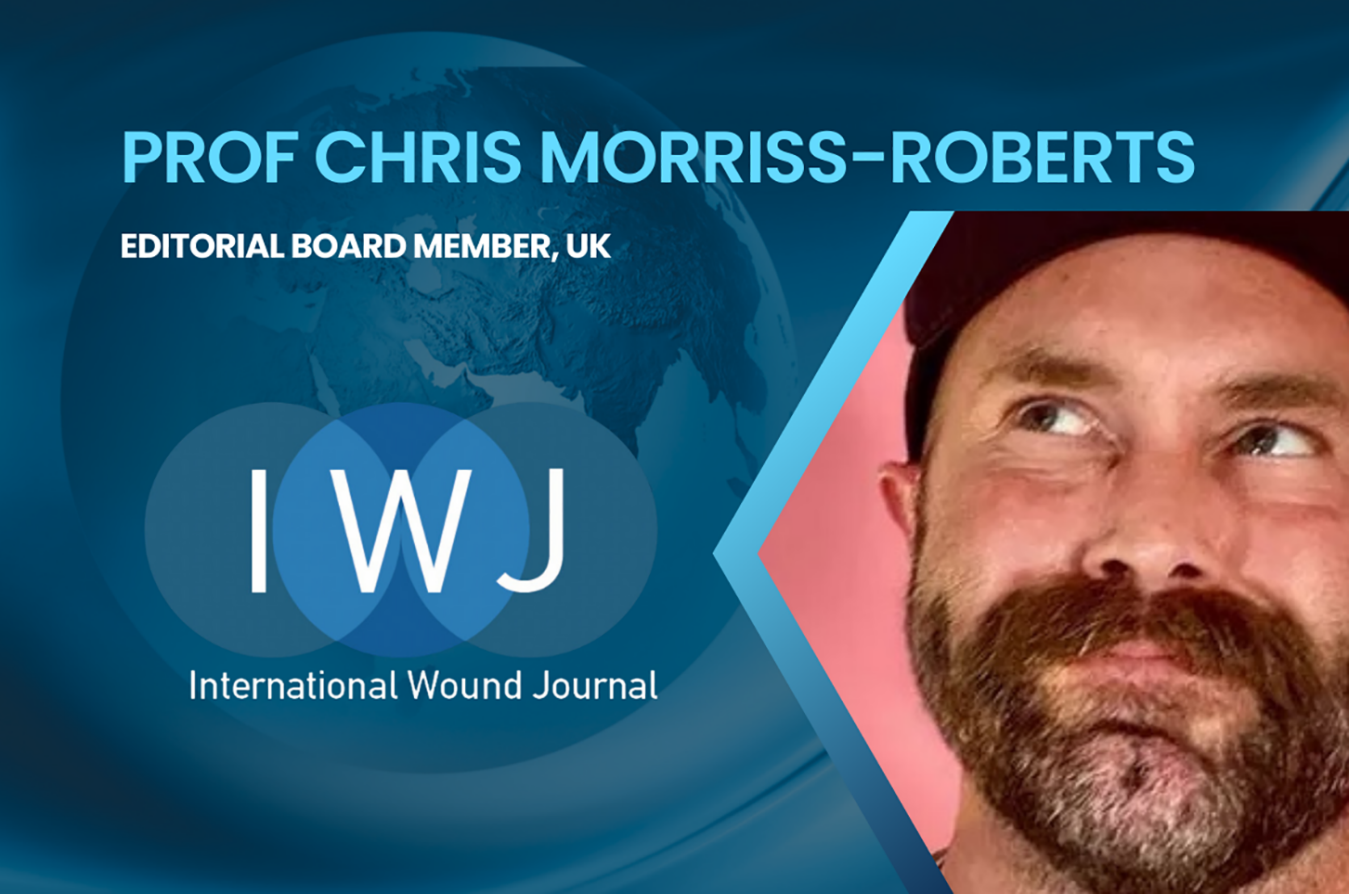	Dr. Morriss‐Roberts started his career as a Podiatrist, studying BSc (hons) Podiatry at The University of Southampton. Chris has worked as a podiatrist in both the private and public sector of healthcare. Chris moved into higher education after working as a clinical educator in the then Westminster PCT. Chris has worked at the University of East London, the University of Brighton and is currently Associate Professor and Deputy Health of Health at London Metropolitan University. While at the University of Brighton, Chris led the postgraduate programmes in health, including Podiatric Surgery, Rheumatology, MSK and independent Prescribing. Additionally, Chris was the lead for the research agenda/training across podiatry at both undergraduate and postgraduate levels. He designed a research programme that enabled students and staff to publish together—providing a mechanism through which to increase REF output and employability skills for undergrads and postgrads. Chris has supervised over 60 MSc students complete their dissertations, as well as eight PhD students complete the doctoral studies. Chris has published widely with over 100 papers, conferences and talks. Chris worked at Great Ormond Street Hospital as a Senior Qualitative Research Fellow. He undertook a hospital‐wide service evaluation, undertook research looking at mental health in the children with 22q syndrome, in additional to exploring the experiences of children living with cancer. Chris has been an active member of the Royal College of Podiatry for over 15 years, sitting on various committees, initiating the EDI committee, enjoying an extended term on the board of directors. Chris was delighted to take the former PodNow under his wing and transform it into ‘The Podiatrist’ in his role as the Chief Professional Editor.
**Professor Tom O'Connor, Ireland**	
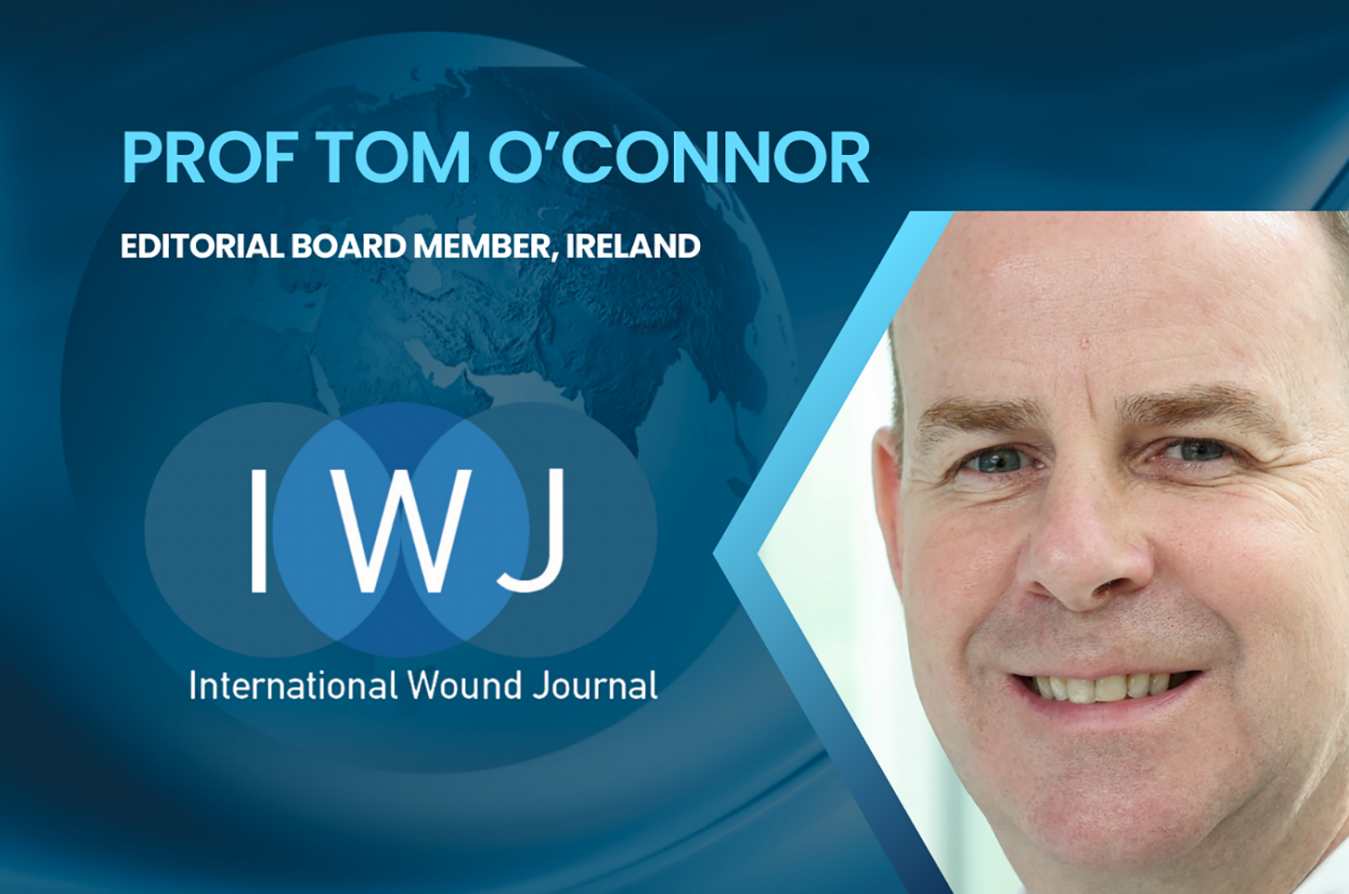	Professor O'Connor is a Registered Nurse, a Registered Nurse Tutor and is a committed nurse academic with many years of experience in nursing and midwifery education. Prof O'Connor has clinical practice experience in the Netherlands, Australia and Ireland and is a graduate of the Royal College of Surgeons in Ireland, holding Bachelors in Nursing Studies. He also holds a Higher Diploma in Education, a Master's of Science in Advanced Nursing from the University of Ulster and completed an Education Doctorate in the University of Keele in the United Kingdom. As Director of Academic Affairs in the RCSI School of Nursing & Midwifery, Prof O'Connor oversees and has developed a broad range of education opportunities for post‐graduate specialization for nurses and midwives. Prof O'Connor is a committed researcher, with over 100 published papers in wound care and other areas. He is an elected Trustee of the and Chair of the Scientific Committee of the European Pressure Ulcer Advisory Panel and holds honorary appointments in Griffith University, Queensland, Australia, Fakeeh College of Health Sciences, Jeddah, Saudi Arabia and Lida Institute, Shanghai, China.
**Professor Karen Ousey, UK**	
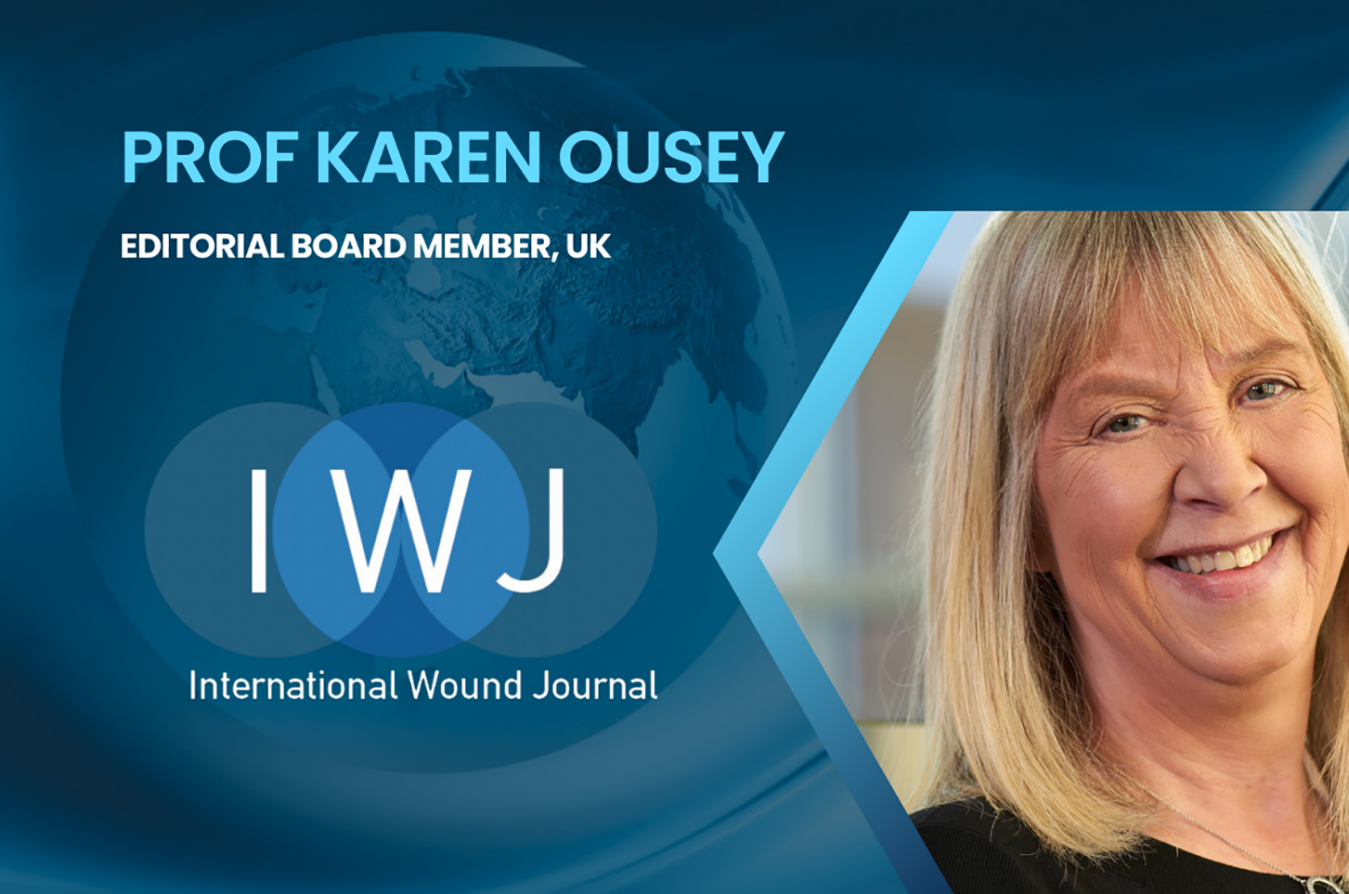	Karen is Professor of Skin Integrity and Director for the Institute of Skin Integrity and Infection Prevention. She is also Visiting Professor in the School of Nursing, Faculty of Health at the Queensland University of Technology, Australia and Visiting Professor at the Royal College of Surgeons, Dublin, a Florence Nightingale Scholar, chair of the International Wound Infection Institute, member of NHS National Wound Care Strategy, past academic editor for Wounds UK and is an editorial board member for the Journal of Wound Care. Karen led development of TVLC, the first UK wide Tissue Viability Service Competency framework. Karen's clinical background is in orthopaedics and tissue viability. She has worked in NHS hospitals in the North West of England and in London.
**Professor Massimo Papi, Italy**	
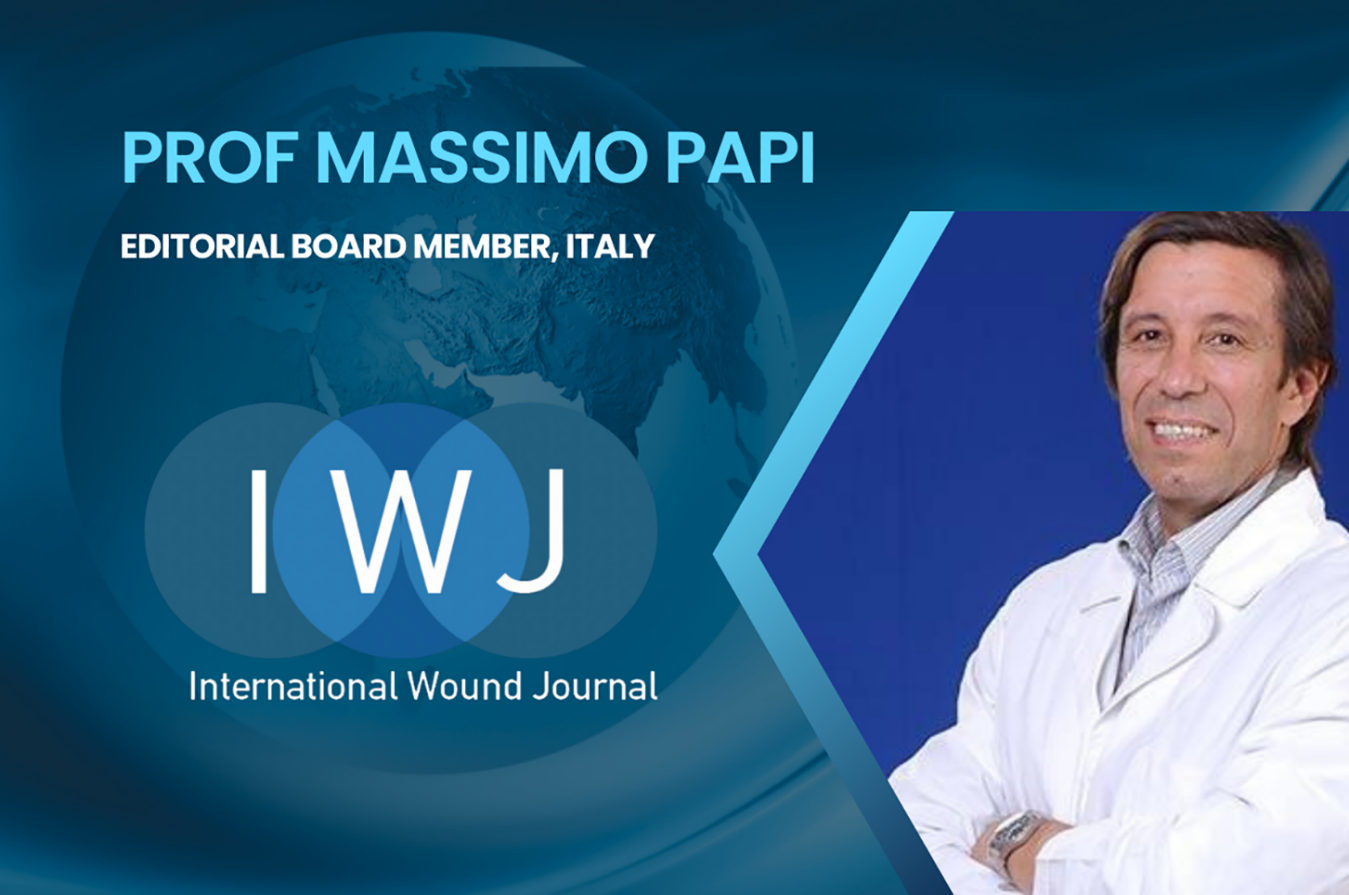	Graduated in Medicine and Surgery in 1983 and specialized in Dermatology since 1986, Professor Massimo Papi started working as a Dermatologist at the Dermopathic Institute of the Immaculate (I.D.I.) in Rome, where Massimo worked for 30 years and held various positions (Head of the high specialized unit on skin wounds, Technical‐Scientific Committee, Ethics Committee). Massimo has published several medical texts on skin ulcers, chronic wounds, and vasculitis and various book chapters on skin infections, psoriasis, vasculitis, ulcers, dermatological therapy and dermatological surgery, about 200 scientific articles in national and international journals and over 400 presentations and conference readings ‘Peer reviewer’ of Archives of Dermatology, Journal of European Academy of Dermatology, International Journal of Lower Extremity Wounds. Massimo is Associate Editor in International Journal of Lower Extremity Wounds (IJLEW) since January 2018. Massimo is a founding member of the Italian Association for the Treatment of Skin Ulcers (AIUC) and initiator of the service for chronic wounds of the IDI in Rome Creator and organizer of DERMART (2009 ➔ 2024) medical‐scientific conference that combines the observation and interpretation of clinical dermatology with visual arts.
**Professor Andrea Pokorna, Czech Rep**	
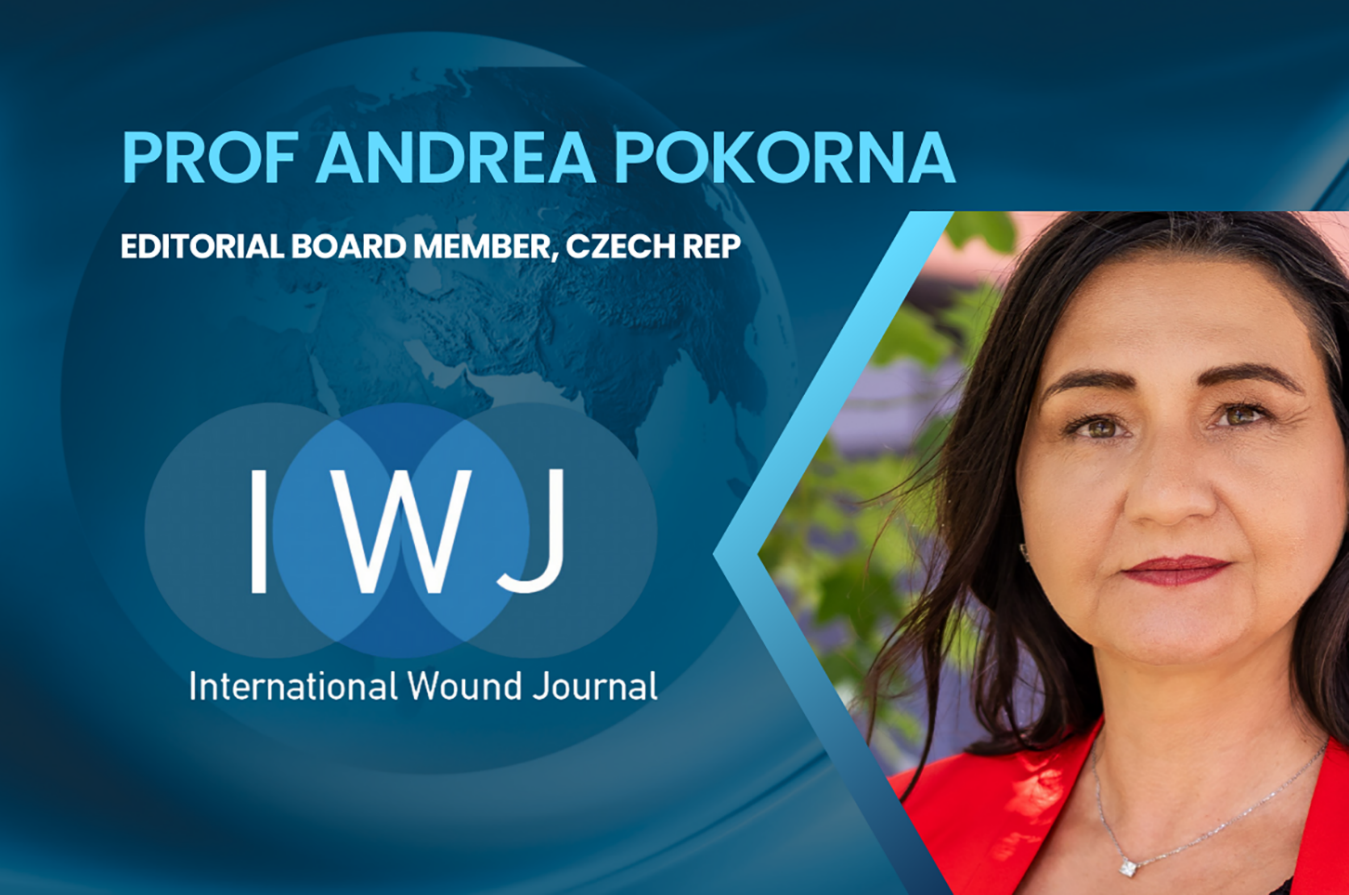	Professor Pokorna is a senior lecturer and professor in nursing at the department of Nursing, Masaryk University, and faculty of Medicine in the Czech Republic. She is also a trustee of the Czech Wound Management Association and a member of the expert for pressure ulcers expert panel by the Ministry of Health of the Czech Republic (organizing preventive measures on national level, national and international education and scientific events and sharing evidence‐based knowledge and skills not only in wound management). Working as a head of department for evaluation of quality of care on national level at Institute of Health information and statistics in the Czech Republic (IHIS CR), she represents in OECD (healthcare quality indicators group). Professor Pokorna has been involved in several national and international projects focusing on wound management, pressure ulcers prevention and treatment, nursing empowerment and nursing education, by mentoring not only in wound management. She is involved in EPUAP & EWMA Joint advocacy project and particularly interested in classification and scoring systems in nursing in general (e.g., pressure ulcer classification, differentiation in nursing diagnostic and appropriate symptomatology management—intensive care and elderly care particularly, science implementation, etc.) Currently, Professor Pokorna collaborates with Skin Integrity Research Group (SKINT) in the development and psychometric testing of instruments to measure the outcomes of nursing interventions related to pressure ulcer prevention and IAD identification and treatment.
**Professor Anna Polak, Poland**	
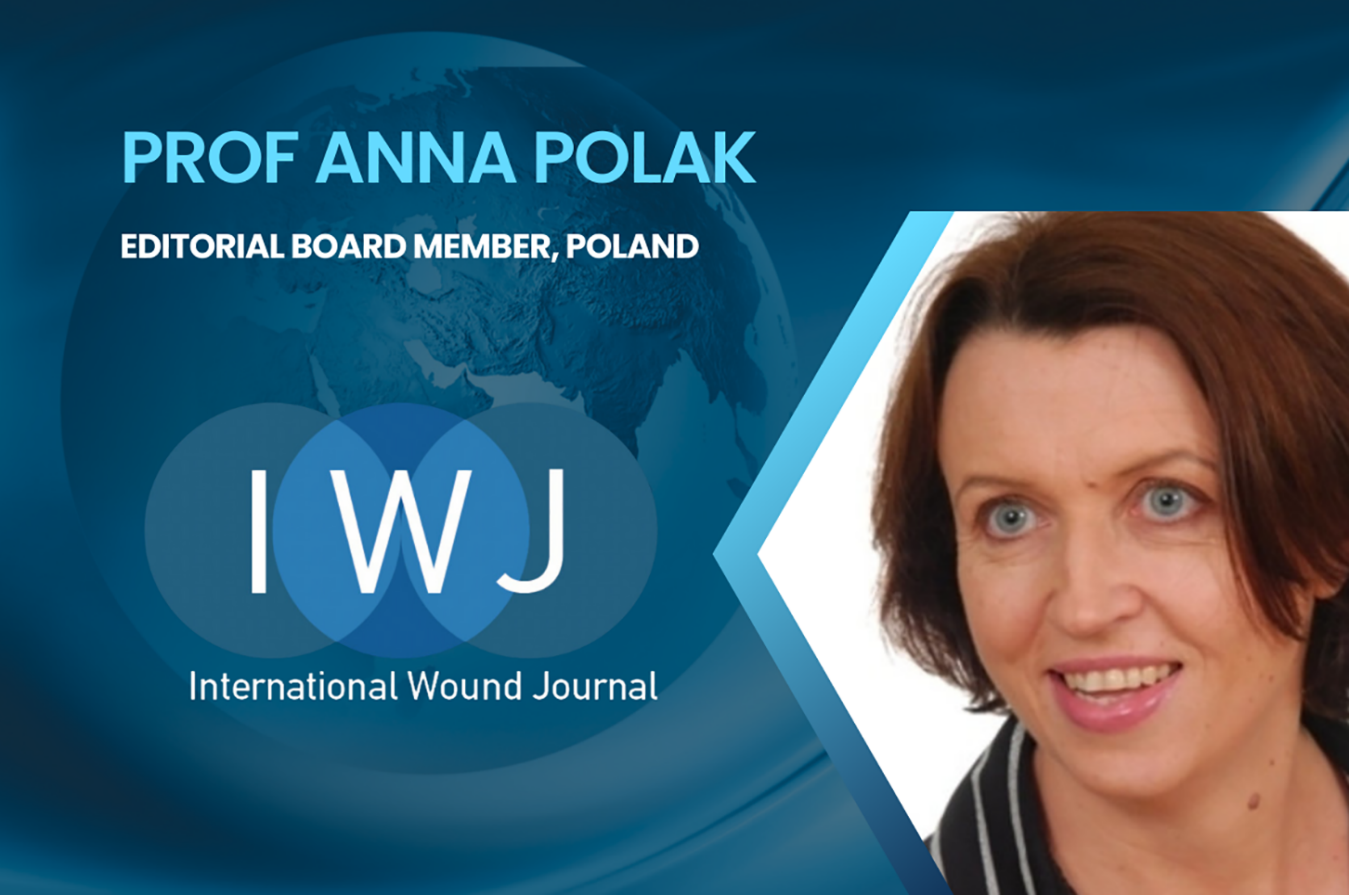	Dr. Polak works as the Associate professor, Academy of Physical Education, Department of Physical Therapy, Institution of Physical Therapy and Health Sciences, Katowice, Poland, and is the Head of the Department of Basic Clinical Physiotherapy; Dean of the Faculty of Physiotherapy, Academy of Physical Education, Academy of Physical Education, Katowice, Poland. As a university lecturer and scientist, she specializes in the treatment of chronic wounds, physical medicine and physiotherapy for elderly people (including geriatrics). Dr. Polak is the author of many scientific publications, including randomized clinical trials on physical treatment of chronic wounds and geriatric physiotherapy. As a member of several international expert groups developing clinical recommendations for the prevention and treatment of pressure ulcers, including: Prevention and Treatment of Pressure Ulcers: Clinical Practice Guideline, National Pressure Ulcer Advisory Panel & European Pressure Ulcer Advisory Panel 2009; National Pressure Ulcer Advisory Panel, European Pressure Ulcer Advisory Panel and Pan Pacific Pressure Injury Alliance—Prevention and Treatment of Pressure Ulcers: Clinical Practice Guideline; 2014; European Pressure Ulcer Advisory Panel, National Pressure Injury Advisory Panel and Pan Pacific Pressure Injury Alliance—Prevention and Treatment of Pressure Ulcers/Injuries: Clinical Practice Guideline—The International Guideline. Emily Haesler (Ed.). EPUAP/NPIAP/PPPIA: 2019. Dr. Polak was a member of the European Pressure Ulcer Advisory Panel (Trustee 2005–2011) and the Polish Wound Management Association.
**Professor Alison Porter‐Armstrong, UK**	
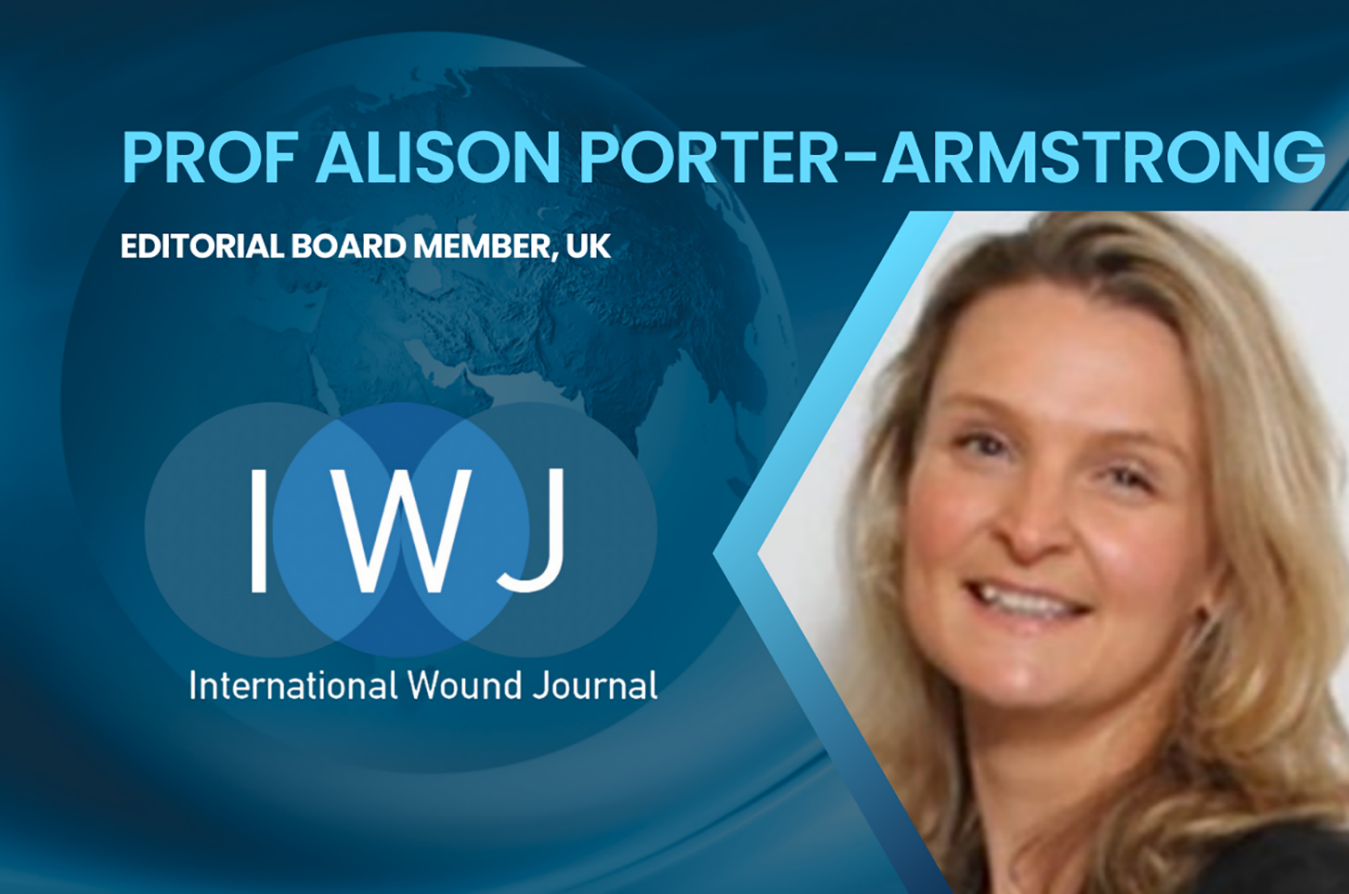	Alison is Professor of Healthcare Technology Innovation and Assessment in the School of Health and Social Care at Edinburgh Napier University. Alison is an occupational therapist whose research interests include assistive technologies, seating and postural support, pressure care, and innovations in upper limb rehabilitation and stroke care. She has published widely in these areas, authoring over 150 peer reviewed publications, commissioned research reports, keynote addresses and conference presentations. As a Cochrane Fellow, she led the Cochrane Review team on the systematic review of education of health care professionals for the prevention of pressure ulcers. Her research programme has generated significant research grant income of approximately £7 million, involving collaboration with a variety of prestigious organizations including the Royal College of Surgeons Ireland and the University of Massachusetts‐Amherst. Alison is a Trustee and Treasurer of the European Pressure Ulcer Advisory Panel (EPUAP). Alison has won many awards for her work including First Placed lead academic for the Best Knowledge Transfer Partnerships in both Northern Ireland and the United Kingdom competitions; is a recipient of the H21 Award for Services to the European Seating Symposium, and is a finalist in the Advancing Healthcare Awards UK 2024 for ‘Partnering, Leading, Shaping to Improve Population Health’.
**Professor Sebastian Probst, Switzerland**	
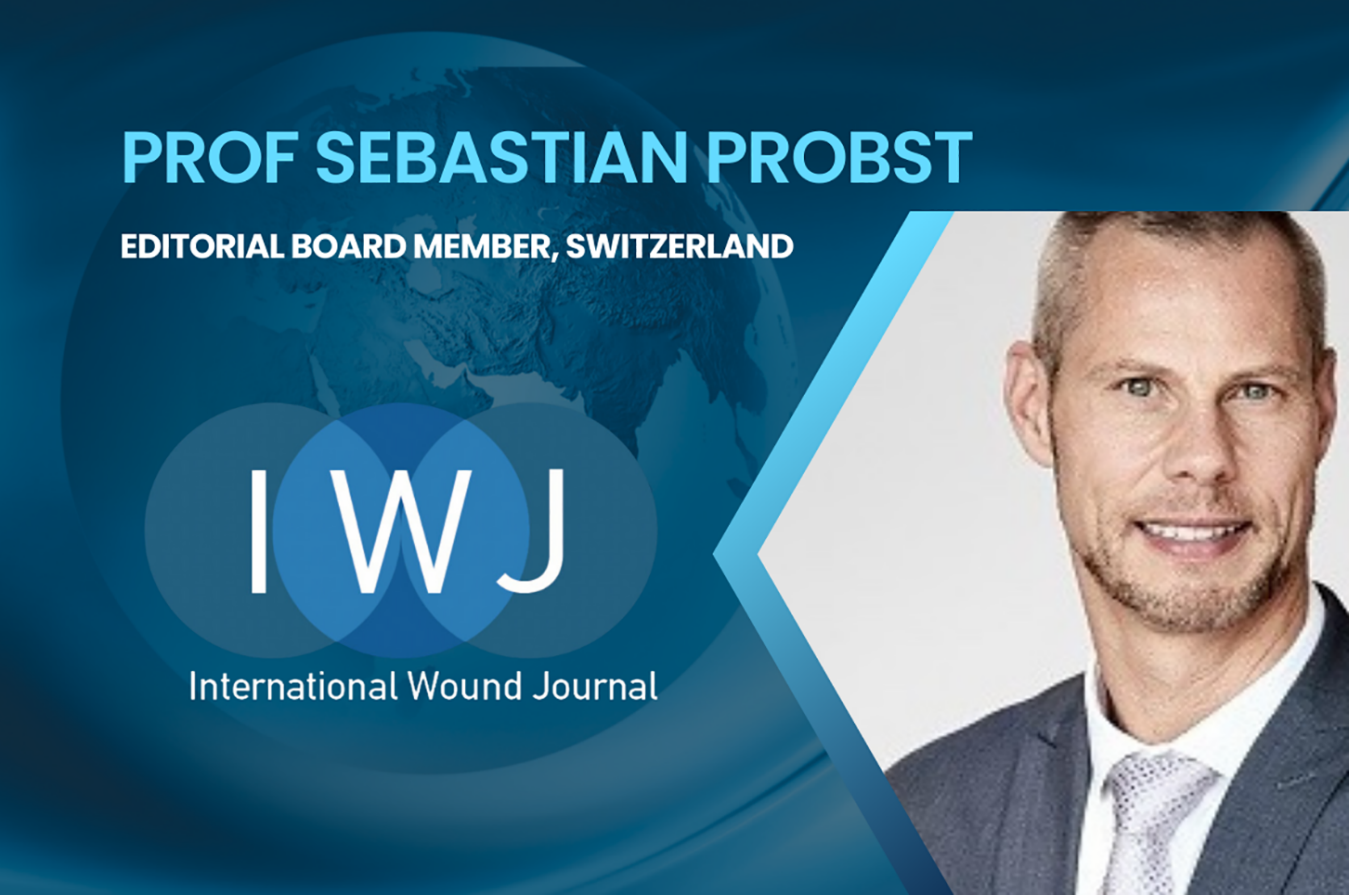	Sebastian Probst holds the position of Full Professor of Tissue Viability and Wound Care at the HES‐SO University of Applied Sciences and Arts Western Switzerland, and the University Hospital of Geneva. He is also Adjunct Professor at Monash University in Melbourne, Australia and the University of Galway in Galway, Ireland. Sebastian's research contributes to a person‐centred care approach to develop best‐practice wound care, patient education, social support and self‐efficacy in wound patients and their families. He is currently engaged in the development of artificial intelligence in wound care to enhance the decision‐making process of healthcare professionals, ultimately improving patient and family outcomes. He actively lectures and mentors Masters and PhD students. He has collaborative projects nationally and internationally. He serves as the Immediate Past President of the European Wound Management Association and as Vice‐President of WundD.A.CH, where he plays pivotal roles in project and document development within these associations.
**Professor Rytis Rimdelka, Luthuania**	
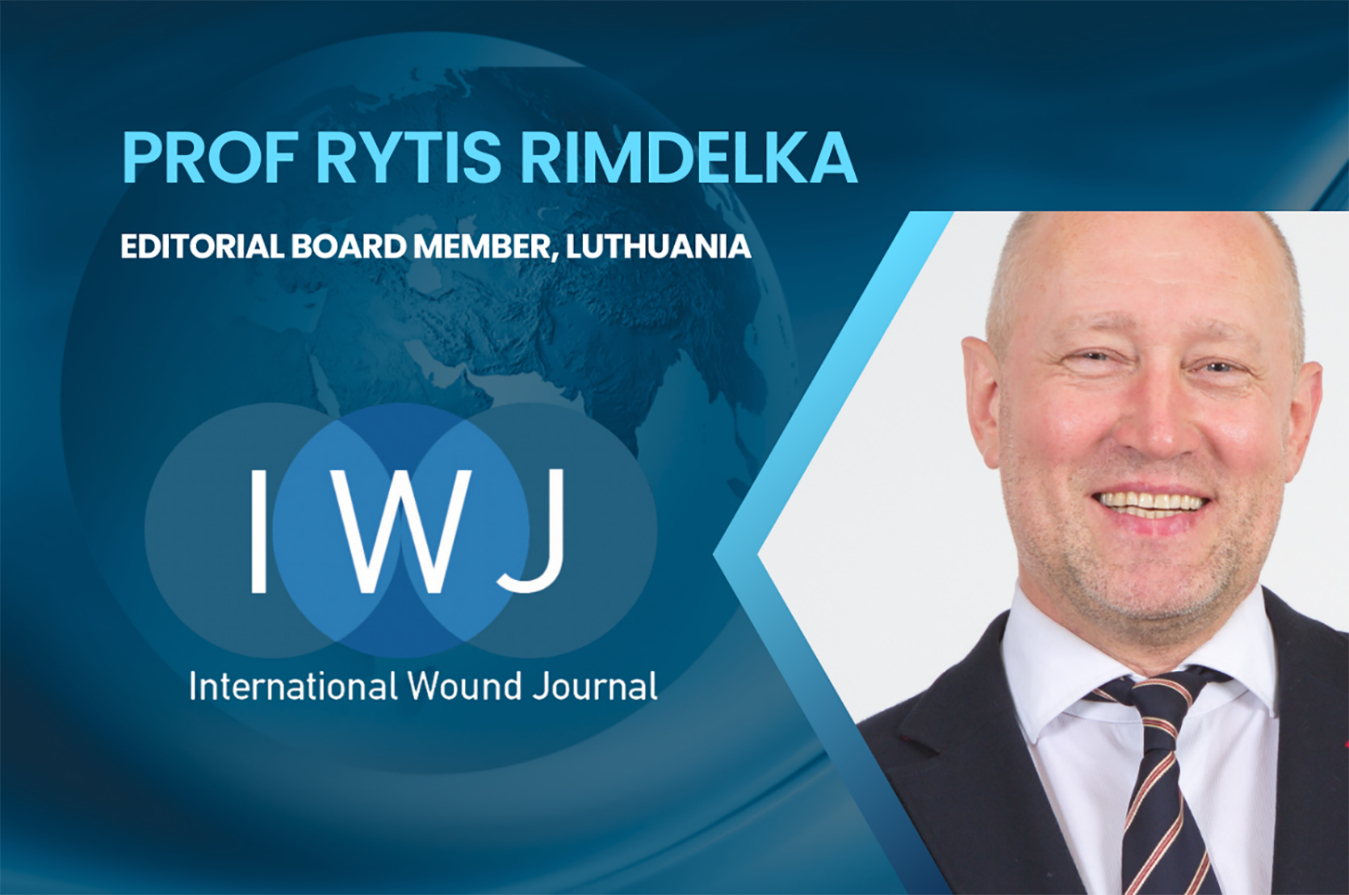	Prof. Rimdeika (MD, PhD) is a renowned Lithuanian medical doctor, scientist and professor specializing in plastic and reconstructive surgery. He graduated from the former Kaunas Medical Academy in 1990 and has since built a notable career in both clinical practice and academia. Prof. Rimdeika served as a founder and the Head of the Department of Plastic and Reconstructive Surgery at the Lithuanian University of Health Sciences from 2006 to 2021. He has developed numerous surgical techniques and led significant research in reconstructive surgery and wound management. His contributions include over 300 lectures at international conferences and numerous publications in international scientific journals. He has also held leadership roles in various professional organizations, including the Lithuanian Wound Association, Lithuanian Plastic and Reconstructive Surgery Society, European Wound Management Association (EWMA) and European Society of Plastic, Reconstructive and Aesthetic Surgery (ESPRAS).
**Professor Ray Samuriwo, UK**	
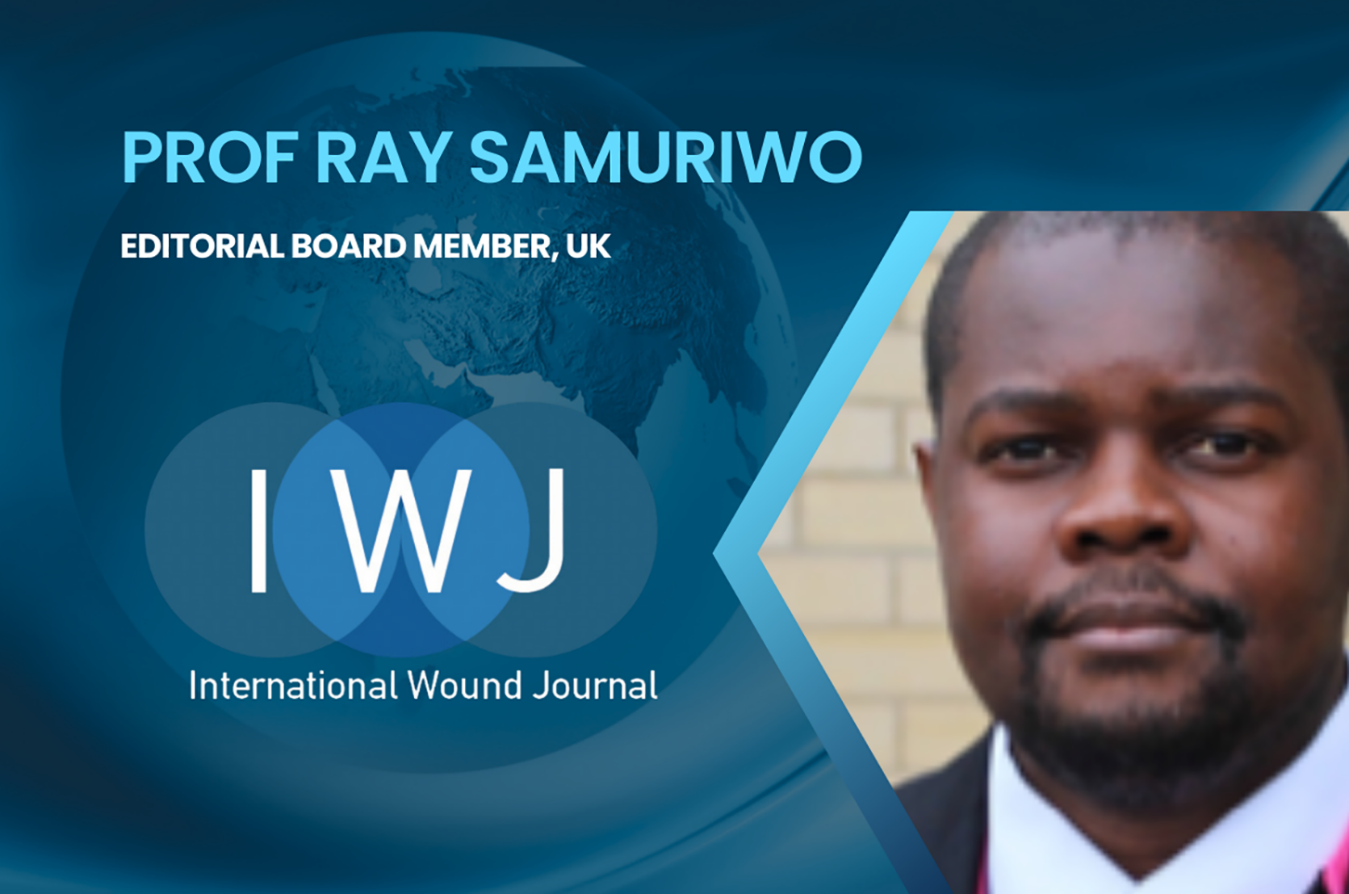	Ray is currently the Associate Professor in Nursing, School of Health and Social Care Edinburgh Napier University, Edinburgh, Scotland, United Kingdom. He is also an Honorary Senior Lecturer at the School of Medicine, College of Biomedical and Life Sciences, Cardiff University, Cardiff, United Kingdom (2022–present). Ray is an experienced researcher and teacher whose work is focused on improving the quality and safety of patient care. A key part of Ray's research is focused on improving skin health and wound healing. He has written and published more than 50 peer‐reviewed research papers, scholarly articles and books. Ray is currently a Regional Director for Europe/United Kingdom for the International Skin Tear Advisory Panel (ISTAP). He is a former Chair of the Society of Tissue Viability (SoTV) formerly known as Tissue Viability Society (TVS) from 2017 to 2019.
**Prfoessor Eric Senneville, France**	
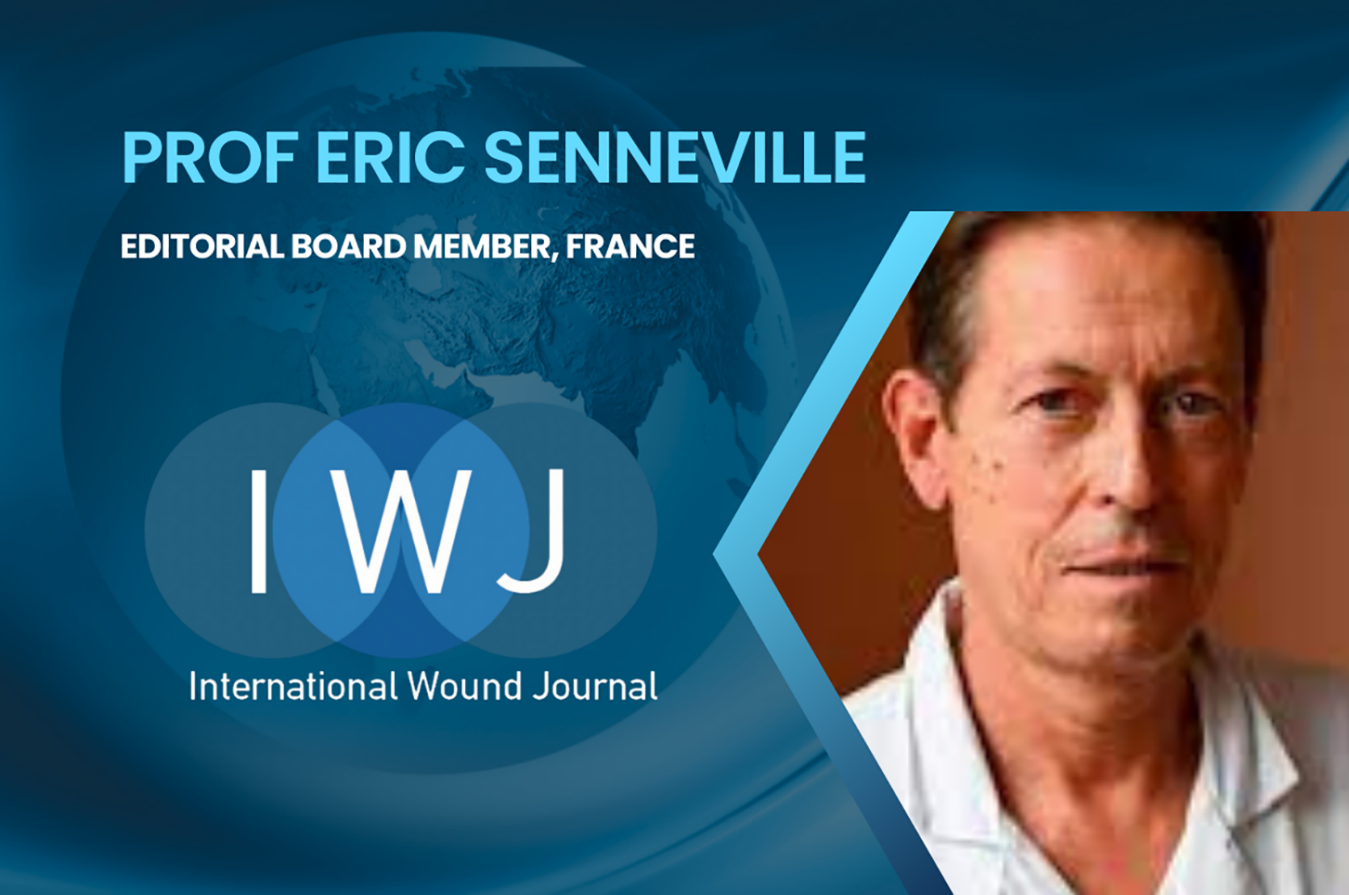	Dr. Senneville, MD, PhD, trained in medicine at Lille University, France, before specializing from 1983 to 1991 in cardiology, nephrology, internal medicine and infectious diseases. He participated in the creation of a multidisciplinary team for diabetes‐related foot infections in 1996, with a special interest in the management of diabetic patients with osteomyelitis of the foot. Dr. Senneville has been a consultant at the Department of Orthopaedic Surgery at Lille University Hospital since 1995. He is head of a 42‐bed unit in the University Department of Infectious Diseases at the Gustave Dron Hospital of Tourcoing, France, and since 2008 has been the coordinator of one of nine French national referral centres for the management of patients with complex osteo‐articular infections. Dr. Senneville is the current chair of the expert panel for the combined International Working Group on the Diabetic Foot/IDSA guidelines on the management of diabetes‐related foot infections. He has authored more than 270 indexed review articles and original articles and more than 30 textbook chapters.
**Professor Duygu Sezgin, Ireland**	
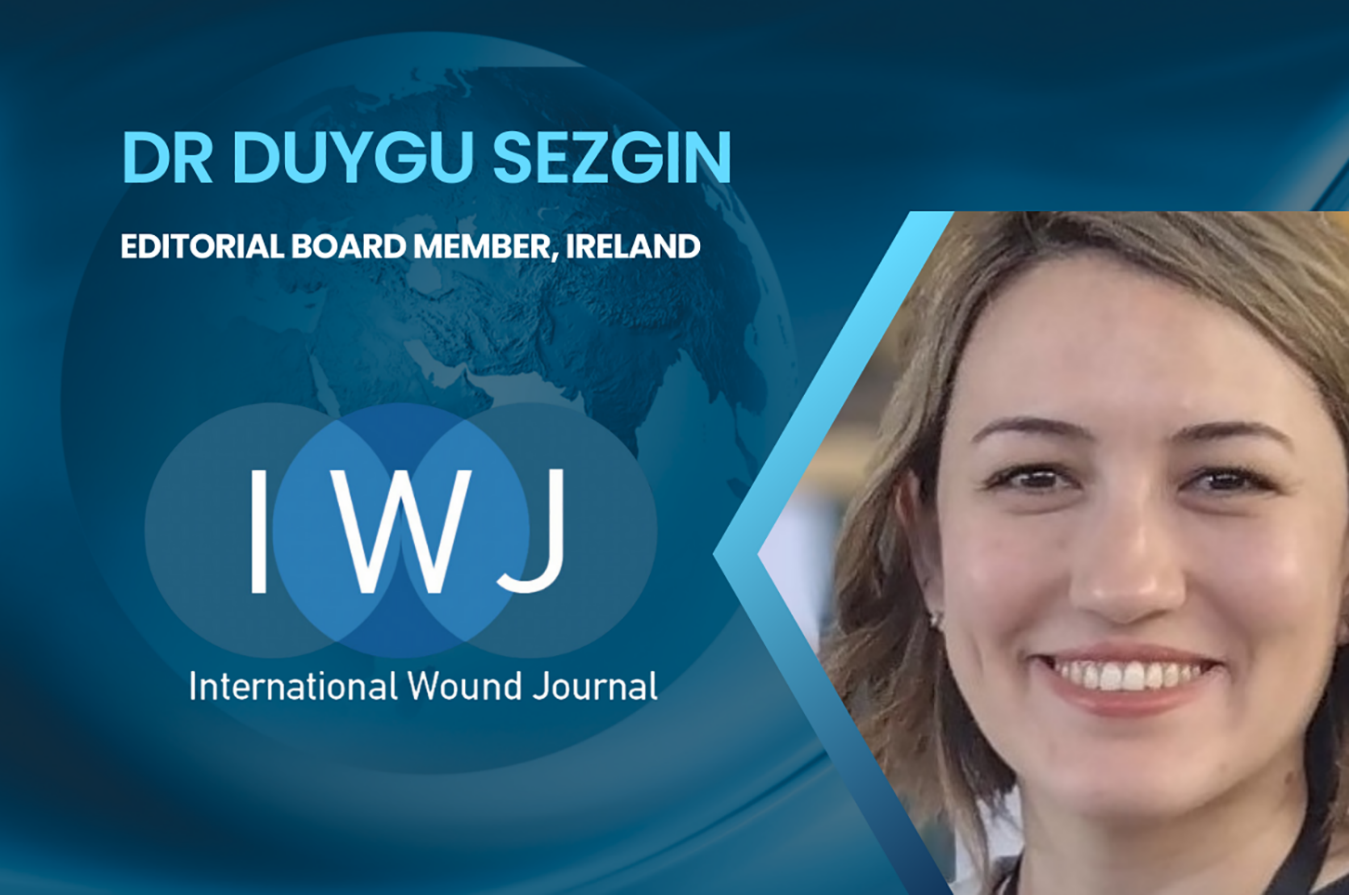	Dr. Sezgin is a lecturer in the School of Nursing and Midwifery, University of Galway. She is also the Programme Director of the Wound Healing and Tissue Repair MSc/PGDip. Dr. Sezgin has publications and research interests in systematic reviews and meta‐analyses, Delphi methodology, model‐based interventions and qualitative study designs. Her research mainly focuses on chronic disease management, including dementia, diabetes, frailty, pre‐frailty, chronic wounds, diabetic foot ulcers and venous leg ulcers.
**Debbie Sharman, UK**	
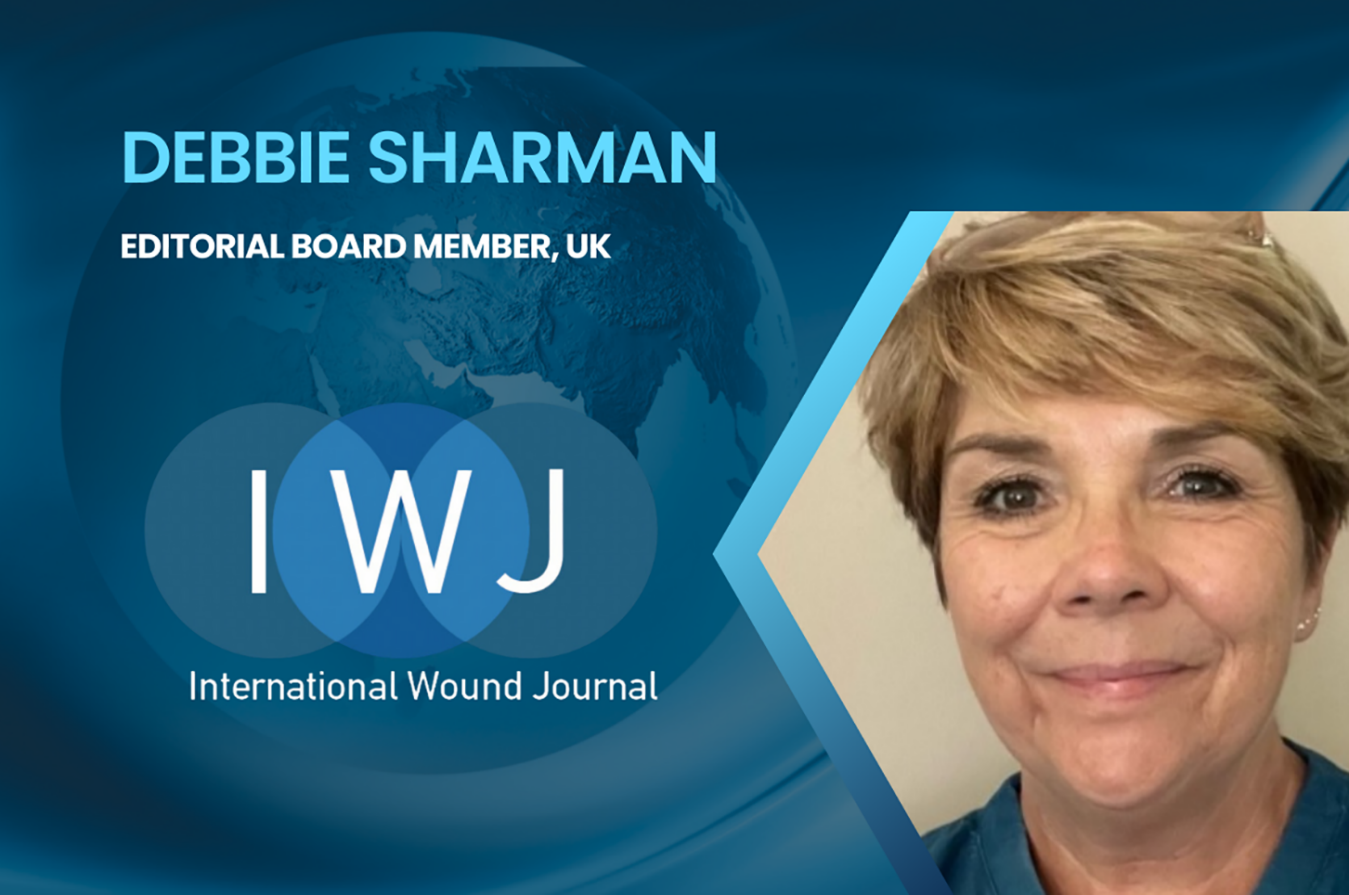	Debbie has been working in the NHS as a podiatrist since 1987, and as Consultant Podiatrist for Diabetes in Dorset since 2008. She is also the Professional lead for Podiatry in Dorset and a Visiting Lecturer at the University of Southampton. She is an Independent Prescriber, prescribing since 2010. She is a Fellow of the Faculty of Podiatric Medicine, Royal College of Physicians and Surgeons (Glasgow) and a Fellow of the Royal College of Podiatric Medicine. Additionally, she is a member of the editorial board of the Journal of Foot and Ankle Research, and co‐chair of FDUK (Foot in Diabetes UK).
**Professor Dominique Sigaudo‐Roussel, France**	
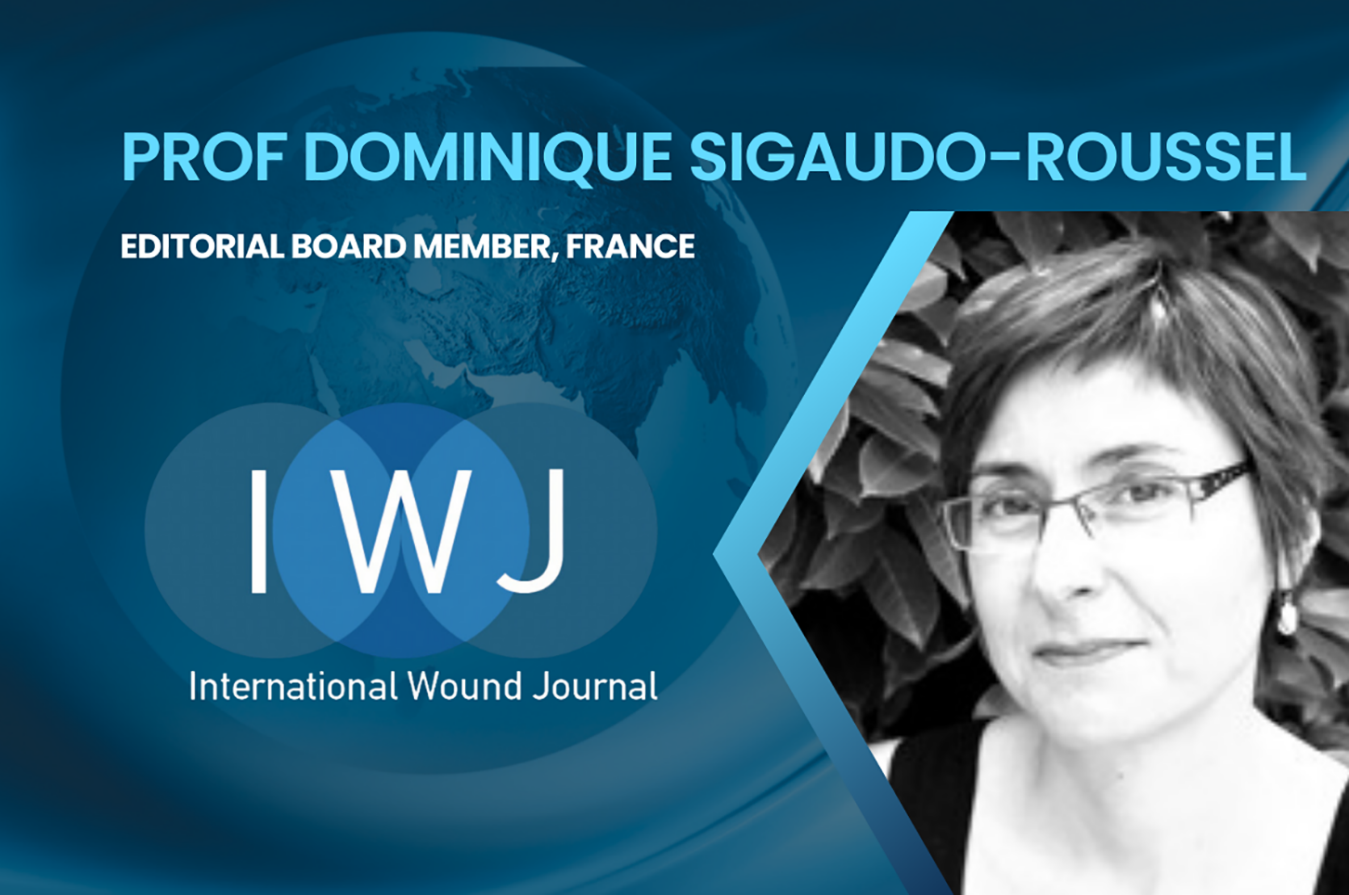	Dr. Sigaudo‐Roussel has an impressive track record of successful clinical studies, particularly in the areas of pressure ulcers related to ageing and diabetes. Currently, her research team is dedicated to investigating pressure ulcer incidence in diabetic patients. She specializes in tissue biology, with a focus on understanding tissue organization, response to stress and developing therapeutic strategies. She heads a multidisciplinary team comprising chemists, biologists, physicians, and more, with a specific focus on chronic skin lesion development and healing mechanisms.
**Professor Harm Jaap Smit, Netherlands**	
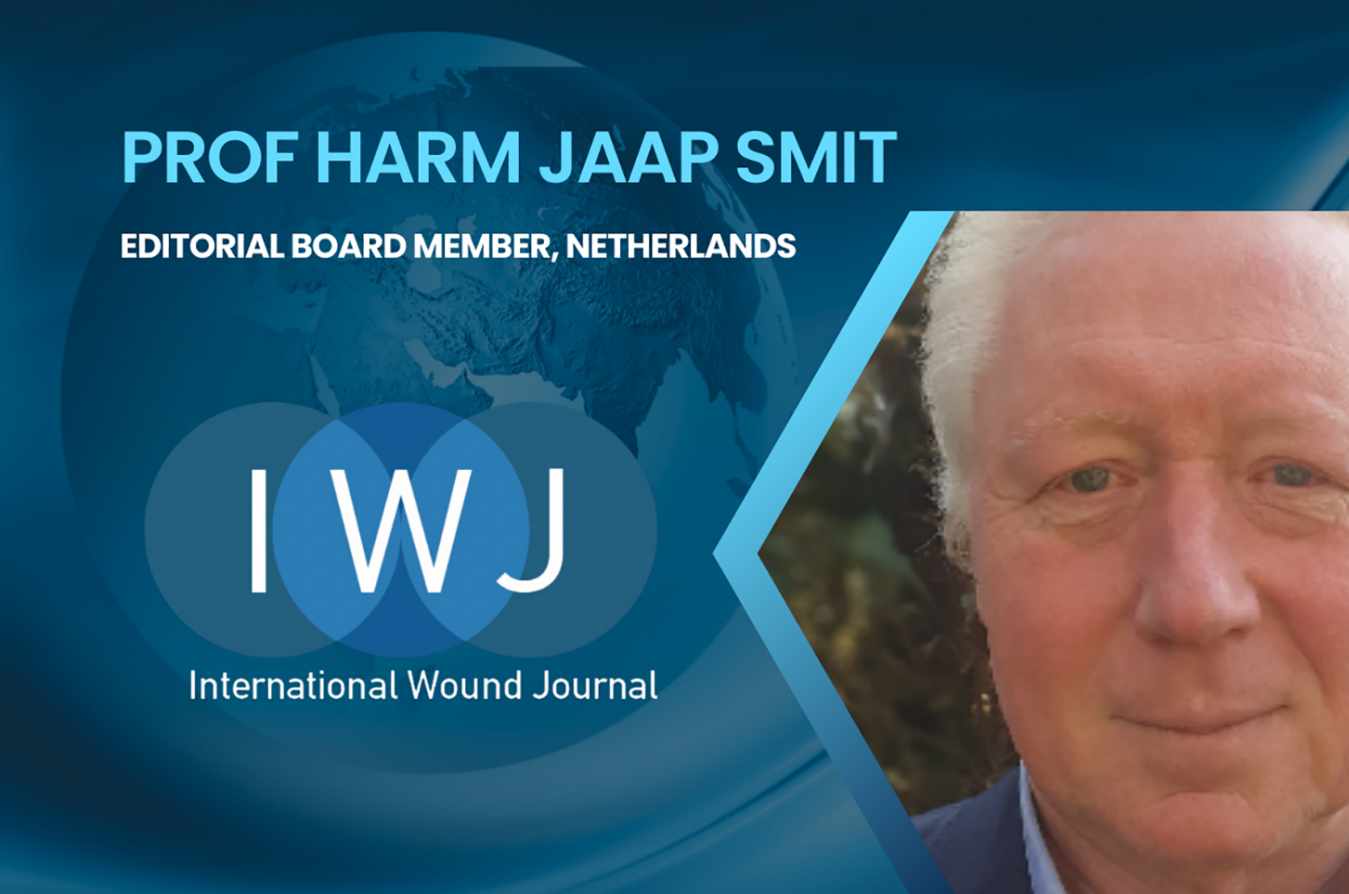	Dr. Smit studied Biology in Utrecht, The Netherlands. After finalizing his study, he has worked in the pharmaceutical and medical device industry with a strong focus on wound care. But he has always remained close to his biology roots by being active in the board of the Dutch Institute of Biology (NIBI) and the European Countries Biologists Association (ECBA). Lately he enjoys teaching Dutch wound care professionals on the fundamentals and philosophy of wound medicine and fulfils a role as scientific adviser for several companies and institutes.
**Professor Jose Verdu Soriano, Spain**	
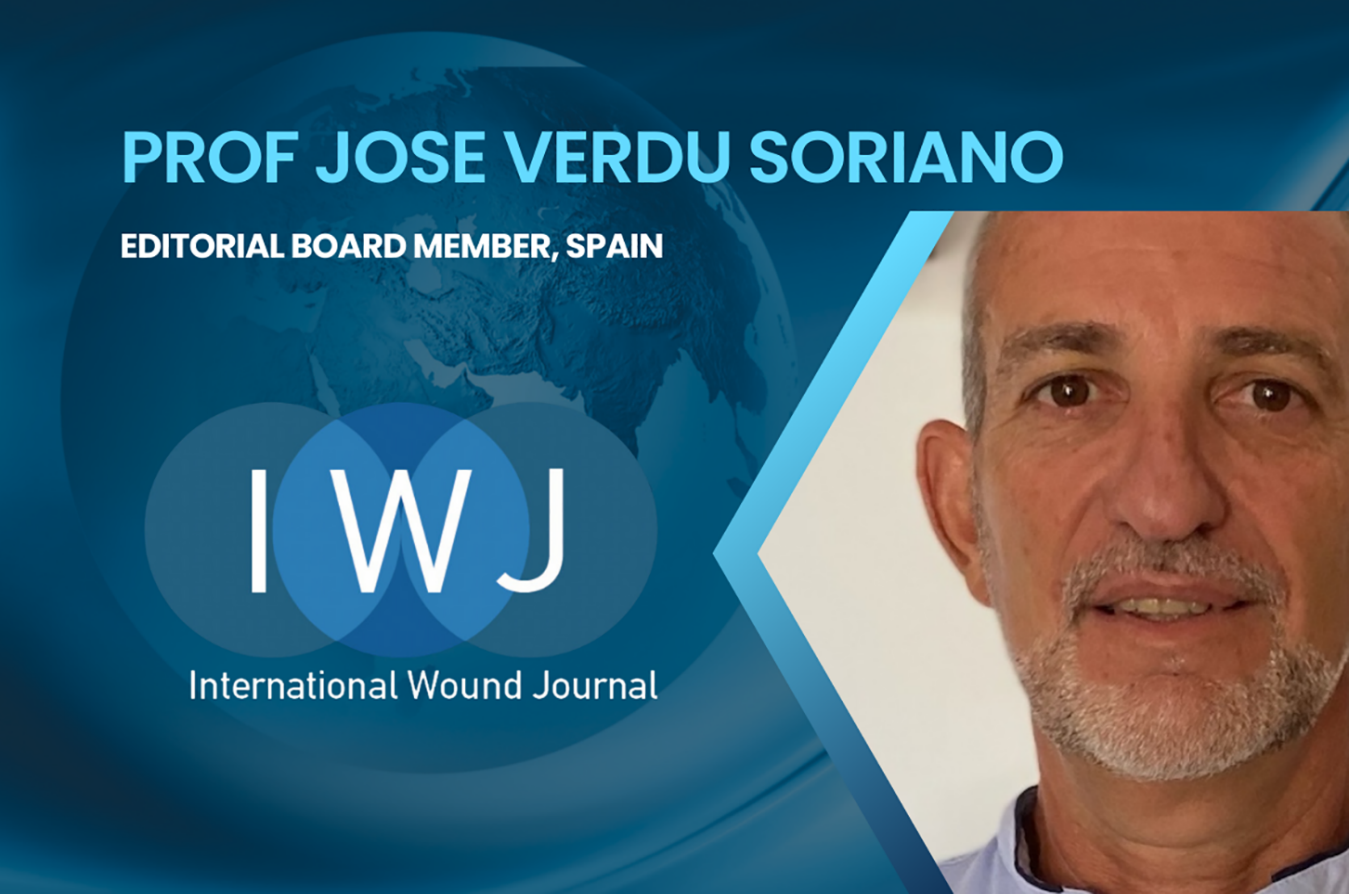	Since 1999, Jose has worked at the University of Alicante as a professor and researcher in the area of Nursing and within the Department of Community Nursing, Preventive Medicine and Public Health and History of Science, occupying different positions and currently works as a professor with five years of teaching periods and six years of research periods and a six‐year period of knowledge transfer. As a teacher, Dr. Soriano has imparted a nursing degree and a human dietetic nutrition degree, both at the University of Alicante; in addition, he has taught official master's degrees from the University of Alicante, the University of Jaen and the University of Cantabria, obtaining positive evaluations of my teaching activity in all of them. Regarding the PhD level, Dr. Soriano has participated in the PhD program since 2006 and has oriented or tutored, under his direction, 15 doctoral theses in his field of research, chronic wounds. From the research perspective, Dr. Soriano has a leadership role in the field of chronic wounds at national and international level, having held positions in the council and executive of EWMA and EPUAP until 2017. At a national level, he is part of the GNEAUPP executive and has been invited internationally both in Europe and in Latin America to teach courses and conferences in this field, not only on wounds but also in nursing research. He is a member of the Scientific Committee of EWMA for the next 2 years and has published more than 100 papers in different journals.
**Lian Stoeldraaijers, Netherlands**	
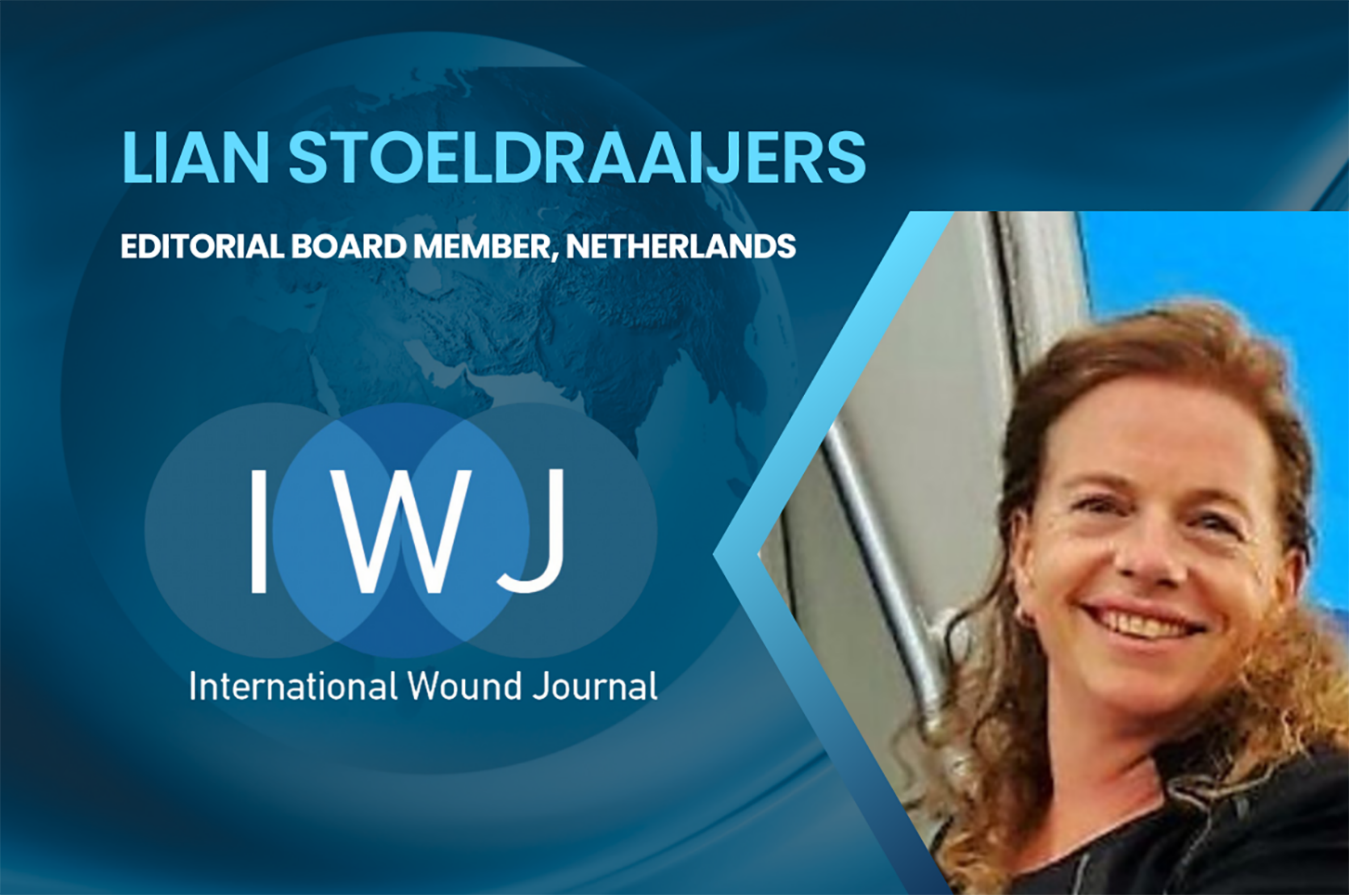	Ms Stoeldraaijers has been a private practice podiatrist for the past 38 years and has taught at the Nursing Academy, Fontys Hogescholen, Eindhoven for the past 17 years. She is the current President of the Dutch Association of Diabetes Podiatrists. She is also the National Representative on D‐Foot International and on the local organizing committee of Diabetic Foot (ISDF). Ms Stoeldraaijers is also a board member of Wound Platform Netherlands. The Wound platform Netherlands does a substantive contribution to the development and implementation of a quality standard Complex wound care for professionals and patients. Specialties: Diabetic foot care, wound care, prevention, therapeutic/custom made footwear, vascular disease in diabetes, multidisciplinary approach, primary care and specialist/hospital care, quality of life, evidence‐based practice and research.
**Helen Strapp, Ireland**	
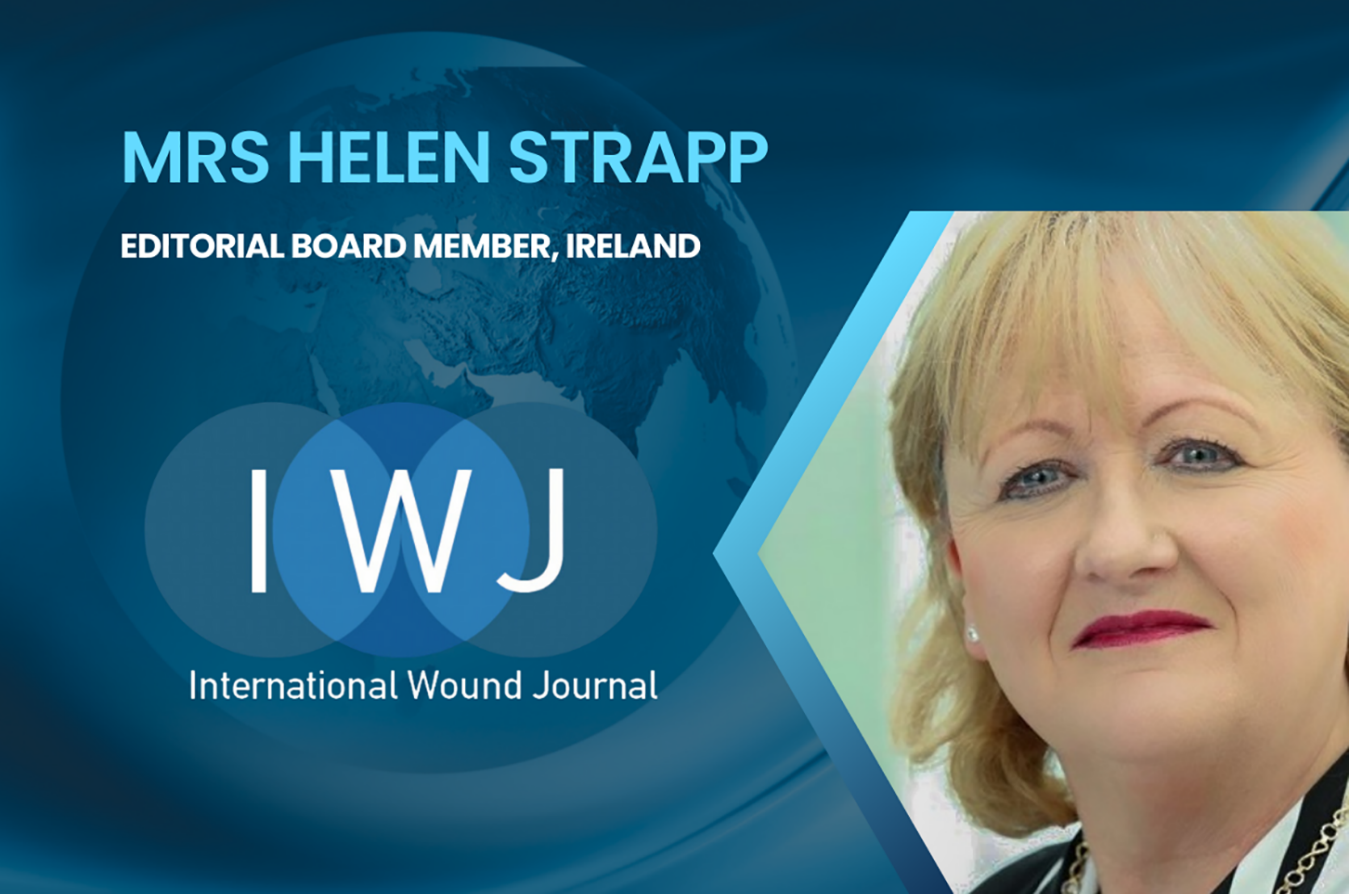	Helen is a Registered Advanced Nurse Practitioner in Tissue Viability/RNP/RGN/RSCN/P.G. and has the Dip Wound Healing & Tissue Repair and an MSc in Nursing from Tallaght University Hospital, Dublin 24, Ireland. She is the lead for pressure ulcer prevention and management in Tallaght University Hospital, which is an acute hospital with 600 beds. As an Honorary Clinical Lecturer at the Royal College of Surgeons in Ireland, she lectures on the Post Graduate Certificate/Diploma/MSc in Wound Healing & Tissue Repair. She is a member of the SWaT (Skin Wounds, and Trauma Research Centre).
**Professor Desmond Tobin, Ireland**	
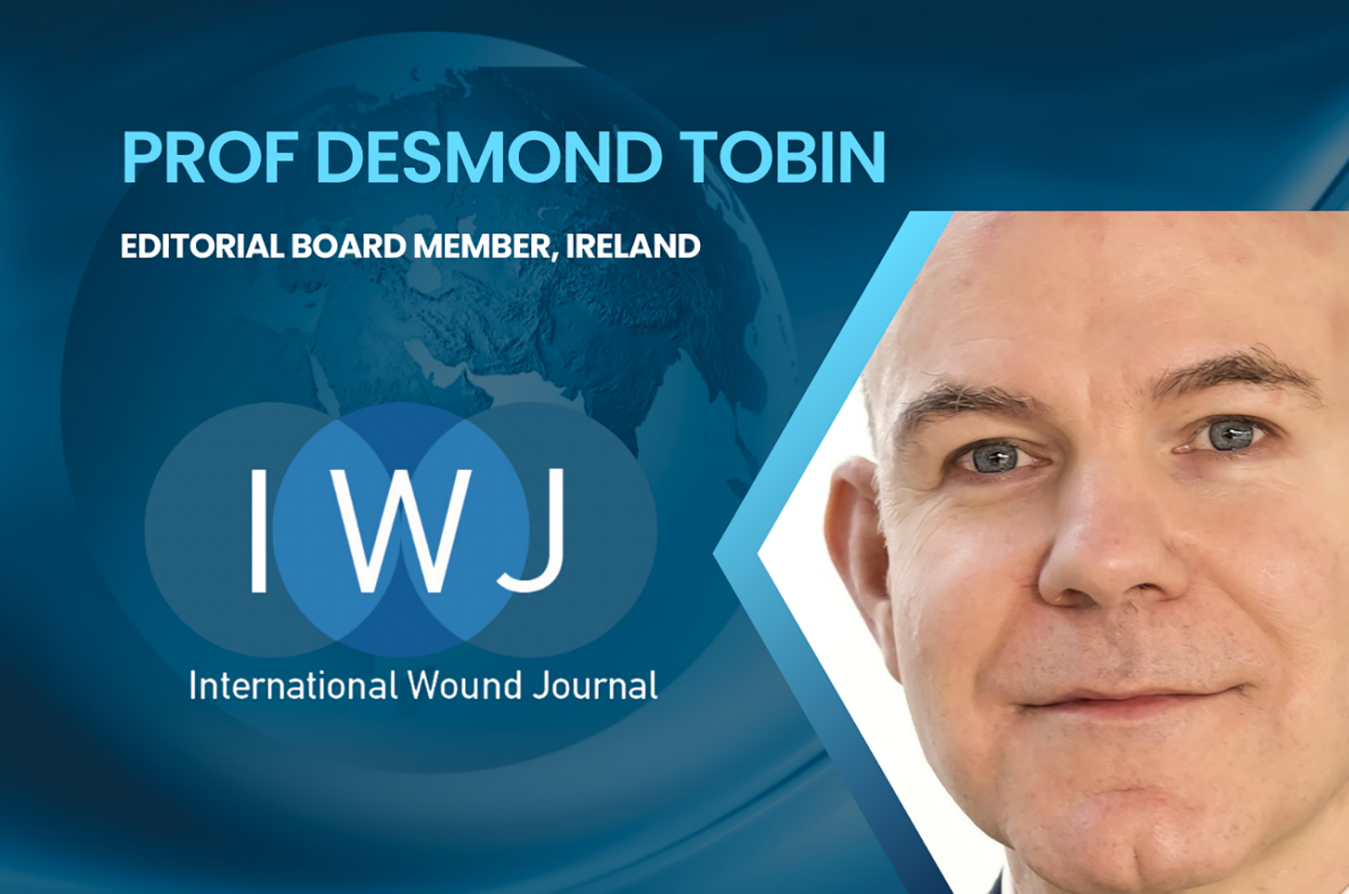	Dr. Tobin is Professor of Cell Biology and Director of the Centre for Skin Sciences at University of Bradford. He holds a BSc from the National University of Ireland (Maynooth), a PhD from the University of London (St. John's Institute of Dermatology) and post‐doctoral training from New York University Medical School's Dept. of Dermatology. Over the past 20 years, he has researched in basic and applied skin/hair sciences, with a particular focus on the biology of human melanocytes/pigmentation and hair growth disorders (immune based). He is a Fellow of Royal College of Pathologists; Royal Society of Biology, and Institute of Trichologists (Vice‐president). He has published over 140 publications, and his H‐Index is currently 53.
**Professor Joan‐Enric Torra Bou, Spain**	
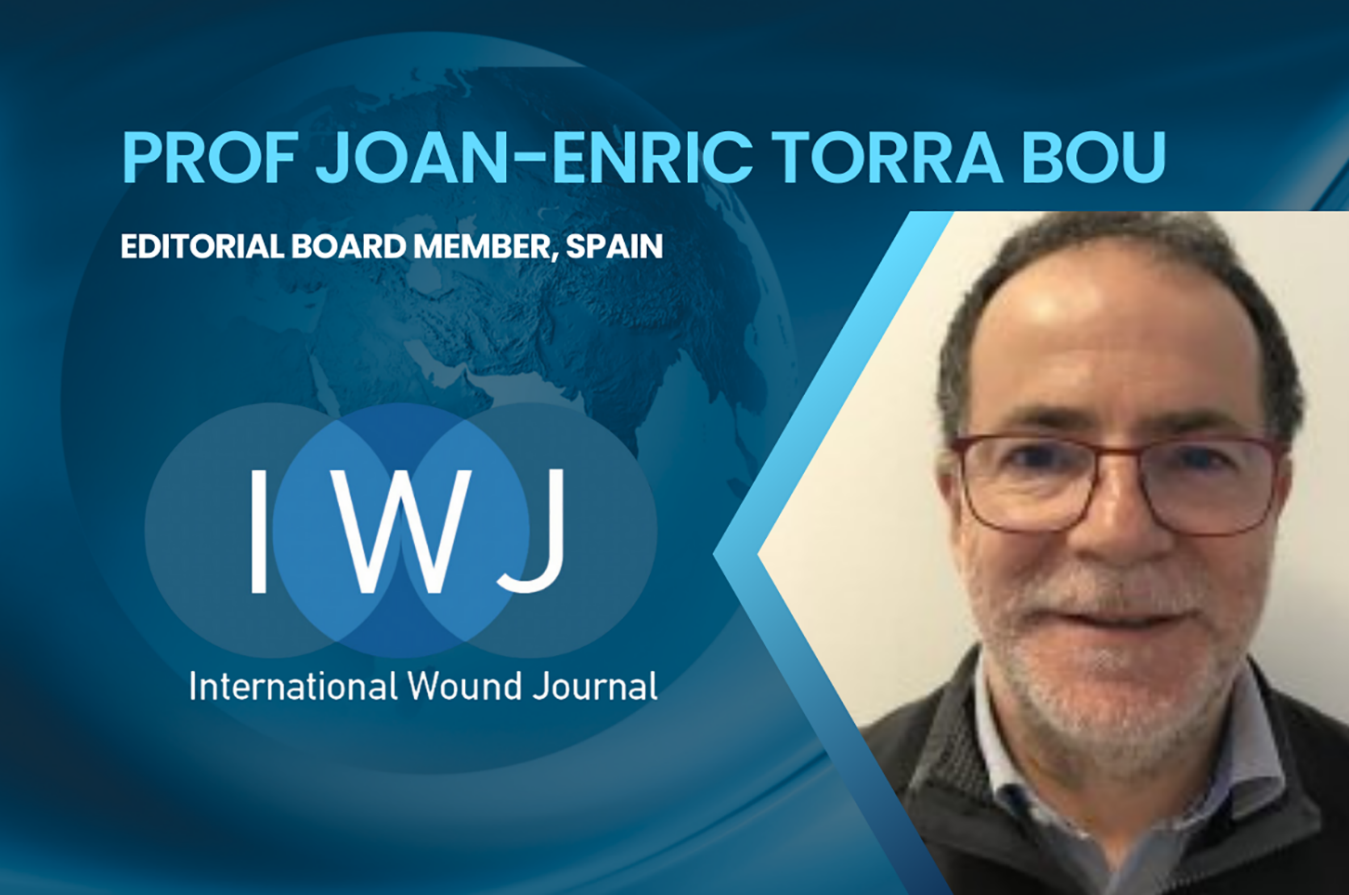	Joan Enric Torra is the founder and Managing Director of SAPIENS Ferides/as‐Wounds Consultants, the first comprehensive consultancy on wounds in Spain and Latin America and associated lecturer in the Faculty of Nursing and Physiotherapy of the University of Lleida, Igualada's campus. He got a Nursing Diploma in the University of Barcelona (Bsc Nurs) in 1982. He got a master degree in wound care (Máster sobre Investigación y Gestión del Cuidado de las Heridas) of the University of Cantabria (MSc Nurs) in 2012 and concluded his PhD at the University of Alicante (February 2016). His interests in wound care research and education are wide‐ranging, including: chronic wounds epidemiology and costs, pressure ulcers prevention and treatment, leg ulcers, complex wounds, paediatric wounds, negative pressure therapy, clinical practice guidelines assessment, Epidermolisis Bullosa, advanced skin care, incontinence associated dermatitis, (IAD), lymphedema, safety patient issues related to wound care, health economics and efficacy and efficiency in wound care and Surgical Site Infection epidemiology and prevention. He is currently a member of the steering Committee of the GNEAUPP (Spanish Pressure Ulcers and Chronic Wounds Advisory Panel), Trustee of the EPUAP (European Pressure Ulcer advisory Panel), and member of the National Pressure Injury Advisory Panel (NPIAP). He has been vice president of the GNEAUPP (1997–2003) www.gneaupp.org and member of the boards of the European Pressure Ulcer Advisory Panel (1998–2003) and the European Wound Management Association (1999–2003). He currently lectures as associated professor at the Faculty of Nursing and Physiotherapy at the University of Lleida, is lecturer at the official wound care master course at the University of Cantabria and in the post‐graduate wound care programmes of the Universitat de Lleida, Universitat Rovira I Virgili (Tarragona), the Escola Superior de Ciencies de la Salut Tecnocampus (Universitat Pompeu Fabra) in Mataró, the Campus Docent Sant Joan de Déu in Barcelona and in the Universitat Autónoma de Barcelona. He has published more than 160 scientific papers, including more than 100 articles on chronic wounds and related scientific topics (15 of them in international journals) as well as more than 50 chapters in books on Wound Care (5 of them in international works) and is the co‐editor of the two editions of the first Spanish textbook on Wound Healing.
**Mr. Joshua Totty, UK**	
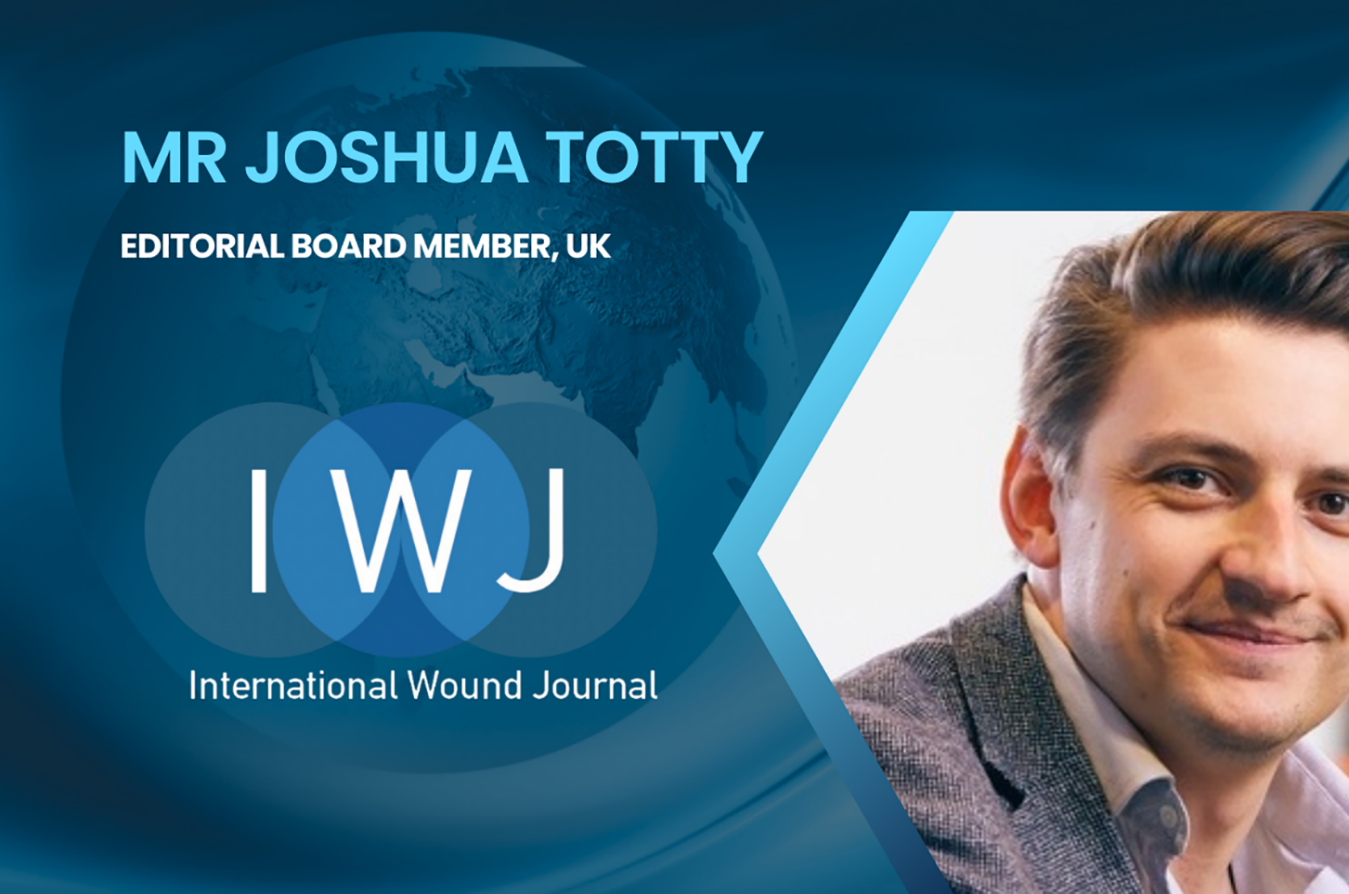	Josh is an NIHR Academic Clinical Lecturer in Plastic Surgery, conducting research into surgical wound infections, wound healing, hand surgery and skin cancer. He is also a Plastic and Reconstructive Surgery trainee, working in the Yorkshire region. After completing his undergraduate medical degree at the Hull York Medical School, he underwent foundation training in West Yorkshire. He then went on to complete a two‐year research fellowship culminating in the award of MD by Research Thesis. He was also successful in completing a Postgraduate Certificate in Health Professionals Education and meeting the requirements for admission to Fellowship of the Higher Education Academy. His doctoral research centred around prevention of surgical site infection in primarily closed wounds, through the use of active wound dressings. As part of this, he designed and implemented a pilot randomized controlled trial. The findings from this work have been presented on an international stage, and published in peer‐reviewed journals. As a result of this work, he was invited as an expert panellist to contribute to new NICE guidance on the topic. Following his research fellowship, he completed core surgical training, again in Yorkshire. This led to him successfully obtaining a national training number in Plastic Surgery in 2020. He was appointed a Clinical Lecturer in Plastic Surgery in February 2021. Since appointment, he has continued to conduct research in surgical wound infection and wound healing. He has been successful in securing grants as Principle Investigator from the Academy of Medical Sciences and from the British Association of Plastic, Reconstructive and Aesthetic Surgeons, as well as grants as co‐investigator from the Healthcare Infection Society and the National Institute for Health Research. In addition to surgical site infection and wound healing, other areas of research interest include skin cancer and the surgical management of skin cancers, hand surgery and soft tissue infection. He sits on the executive committee of the Reconstructive Surgery Trials Network as vice‐lead, and collaborates closely with departments both within the University and externally.
**Professor Peter Vogt, Germany**	
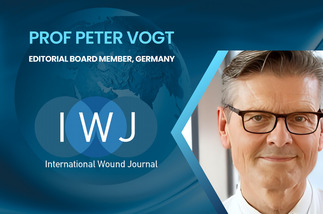	Prof. Vogt is a highly experienced plastic and aesthetic surgeon with over 40 years of expertise. He has an impressive academic background, obtaining a medical licence and completing his dissertation at Goethe University Frankfurt. He further honed his surgical skills through extensive training and fellowships at renowned institutions such as Harvard Medical School and the Brigham and Women's Hospital in Boston. Dr. Vogt is currently practicing at the Department of Plastic, Aesthetic, Hand, and Reconstructive Surgery at Hannover Medical School Hospital in Germany. He has held various important positions throughout his career, including serving as the President of the European Burns Association and the German Society for Plastic, Reconstructive, and Aesthetic Surgeons. With 644 scientific publications, Dr. Vogt is actively involved in research and has significantly contributed to plastic surgery. His publications cover a wide range of topics, including autologous fat transplantation, wound closure techniques and treatment of burns. Dr. Vogt's extensive experience, exceptional academic credentials and commitment to advancing the plastic and aesthetic surgery field make him a highly respected and sought‐after professional. His dedication to patient care and continuous research sets him apart and ensures his patients receive the highest expertise and innovative treatment options.
**Dr. Corrine Scicluna Ward, Malta**	
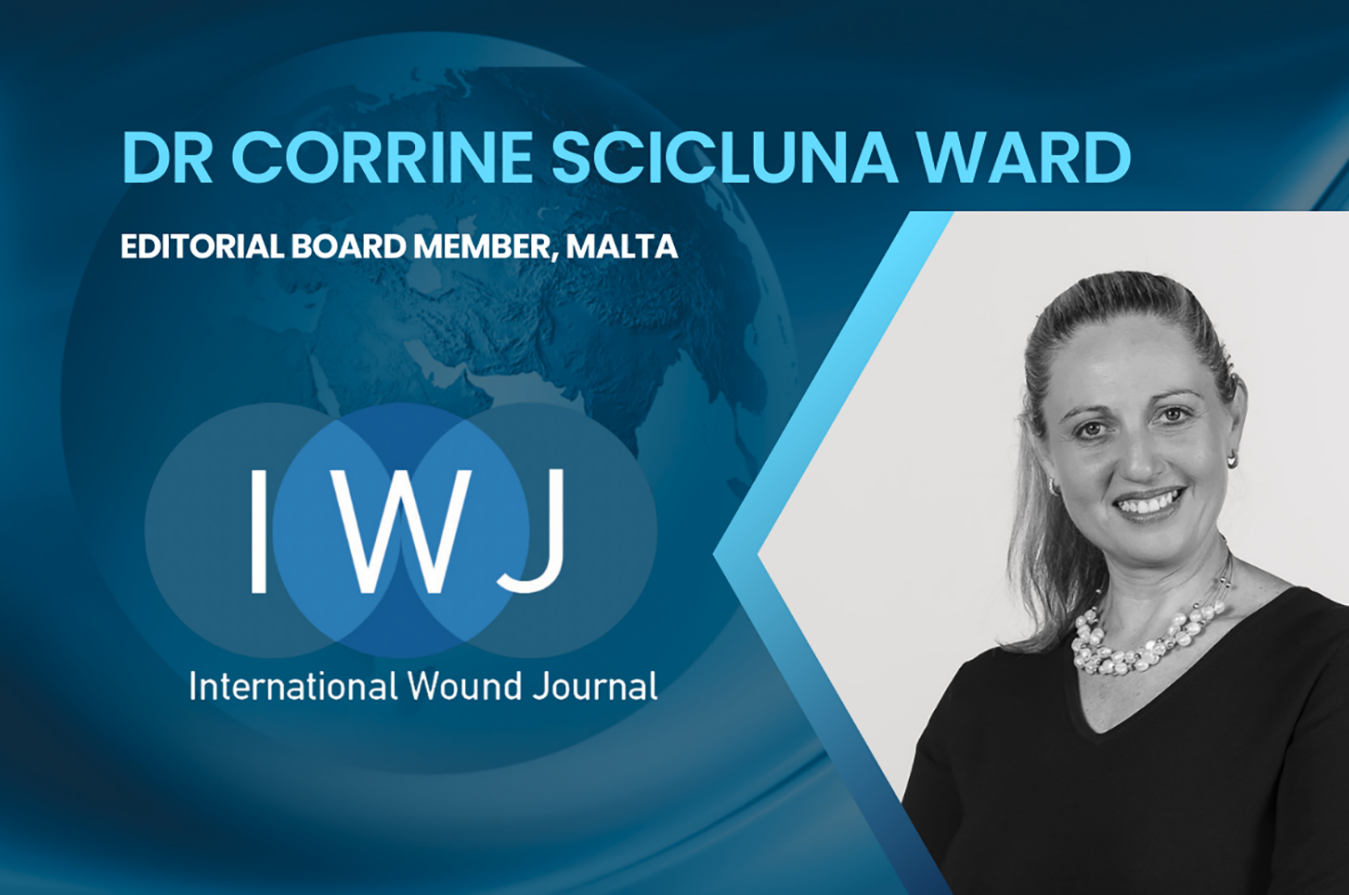	Corinne is a Senior Lecturer at the University of Malta and is the coordinator of the Master's degree in Specialist pathways, including the skin and wound care pathway. She has been a nurse for 30 years and was the first Tissue Viability Specialist Nurse who led this service in Malta. She still practices as a specialist nurse on a consultancy basis for private individuals, institutions and hospitals. Corinne is president and founder of the Maltese Association of Skin and wound Care, and is the former president of the International Skin Care Nursing Group. She is council member of the European Wound Management Association (EWMA) since May 2003. She also represents Malta on the several international organizations including the: International Diabetic Foot Group, Teacher's network in the European Wound Management Association, the Commonwealth Wound Care Resource Alliance and is a European member of the World Union of Wound Healing Societies. Corinne was a Senior Nursing Manager at Mater Dei Hospital and managed the Child and Development Assessment Unit at St Luke's Hospital. She was the president of the Nurses' Association of Malta prior to its amalgamation with the Malta Union of Midwives and Nurses (MUMN). She was the chair of the Commonwealth Nursing Federation for the European Region between 2002 and 2007. Corinne obtained a Diploma and Degree in Nursing from the University of Malta, and a Master's of Science Degree in Wound Healing and Tissue Repair from the University of Cardiff, UK, and has been awarded a PhD from Bournemouth University, UK, in 2017. Her interests include skin and wound care, pressure injuries, advanced nursing practice and legal and professional preparation of the nurse with a particular interest on the specialist nurse's role from a national policy perspective.
**Debbie Wilson, UK**	
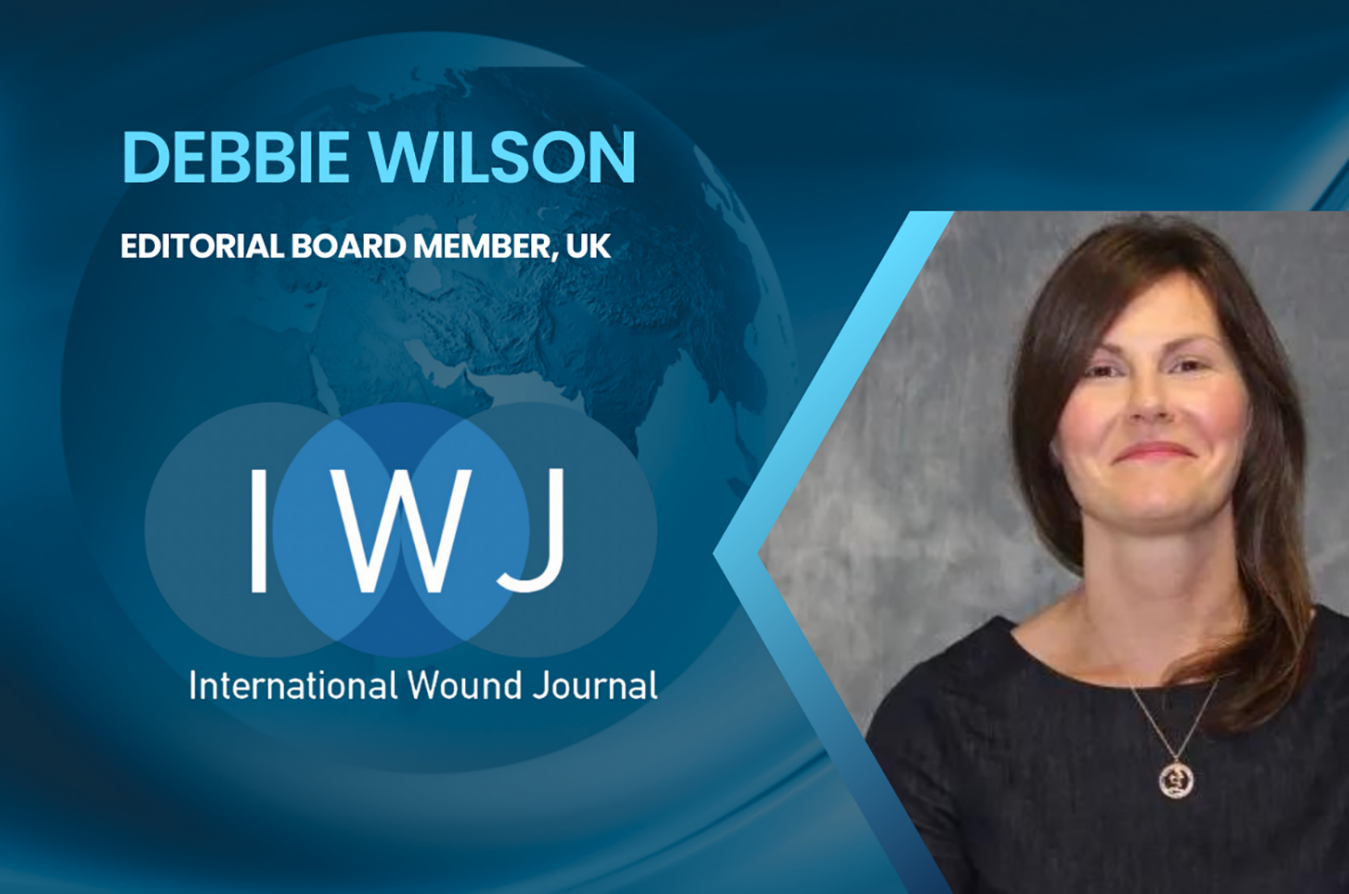	Deborah is a Fellow and ordinary Board Member for the Faculty of Podiatric Medicine's Executive Board for RCPSG. Professionally, Deborah qualified as a podiatrist in 1997 and graduated with an MSc in Podiatry in 2014 from Glasgow Caledonian University (GCU). She is currently a lecturer in Podiatry (Clinical Academic) at Glasgow Caledonian University and considers herself a lifelong learner and joined teaching in 2015 from an NHS background whereupon she developed her clinical expertise in diabetes, wound care and vascular conditions of the lower limb and foot. Her current role is split between undergraduate and postgraduate teaching, and as a podiatric clinical specialist in diabetes. The consolidation of these two roles to one post has fuelled her passion for podiatric education, which has been enabled through a collaborative and strategic partnership between GCU and NHS Lanarkshire.
**Dr. Matthew Young, UK**	
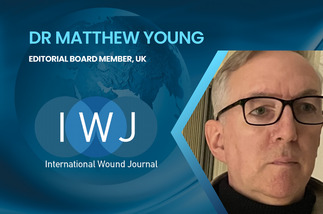	Dr. Young qualified from Newcastle University Medical School in 1985 and trained in a variety of centres including the Sheffield and Manchester diabetic foot clinics. Matthew has been a Consultant Diabetologist at the Royal Infirmary of Edinburgh since 1995, leading the multidisciplinary team of the largest diabetic foot clinic in Scotland. His research interests include foot screening and skeletal change in the diabetic foot, particularly Charcot joints and cardiovascular disease in foot ulcer patients. He served on the Scottish Intercollegiate Guidelines Network (SIGN) group, for diabetic foot guidelines, and the Foot Advisory Group of the Scottish Diabetes Group. He has published extensively on diabetes and its complications, particularly the diabetic foot. He was awarded an honorary fellowship of the College of Podiatric Medicine in recognition of his work in promoting podiatry and multi‐disciplinary working. Look out for our next editorial introducing the remainder of our new board members from Asia, South America, Oceania and Africa. As the editorial team, we are excited to expand our board to help maintain the high‐quality standards of the International Wound Journal moving forward.
